# Supplementary material for: Representativeness of a mobile phone-based coverage evaluation survey following mass drug administration for soil-transmitted helminths: a comparison of participation between two cross-sectional surveys
Source: BMJ Open. 2023 Oct 29;13(10):e070077. doi: 10.1136/bmjopen-2022-070077 (PMC10619066; doi:10.1136/bmjopen-2022-070077)
Supplement: Supplementary data [file bmjopen-2022-070077supp002.pdf]

Coverage Evaluation Survey Questionnaire

1	Name of the participant	
2	Sex if the participant	
3	Age of the participant in years	
4.1	Can the participant answer the questions now?	1 Yes, agrees to participate<br>2 Not present<br>3 No, refused<br>4 No, other<br>5 Proxy respondent
4.2	Why has household refused to participate?	1 Refused to speak/Not interested<br>2 Prior negative experience<br>3 Unwilling to provide detailed household information<br>4 Unwilling because of concerns related to risks, confidentiality, etc.<br>5 Unwilling to sign/provide thumb impression<br>6. Other
4.3	If unavailable, where is the participant?	1 At school<br>2 At college<br>3 At work<br>4 Not at home (e.g at shop)<br>5 Moved away permanently<br>6 Temporarily migrated<br>7 Died<br>8. Other
5	Name of the proxy respondent	
6	Sex of the proxy respondent	
7	Age of the proxy respondent	
8	Did someone give you a tablet in the past month to treat worms (at home, at school, or in the community)?	1 Yes<br>2 No<br>3. Don't know/Cannot recall<br>4. Refused
8.1	If 'No', Why didn't you receive the tablet?	1 Unaware of MDA<br>2 Distributor did not come to my community<br>3 Distributor came to my community but did not come to my house<br>4 Distributor did not come to my school<br>5 The distributor was out of tablets and did not return<br>6 Pregnant<br>7 Breastfeeding<br>8 Ill<br>9 Told I could not take a tablet that day for other reasons<br>10 Too old<br>11 Underage (less than 1yo)<br>12 I was busy<br>13 I was too far away<br>14 Other<br>15 Don't know/Cannot recall<br>16 Refused
8.2	If 'Yes', Did you swallow the tablet?	1 Yes<br>2 No<br>3 Don't know/Cannot recall<br>4 Refused
8.3	Why did you not swallow the tablet?	1 Pregnant<br>2 Breastfeeding<br>3 Ill<br>4 Too old<br>5 Underage (less than 1yo)<br>6 Afraid of side effects<br>7 I am not at risk for this disease<br>8 I am taking other medications<br>9 Medicine doesn't work<br>10 Other<br>11 Don't know/Cannot recall<br>12 Refused
8.4	Why did you swallow the tablet?	1 To be healthy<br>2 Afraid of getting the disease<br>3 Someone told me to<br>4 Everyone else is taking it<br>5 Don't know/Cannot recall

		6	Refused
		7	Other
9	Did you swallow the tablet in front of the person who gave it to you?	1	Yes
		2	No
		3	Don't know/Cannot recall
		4	Refused
10	Who gave you the tablet?	1	Family member
		2	Health staff
		3	Field worker
		4	Drug distributor
		5	Community leader
		6	Teacher /Anganwadi worker
		7	Other
		8	Don't know/Cannot recall
		9	Refused
11	Where did they give you the tablet?	1	At home
		2	At school
		3	At Anganwadi centre
		4	Elsewhere in community
		5	Other
		6	Don't know/Cannot recall
		7	Refused
12	Did you feel any side effects after swallowing the tablet?	1	Yes
		2	No
		3	Don't know/Cannot recall
		4	Refused
13.1	Which of the following statements are true about side effects you experienced after swallowing the tablet?	1	Mild (did not interfere with daily activities)
		2	Moderate (interfered with daily activities)
		3	Serious (required hospitalization)
		4	Don't know/Cannot recall
		5	Refused
14	How many of these tablets did you swallow?	1	1/2
		2	1
		3	2
		4	3
		5	4
		6	5+
		7	Don't know/Cannot recall
		8	Refused
15	Were you aware that tablets would be distributed?	1	Yes
		2	No
		3	Don't know/Cannot recall
		4	Refused
16	How did you know that the tablets would be distributed?	1	Family member/friend/neighbor
		2	Professional health staff
		3	Community drug distributor/community health worker/teacher
		4	Community or religious leader
		5	Brochures/flyers
		6	Posters
		7	Banners
		8	Radio
		9	TV
		10	Social media
		11	Community Theatre/Drama
		12	Gram sabha
		13	Don't know/Cannot recall
		14	Refused to answer
		15	Other
17	Did any of your neighbors take the distributed tablet?	1	Yes
		2	No
		3	Don't know/Cannot recall
		4	Refused
18	What did you like about the community treatment programme?	1	House to house treatment makes it easy
		2	Trusted distributors
		3	No long wait for drugs
		4	Free drugs
		5	No specific part I liked
		6	Don't know/Cannot recall
		7	Refused to answer

		8	Other
19	What did you not like about the community treatment programme?	1<br>2<br>3<br>4<br>5<br>6<br>7<br>8<br>9<br>10	Inconvenient time<br>Drugs ran out or were not available<br>Unfriendly distributor<br>Took too much time<br>Adverse reaction to drug<br>Did not also give treatment for other diseases<br>No specific part I disliked<br>Don't know/Cannot recall<br>Refused to answer<br>Other
20	Next time the tablets are given, how would you want them distributed?	1<br>2<br>3<br>4<br>5<br>6	The same door-to-door<br>All individuals go to school<br>Distributors in central places in the village<br>Don't know/Cannot recall<br>Refused to answer<br>Other
21	Did you have to change your daily routine to participate in the treatment day?	1<br>2<br>3<br>4	Yes<br>No<br>Don't know/Cannot recall<br>Refused
22	About how much of your time did it take to participate in the treatment day, including the time you spent waiting for the drug distributor to arrive?	1<br>2<br>3<br>4<br>5<br>6<br>7<br>8	Less than one hour<br>About one hour<br>Half of a day<br>A full day<br>More than one day, distributor did not come on day planned<br>Don’t know/Cannot recall<br>Refused to say<br>Other
23	About how many minutes did the drug distributor spend at your house on the treatment day?	1<br>2<br>3<br>4<br>5<br>6<br>7<br>8<br>9<br>10<br>11	Less than 5 minutes<br>About 10 minutes<br>About 20 minutes<br>About 30 minutes<br>About 45 minutes<br>About one hour<br>More than one hour<br>Drugs were left here when we were not home<br>Don't know/Cannot recall<br>Refused to answer<br>Other
24	If there are no adult members available, where are they?	1<br>2<br>3	Not at home (e.g., at shop)<br>Moved away permanently<br>Temporarily migrated
