# Supplementary material for: Representativeness of a mobile phone-based coverage evaluation survey following mass drug administration for soil-transmitted helminths: a comparison of participation between two cross-sectional surveys
Source: BMJ Open. 2023 Oct 29;13(10):e070077. doi: 10.1136/bmjopen-2022-070077 (PMC10619066; doi:10.1136/bmjopen-2022-070077)
Supplement: Supplementary data [file bmjopen-2022-070077supp001.pdf]

Supplementary Table 1 - Factors associated with non-response in a phone-based coverage evaluation survey, including mobile phone ownership

	Phone-based coverage survey					
	**Sampled households†**	**Non response‡**	**Univariate**		**Multivariable**	
	**N=1983 (100) n (%)**	**n=849 (42.8) n (%)**	**PR (95% CI)**	**P value**	**PR (95% CI)**	**P value**
Site details						
**Proportion of households in each arm**						
Control	993 (50.1)	429 (43.2)	REF	0.78		
Intervention	990 (49.9)	420 (42.4)	1.0 (0.9, 1.1)			
**Proportion of households per site**						
Timiri	1586 (80.0)	694 (43.8)	REF	0.45	REF	**0.04**
Jawadhu Hills	397 (20.0)	155 (39.0)	0.9 (0.7, 1.2)		0.7 (0.6, 1.0)	
Household characteristics						
**Religion**						
Other	68 (3.4)	27 (39.7)	REF	0.67		
Hindu	1915 (96.6)	822 (42.9)	1.1 (0.8, 1.5)			
**Caste**						
Higher caste	20 (1.0)	6 (30.0)	REF	0.50		
Backward caste	635 (32.0)	271 (42.7)	1.4 (0.8, 2.6)			
Most backward caste	487 (24.6)	213 (43.7)	1.5 (0.8, 2.7)			
Scheduled caste	439 (22.1)	198 (45.1)	1.5 (0.9, 2.6)			
Scheduled tribes	402 (20.3)	161 (40.0)	1.3 (0.7, 2.5)			
**House type§**						
Concrete	1054 (53.2)	417 (39.6)	REF	**0.01**		
Mixed	233 (11.7)	113 (48.5)	1.2 (1.1, 1.4)			
Government funded/ donated house	200 (10.1)	95 (47.5)	1.2 (1.0, 1.5)			
Thatched	496 (25.0)	224 (45.2)	1.1 (1.0, 1.3)			
**Socio-economic quintiles**						
Least poorest quintile	422 (21.3)	110 (26.1)	REF	**<0.01**	REF	**<0.01**
4th quintile	390 (19.7)	161 (41.3)	1.6 (1.3, 1.9)		1.5 (1.2, 1.8)	
3th quintile	406 (20.5)	185 (45.6)	1.7 (1.4, 2.1)		1.5 (1.3, 1.9)	
2th quintile	386 (19.5)	191 (49.5)	1.9 (1.5, 2.3)		1.6 (1.4, 1.9)	
Poorest quintile	379 (19.1)	202 (53.3)	2.0 (1.7, 2.5)		1.7 (1.4, 2.1)	
**Large household (family size)**						
No (<= 4 members)	1286 (64.9)	603 (46.9)	REF	**<0.01**		

Yes (>= 5 members)	697 (35.1)	246 (35.3)	0.8 (0.7, 0.8)			
**Owns mobile phone**						
No	298 (15.0)	255 (85.6)	REF	**<0.01**	REF	**<0.01**
Yes	1685 (85.0)	594 (35.3)	0.4 (0.4, 0.5)		0.5 (0.4, 0.5)	
Head of the household characteristics						
**Age**						
18-30 years	84 (4.2)	31 (36.9)	REF	**<0.01**		
31-40 years	388 (19.6)	142 (36.6)	1.0 (0.8, 1.3)			
41-50 years	516 (26.0)	185 (35.9)	1.0 (0.8, 1.2)			
51-60 years	431 (21.7)	184 (42.7)	1.2 (0.9, 1.5)			
>61 years	564 (28.4)	307 (54.4)	1.5 (1.1, 2.0)			
**Sex**						
Male	1602 (80.8)	638 (39.8)	REF	<0.01		
Female	381 (19.2)	211 (55.4)	1.4 (1.2, 1.6)			
**Education level**						
No education	587 (29.6)	317 (54.0)	REF	**<0.01**		
Any primary	452 (22.8)	196 (43.4)	0.8 (0.7, 0.9)			
Any middle	343 (17.3)	141 (41.1)	0.8 (0.6, 0.9)			
Any secondary or higher	601 (30.3)	195 (32.4)	0.6 (0.5, 0.7)			
**Marriage status**						
Never married	397 (20.0)	229 (57.7)	REF	**<0.01**		
Ever married	1586 (80.0)	620 (39.1)	0.7 (0.6, 0.8)			

Acronyms: PR - Prevalence Ratio; CI - Confidence Interval

†Number (N) of sampled households with complete covariate data used to estimate prevalence of non-response in each category.

‡Non-response - no household members available for interview, household refused to participate or unable to consent, or the household was not attempted.

§Concrete - concrete walls and roof; Mixed - concrete walls and tiled roof; Government donated/funded house - prebuilt government houses or houses funded through schemes for economically and socially marginalized groups; Thatched - thatched walls and roof.

Supplementary Table 2 - Factors associated with non-response in an in-person coverage evaluation survey, including mobile phone ownership

	In-person coverage survey					
	**Sampled households†**	**Non response‡**	**Univariate**		**Multivariable**	
	**N=1976 (100) n (%)**	**n=211 (10.7) n (%)**	**PR (95% CI)**	**P value**	**PR (95% CI)**	**P value**
Site details						
**Proportion of households in each arm**						
Control	990 (50.1)	110 (11.1)	REF	0.65		
Intervention	986 (49.9)	101 (10.2)	0.9 (0.7, 1.3)			
**Proportion of households per site**						
Timiri	1581 (80.0)	187 (11.8)	REF	**<0.01**	REF	**<0.01**
Jawadhu Hills	395 (20.0)	24 (6.1)	0.5 (0.3, 0.8)		0.5 (0.3, 0.8)	
Household characteristics						
**Religion**						
Other	58 (2.9)	13 (22.4)	REF	**<0.01**		
Hindu	1918 (97.1)	198 (10.3)	0.5 (0.3, 0.7)			
**Caste**						
Higher caste	18 (0.9)	1 (5.6)	REF	0.18		
Backward caste	609 (30.8)	73 (12.0)	2.2 (0.3, 16.0)			
Most backward caste	502 (25.4)	62 (12.4)	2.2 (0.3, 16.2)			
Scheduled caste	439 (22.2)	46 (10.5)	1.9 (0.2, 14.4)			
Scheduled tribes	408 (20.6)	29 (7.1)	1.3 (0.2, 9.8)			
**House type§**						
Concrete	1019 (51.6)	123 (12.1)	REF	0.02		
Mixed	229 (11.6)	26 (11.4)	0.9 (0.6, 1.4)			
Government funded/ donated house	191 (9.7)	24 (12.6)	1.0 (0.7, 1.5)			
Thatched	537 (27.2)	38 (7.1)	0.6 (0.4, 0.9)			
**Socio-economic quintiles**						
Least poorest quintile	391 (19.8)	48 (12.3)	REF	**0.02**		
4th quintile	360 (18.2)	39 (10.8)	0.9 (0.6, 1.3)			
3th quintile	429 (21.7)	54 (12.6)	1.0 (0.7, 1.6)			
2th quintile	410 (20.7)	47 (11.5)	0.9 (0.6, 1.5)			
Poorest quintile	386 (19.5)	23 (6.0)	0.5 (0.3, 0.8)			
**Large household (family size)**						

No<br>(<= 4 members)	1339 (67.8)	159 (11.9)	REF	**0.02**		
Yes<br>(>= 5 members)	637 (32.2)	52 (8.2)	0.7 (0.5, 0.9)			
**Owns mobile phone**						
No	293 (14.8)	25 (8.5)	REF	0.16		
Yes	167 (10.8)	1.3 (0.9, 1.8)	1.3 (0.9, 1.8)			
Data not available	134 (6.8)	19 (14.2)	1.7 (1.0, 2.8)			
Head of the household characteristics						
**Age**						
18-30 years	108 (5.5)	16 (14.8)	REF	**0.03**	REF	**<0.01**
31-40 years	403 (20.4)	55 (13.6)	0.9 (0.6, 1.5)		0.8 (0.5, 1.3)	
41-50 years	489 (24.7)	38 (7.8)	0.5 (0.3, 0.9)		0.4 (0.3, 0.7)	
51-60 years	455 (23.0)	53 (11.6)	0.8 (0.5, 1.3)		0.6 (0.4, 1.0)	
>61 years	521 (26.4)	49 (9.4)	0.6 (0.3, 1.2)		0.5 (0.3, 0.9)	
**Sex**						
Male	1567 (79.3)	155 (9.9)	REF	**0.02**		
Female	409 (20.7)	56 (13.7)	1.4 (1.1, 1.8)			
**Education level**						
No education	655 (33.1)	67 (10.2)	REF	0.36		
Any primary	427 (21.6)	48 (11.2)	1.1 (0.8, 1.5)			
Any middle	360 (18.2)	31 (8.6)	0.8 (0.5, 1.4)			
Any secondary or higher	534 (27.0)	65 (12.2)	1.2 (0.9, 1.6)			
**Marriage status**						
Never married	377 (19.1)	53 (14.1)	REF	**0.01**		
Ever married	1599 (80.9)	158 (9.9)	0.7 (0.5, 0.9)			

Acronyms: PR - Prevalence Ratio; CI - Confidence Interval

†Number (N) of sampled households with complete covariate data used to estimate prevalence of non-response in each category.

‡Non-response - no household members available for interview, household refused to participate or unable to consent, or the household was not attempted.

§Concrete - concrete walls and roof; Mixed - concrete walls and tiled roof; Government donated/funded house - prebuilt government houses or houses funded through schemes for economically and socially marginalized groups; Thatched - thatched walls and roof.

Supplementary Table 3 - Factors associated with non-response in a phone-based coverage survey, including phone number availability

Phone-based coverage survey						
	**Sampled households†**	**Non response‡**	**Univariate**		**Multivariable**	
	**N=1983 (100) n (%)**	**n=849 (42.8) n (%)**	**PR (95% CI)**	**P value**	**PR (95% CI)**	**P value**
Site details						
**Proportion of households in each arm**						
Control	993 (50.1)	429 (43.2)	REF	0.78		
Intervention	990 (49.9)	420 (42.4)	1.0 (0.9, 1.1)			
**Proportion of households per site**						
Timiri	1586 (80.0)	694 (43.8)	REF	0.45	REF	**0.08**
Jawadhu Hills	397 (20.0)	155 (39.0)	0.9 (0.7, 1.2)		0.8 (0.6, 1.0)	
Household characteristics						
**Religion**						
Other	68 (3.4)	27 (39.7)	REF	0.67		
Hindu	1915 (96.6)	822 (42.9)	1.1 (0.8, 1.5)			
**Caste**						
Higher caste	20 (1.0)	6 (30.0)	REF	0.50		
Backward caste	635 (32.0)	271 (42.7)	1.4 (0.8, 2.6)			
Most backward caste	487 (24.6)	213 (43.7)	1.5 (0.8, 2.7)			
Scheduled caste	439 (22.1)	198 (45.1)	1.5 (0.9, 2.6)			
Scheduled tribes	402 (20.3)	161 (40.0)	1.3 (0.7, 2.5)			
**House type§**						
Concrete	1054 (53.2)	417 (39.6)	REF	**0.01**		
Mixed	233 (11.7)	113 (48.5)	1.2 (1.1, 1.4)			
Government funded/ donated house	200 (10.1)	95 (47.5)	1.2 (1.0, 1.5)			
Thatched	496 (25.0)	224 (45.2)	1.1 (1.0, 1.3)			
**Socio-economic quintiles**						
Least poorest quintile	422 (21.3)	110 (26.1)	REF	**<0.01**	REF	**<0.01**
4th quintile	390 (19.7)	161 (41.3)	1.6 (1.3, 1.9)		1.5 (1.3, 1.9)	
3th quintile	406 (20.5)	185 (45.6)	1.7 (1.4, 2.1)		1.5 (1.2, 1.7)	
2th quintile	386 (19.5)	191 (49.5)	1.9 (1.5, 2.3)		1.6 (1.4, 1.9)	
Poorest quintile	379 (19.1)	202 (53.3)	2.0 (1.7, 2.5)		1.7 (1.4, 2.0)	
**Large household (family size)**						
No (<= 4 members)	1286 (64.9)	603 (46.9)	REF	**<0.01**		

Yes (>= 5 members)	697 (35.1)	246 (35.3)	0.8 (0.7, 0.8)			
**Phone number available**						
No	405 (20.4)	382 (94.3)	REF	**<0.01**	REF	**<0.01**
Yes	1578 (79.6)	467 (29.6)	0.3 (0.3, 0.4)		0.3 (0.3, 0.4)	
Head of the household characteristics						
**Age**						
18-30 years	84 (4.2)	31 (36.9)	REF	**<0.01**		
31-40 years	388 (19.6)	142 (36.6)	1.0 (0.8, 1.3)			
41-50 years	516 (26.0)	185 (35.9)	1.0 (0.8, 1.2)			
51-60 years	431 (21.7)	184 (42.7)	1.2 (0.9, 1.5)			
>61 years	564 (28.4)	307 (54.4)	1.5 (1.1, 2.0)			
**Sex**						
Male	1602 (80.8)	638 (39.8)	REF	**<0.01**		
Female	381 (19.2)	211 (55.4)	1.4 (1.2, 1.6)			
**Education level**						
No education	587 (29.6)	317 (54.0)	REF	**<0.01**		
Any primary	452 (22.8)	196 (43.4)	0.8 (0.7, 0.9)			
Any middle	343 (17.3)	141 (41.1)	0.8 (0.6, 0.9)			
Any secondary or higher	601 (30.3)	195 (32.4)	0.6 (0.5, 0.7)			
**Marriage status**						
Never married	397 (20.0)	229 (57.7)	REF	**<0.01**		
Ever married	1586 (80.0)	620 (39.1)	0.7 (0.6, 0.8)			

Acronyms: PR - Prevalence Ratio; CI - Confidence Interval

†Number (N) of sampled households with complete covariate data used to estimate prevalence of non-response in each category.

‡Non-response - no household members available for interview, household refused to participate or unable to consent, or the household was not attempted.

§Concrete - concrete walls and roof; Mixed - concrete walls and tiled roof; Government donated/funded house - prebuilt government houses or houses funded through schemes for economically and socially marginalized groups; Thatched - thatched walls and roof.

Supplementary Table 4 - Factors associated with non-response in an in-person coverage survey, including phone number availability

	In-person coverage survey					
	**Sampled households†**	**Non response‡**	**Univariate**		**Multivariable**	
	**N=1976 (100) n (%)**	**n=211 (10.7) n (%)**	**PR (95% CI)**	**P value**	**aPR (95% CI)**	**P value**
Site details						
**Proportion of households in each arm**						
Control	990 (50.1)	110 (11.1)	REF	0.65		
Intervention	986 (49.9)	101 (10.2)	0.9 (0.7, 1.3)			
**Proportion of households per site**						
Timiri	1581 (80.0)	187 (11.8)	REF	**<0.01**	REF	**<0.01**
Jawadhu Hills	395 (20.0)	24 (6.1)	0.5 (0.3, 0.8)		0.5 (0.3, 0.8)	
Household characteristics						
**Religion**						
Other	58 (2.9)	13 (22.4)	REF	**<0.01**		
Hindu	1918 (97.1)	198 (10.3)	0.5 (0.3, 0.7)			
**Caste**						
Higher caste	18 (0.9)	1 (5.6)	REF	0.18		
Backward caste	609 (30.8)	73 (12.0)	2.2 (0.3, 16.0)			
Most backward caste	502 (25.4)	62 (12.4)	2.2 (0.3, 16.2)			
Scheduled caste	439 (22.2)	46 (10.5)	1.9 (0.2, 14.4)			
Scheduled tribes	408 (20.6)	29 (7.1)	1.3 (0.2, 9.8)			
**House type§**						
Concrete	1019 (51.6)	123 (12.1)	REF	0.02		
Mixed	229 (11.6)	26 (11.4)	0.9 (0.6, 1.4)			
Government funded/ donated house	191 (9.7)	24 (12.6)	1.0 (0.7, 1.5)			
Thatched	537 (27.2)	38 (7.1)	0.6 (0.4, 0.9)			
**Socio-economic quintiles**						
Least poor quintile	391 (19.8)	48 (12.3)	REF	**0.02**		
4th quintile	360 (18.2)	39 (10.8)	0.9 (0.6, 1.3)			
3th quintile	429 (21.7)	54 (12.6)	1.0 (0.7, 1.6)			
2th quintile	410 (20.7)	47 (11.5)	0.9 (0.6, 1.5)			
Poorest quintile	386 (19.5)	23 (6.0)	0.5 (0.3, 0.8)			
**Large household (family size)**						
No (<= 4 members)	1339 (67.8)	159 (11.9)	REF	**0.02**		

Yes (>= 5 members)	637 (32.2)	52 (8.2)	0.7 (0.5, 0.9)			
**Phone number available**						
No	273 (13.8)	26 (9.5)	REF	**<0.01**		
Yes	1703 (86.2)	185 (10.9)	1.1 (0.8, 1.6)			
Head of the household characteristics						
**Age**						
18-30 years	108 (5.5)	16 (14.8)	REF	**0.03**	REF	**<0.01**
31-40 years	403 (20.4)	55 (13.6)	0.9 (0.6, 1.5)		0.8 (0.5, 1.3)	
41-50 years	489 (24.7)	38 (7.8)	0.5 (0.3, 0.9)		0.4 (0.3, 0.7)	
51-60 years	455 (23.0)	53 (11.6)	0.8 (0.5, 1.3)		0.6 (0.4, 1.0)	
>61 years	521 (26.4)	49 (9.4)	0.6 (0.3, 1.2)		0.5 (0.3, 0.9)	
**Sex**						
Male	1567 (79.3)	155 (9.9)	REF	**0.02**		
Female	409 (20.7)	56 (13.7)	1.4 (1.1, 1.8)			
**Education level**						
No education	655 (33.1)	67 (10.2)	REF	0.36		
Any primary	427 (21.6)	48 (11.2)	1.1 (0.8, 1.5)			
Any middle	360 (18.2)	31 (8.6)	0.8 (0.5, 1.4)			
Any secondary or higher	534 (27.0)	65 (12.2)	1.2 (0.9, 1.6)			
**Marriage status**						
Never married	377 (19.1)	53 (14.1)	REF	**0.01**		
Ever married	1599 (80.9)	158 (9.9)	0.7 (0.5, 0.9)			

Acronyms: PR - Prevalence Ratio; CI - Confidence Interval

†Number (N) of sampled households with complete covariate data used to estimate prevalence of non-response in each category.

‡Non-response - no household members available for interview, household refused to participate or unable to consent, or the household was not attempted.

§Concrete - concrete walls and roof; Mixed - concrete walls and tiled roof; Government donated/funded house - prebuilt government houses or houses funded through schemes for economically and socially marginalized groups; Thatched - thatched walls and roof.

Supplementary Table 5 - Phone number availability among mobile phone ownership reported in the annual census

Owns mobile phone	In-person coverage survey households (n=1563)	Phone-based coverage survey households (n=1700)	P value
Household phone number available			
**No**	102 (6.5)	176 (10.4)	**<0.01**
**Yes**	1461 (93.5)	1524 (89.6)	

Supplementary Table 6 - Factors associated with reported non-ownership of a mobile phone in Tamil Nadu, 2019

	Censused households 2019-2020	Non ownership	Univariate		Multivariable	
	N=37883 (100) n (%)	N=5728 (15.1) n (%)	PR (95% CI)	P value	PR (95% CI)	P value
Site details						
**Proportion of households in each arm**						
Control	18424 (48.6)	2800 (15.2)	REF	0.91		
Intervention	19459 (51.4)	2928 (15.0)	1.0 (0.8, 1.2)			
**Proportion of households per site**						
Timiri	29545 (78.0)	4159 (14.1)	REF	**<0.01**	REF	**<0.01**
Jawadhu Hills	8338 (22.0)	1569 (18.8)	1.3 (1.1, 1.7)		0.4 (0.3, 0.4)	
Household characteristics						
**Religion**						
Other	1133 (3.0)	93 (8.2)	REF	**<0.01**		
Hindu	36750 (97.0)	5635 (15.3)	1.9 (1.5, 2.4)			
**Caste**						
Higher	430 (1.1)	50 (11.6)	REF	**<0.01**		
Backward	11362 (30.0)	1406 (12.4)	1.1 (0.8, 1.5)			
Most backward	9207 (24.3)	1334 (14.5)	1.2 (0.9, 1.8)			
Scheduled	8457 (22.3)	1264 (14.9)	1.3 (0.9, 1.8)			
Scheduled tribes	8427 (22.2)	1674 (19.9)	1.7 (1.2, 2.4)			
**House type†**						
Concrete	20055 (52.9)	1861 (9.3)	REF	**<0.01**		
Mixed	4190 (11.1)	750 (17.9)	1.9 (1.8, 2.1)			
Government funded/ donated house	3518 (9.3)	626 (17.8)	1.9 (1.7, 2.1)			
Thatched	10120 (26.7)	2491 (24.6)	2.7 (2.4, 2.9)			
**Socio-economic quintiles**						
Least poor quintile	7573 (20.0)	132 (1.7)	REF	**<0.01**	REF	**<0.01**
4th quintile	7569 (20.0)	302 (4.0)	2.3 (1.8, 2.9)		2.0 (1.6, 2.5)	
3th quintile	7540 (19.9)	876 (11.6)	6.7 (5.3, 8.4)		5.2 (4.1, 6.5)	
2th quintile	7598 (20.1)	1750 (23.0)	13.2 (9.9, 17.7)		10.9 (8.5, 14.1)	
Poorest quintile	7603 (20.1)	2668 (35.1)	20.1 (15.4, 26.3)		19.3 (15.3, 24.3)	
**Large household (family size)**						

No (<= 4 members)	24965 (65.9)	4947 (19.8)	REF	**<0.01**		
Yes (>= 5 members)	12918 (34.1)	781 (6.0)	0.3 (0.3, 0.4)			
**Head of the household characteristics**						
**Age**						
18-30 years	1600 (4.2)	144 (9.0)	REF	**<0.01**		
31-40 years	7230 (19.1)	538 (7.4)	0.8 (0.7, 1.0)			
41-50 years	9708 (25.6)	783 (8.1)	0.9 (0.7, 1.1)			
51-60 years	8848 (23.4)	1160 (13.1)	1.5 (1.1, 1.9)			
>61 years	10497 (27.7)	3103 (29.6)	3.3 (2.5, 4.2)			
**Sex**						
Male	30800 (81.3)	3203 (10.4)	REF	**<0.01**		
Female	7083 (18.7)	2525 (35.6)	3.4 (3.1, 3.7)			
**Education level**						
No education	11581 (30.6)	3518 (30.4)	REF	**<0.01**	REF	**<0.01**
Any primary	8613 (22.7)	1240 (14.4)	0.5 (0.4, 0.5)		0.6 (0.6, 0.6)	
Any middle	6762 (17.8)	517 (7.6)	0.3 (0.2, 0.3)		0.4 (0.3, 0.4)	
Any secondary or higher	10927 (28.8)	453 (4.1)	0.1 (0.1, 0.2)		0.3 (0.3, 0.3)	
**Marriage status**						
Never married	7843 (20.7)	2750 (35.1)	REF	**<0.01**		
Ever married†	30040 (79.3)	2978 (9.9)	0.3 (0.3, 0.3)			

Acronyms: PR - Prevalence Ratio; CI - Confidence Interval

† Concrete - concrete walls and roof; Mixed - concrete walls and tiled roof; Government donated/funded house - prebuilt government houses or houses funded through schemes for economically and socially marginalized groups; Thatched - thatched walls and roof
