# Supplementary material for: Representativeness of a mobile phone-based coverage evaluation survey following mass drug administration for soil-transmitted helminths: a comparison of participation between two cross-sectional surveys
Source: BMJ Open. 2023 Oct 29;13(10):e070077. doi: 10.1136/bmjopen-2022-070077 (PMC10619066; doi:10.1136/bmjopen-2022-070077)
Supplement: Author's manuscript [file bmjopen-2022-070077.draft_revisions.pdf]

BMJ Open

BMJ Open is committed to open peer review. As part of this commitment we make the peer review history of every article we publish publicly available.

When an article is published we post the peer reviewers' comments and the authors' responses online. We also post the versions of the paper that were used during peer review. These are the versions that the peer review comments apply to.

The versions of the paper that follow are the versions that were submitted during the peer review process. They are not the versions of record or the final published versions. They should not be cited or distributed as the published version of this manuscript.

BMJ Open is an open access journal and the full, final, typeset and author-corrected version of record of the manuscript is available on our site with no access controls, subscription charges or pay-per-view fees (http://bmjopen.bmj.com).

If you have any questions on BMJ Open's open peer review process please email info.bmjopen@bmj.com

BMJ Open

Representativeness of a mobile phone-based coverage evaluation survey following mass drug administration for soil-transmitted helminths- a cross-sectional study

Journal:	*BMJ Open*
Manuscript ID	bmjopen-2022-070077
Article Type:	Original research
Date Submitted by the Author:	24-Nov-2022
Complete List of Authors:	Ramesh, Rohan; Christian Medical College Vellore, The Wellcome Trust Research Laboratory, Division of Gastrointestinal Sciences<br>Oswald, William; London School of Hygiene & Tropical Medicine, Department of Disease Control<br>Israel, Gideon ; Christian Medical College Vellore, The Wellcome Trust Research Laboratory, Division of Gastrointestinal Sciences<br>Aruldas, Kumudha; Christian Medical College Vellore, The Wellcome Trust Research Laboratory, Division of Gastrointestinal Sciences<br>Galagan, Sean ; University of Washington, Department of Global Health<br>Legge, Hugo; London School of Hygiene & Tropical Medicine, Department of Disease Control<br>PUTHUPALAYAM KALIAPPAN, SARAVANAKUMAR; Christian Medical College Vellore, The Wellcome Trust Research Laboratory, Division of Gastrointestinal Sciences<br>Walson, Judd; University of Washington, Departments of Global Health, Medicine, Pediatrics and Epidemiology<br>Halliday, Katherine; London School of Hygiene and Tropical Medicine Faculty of Infectious and Tropical Diseases, Department of Disease Control<br>Ajjampur, Sitara; Christian Medical College Vellore, The Wellcome Trust Research Laboratory, Division of Gastrointestinal Sciences
Keywords:	Public health < INFECTIOUS DISEASES, Tropical medicine < INFECTIOUS DISEASES, PUBLIC HEALTH

SCHOLARONE™
Manuscripts

I, the Submitting Author has the right to grant and does grant on behalf of all authors of the Work (as defined in the below author licence), an exclusive licence and/or a non-exclusive licence for contributions from authors who are: i) UK Crown employees; ii) where BMJ has agreed a CC-BY licence shall apply, and/or iii) in accordance with the terms applicable for US Federal Government officers or employees acting as part of their official duties; on a worldwide, perpetual, irrevocable, royalty-free basis to BMJ Publishing Group Ltd ("BMJ") its licensees and where the relevant Journal is co-owned by BMJ to the co-owners of the Journal, to publish the Work in this journal and any other BMJ products and to exploit all rights, as set out in our licence.

The Submitting Author accepts and understands that any supply made under these terms is made by BMJ to the Submitting Author unless you are acting as an employee on behalf of your employer or a postgraduate student of an affiliated institution which is paying any applicable article publishing charge ("APC") for Open Access articles. Where the Submitting Author wishes to make the Work available on an Open Access basis (and intends to pay the relevant APC), the terms of reuse of such Open Access shall be governed by a Creative Commons licence – details of these licences and which Creative Commons licence will apply to this Work are set out in our licence referred to above.

Other than as permitted in any relevant BMJ Author's Self Archiving Policies, I confirm this Work has not been accepted for publication elsewhere, is not being considered for publication elsewhere and does not duplicate material already published. I confirm all authors consent to publication of this Work and authorise the granting of this licence.

For peer review only

BMJ Open (Original article)

Title page

Representativeness of a mobile phone-based coverage evaluation survey following mass drug administration for soil-transmitted helminths: a cross-sectional study

Corresponding author

Sitara S.R. Ajjampur: The Wellcome Trust Research Laboratory, Division of Gastrointestinal Sciences, Christian Medical College, Vellore, Tamil Nadu, India-632002. Email: sitararao@cmcvellore.ac.in; Ph: +914162282052

Authors

Rohan Michael Ramesh*: The Wellcome Trust Research Laboratory, Division of Gastrointestinal Sciences, Christian Medical College, Vellore, Tamil Nadu, India

William Edward Oswald*: Department of Disease Control, Faculty of Infectious and Tropical Diseases, London School of Hygiene & Tropical Medicine, London, United Kingdom; Global Health Division, International Development Group, RTI International, Washington, USA.

Gideon John Israel: The Wellcome Trust Research Laboratory, Division of Gastrointestinal Sciences, Christian Medical College, Vellore, Tamil Nadu, India

Kumudha Aruldas: The Wellcome Trust Research Laboratory, Division of Gastrointestinal Sciences, Christian Medical College, Vellore, Tamil Nadu, India

For peer review only

Sean Galagan: Department of Global Health, University of Washington, Seattle, Washington, United States of America

Hugo Legge: Department of Disease Control, Faculty of Infectious and Tropical Diseases, London School of Hygiene & Tropical Medicine, London, United Kingdom

Saravanakumar Puthupalayam Kaliappan: The Wellcome Trust Research Laboratory, Division of Gastrointestinal Sciences, Christian Medical College, Vellore, Tamil Nadu, India

Judd L Walson: Departments of Global Health, Medicine, Pediatrics and Epidemiology, University of Washington, Seattle, Washington, United States of America

Katherine Elizabeth Halliday: Department of Disease Control, Faculty of Infectious and Tropical Diseases, London School of Hygiene & Tropical Medicine, London, United Kingdom

Sitara S.R. Ajjampur: The Wellcome Trust Research Laboratory, Division of Gastrointestinal Sciences, Christian Medical College, Vellore, Tamil Nadu, India

* These authors contributed equally to the work.

Keywords

For peer review only

Phone-based survey, coverage evaluation survey, in-person survey, low-and middle-income countries, representativeness, non-response, participation, India

Word Count: 3935

For peer review only

ABSTRACT

Objectives: With increasing mobile phone subscriptions, telephone-based surveys are gaining popularity for public health programs. Despite several advantages, systematic exclusion of participants may limit representativeness. In this study, representativeness and implementation of a phone-based coverage evaluation survey (necessitated by an abrupt lockdown during the COVID-19 pandemic) to previous in-person survey was compared. These surveys were conducted to measure treatment coverage within the DeWorm3 study, a cluster-randomized, community-based trial on community-wide mass drug administration (MDA) for soil-transmitted helminths (STH).

Design: Cross-sectional study

Setting: The DeWorm3 trial site in Tamil Nadu, India includes Timiri, a rural block, and Jawadhu hills, a hilly, hard-to-reach block inhabited predominantly by a tribal population.

Participants: In the phone-based and in-person coverage evaluation surveys, all individuals residing in 2000 randomly selected households (50 in each of the 40 clusters) were invited to participate.

Results: Of the 2000 households selected for each survey, 89% participated during the in-person survey, and 57% participated during the phone-based survey, of which 346 (17%) could not be contacted as they did not provide a telephone number during the census. Smaller households, households with lower socioeconomic status, and those with older, female or less educated household heads were under-represented in the phone-based survey. Modified Poisson regression determined non-response in the phone-based survey was higher among households from the poorest socioeconomic quintile (Adjusted Prevalence Ratios (aPR): 2.3, 95% Confidence

For peer review only

Interval (CI):2.0–2.7) and lower when households heads had completed secondary school or higher education (aPR:0.7, 95% CI:0.6–0.8).

Conclusions: These findings suggest that phone-based surveys underrepresent groups at higher risk of STH and may bias treatment coverage estimates, negatively impacting those that most need treatment. Neglected Tropical Disease control programmes may not have reliable underlying population estimates. These results suggest in-person surveys are more appropriate for evaluating coverage of MDA within most programmatic settings.

Strengths and limitations

- A detailed and up-to-date census allowed us to robustly assess the representativeness of both survey strategies.
- This analysis also offered an advantage over others based in LMICs since it was conducted in a censused population in a rural setting with regular community engagement and used the same questionnaire in both surveys within a reasonable time frame, thus allowing us to compare participation confidently.
- The phone-based survey was conducted in a state with high tele-density and fair network coverage, even in rural areas. As a result, these findings may not be generalisable to all rural settings.
- This survey was conducted during the COVID-19 pandemic and during a time of lockdowns in the study region, which may have negatively impacted responsiveness to the phone survey.

For peer review only

INTRODUCTION

Telephone-based surveys have been widely used in public health programs and research, ranging from determining immunisation coverage of children under three years, surveillance for risk factors related to leading causes of death, accessing mental health and self-reporting alcohol consumption [1-5]. With increasing mobile phone subscription rates in low-and middle-income countries (LMICs), where cell phone coverage in some regions compares with that in high-income countries (HICs), this survey method has become popular [6,7]. Cost-effectiveness, time efficiency, increased reach, willingness to share sensitive information, and data quality potentially comparable to in-person interviews are some advantages of phone-based surveys [8,9]. Disadvantages of phone-based surveys include the inability to collect visual data, potential interviewer bias, and lower response rates in some communities [9,10]. Mobile phone ownership is not universal, and the practical appeal of phone-based surveys may come at a cost by systematically excluding segments of the population, raising questions about the representativeness and generalizability of the estimates they yield [8,11–13]. India's teledensity, or the number of telephone connections for every hundred individuals living within an area, is 89, with a slight disparity between most rural and urban areas [14,15]. Tamil Nadu in southern India, with a population of 72 million, is the fifth most tele-dense state in India with 109 per 100, which is higher than the Indian average of 90%, with high mobile phone penetration even in rural households [14,16].

Neglected Tropical Diseases (NTDs) predominantly affect impoverished, rural communities, and preventive chemotherapy (PC) through mass drug administration

For peer review only

(MDA) is one of the key public health interventions implemented to control soil-transmitted helminth (STH) infections, lymphatic filariasis (LF), trachoma, schistosomiasis, and onchocerciasis [17]. The World Health Organization (WHO) recommends that coverage evaluation surveys are routinely conducted to monitor MDA programs like lymphatic filariasis using a standardised WHO tool [18,19]. For lymphatic filariasis, community-wide MDA coverage is assessed by household surveys via a standardised WHO tool [20]. For STH, the current WHO recommendation is for age-targeted deworming programs to be implemented for pre-school-aged (PSAC, 2 to 4 years) and school-aged children (SAC, 5 to 14 years) and women of reproductive age (WRA, 15 to 49 years) annually or biannually based on prevalence. Based on this recommendation, India initiated a school-based deworming program, “National Deworming Day (NDD),” biannually in 2015, targeting 240 million children [21]. Unlike LF MDA, coverage evaluation surveys in a sample of schools with a WHO tool are not typically conducted as standard following age-targeted deworming programs for STH [17].

The DeWorm3 study is an ongoing multi-country (India, Malawi, and Benin) community-based cluster-randomised trial assessing the feasibility of interrupting transmission of STH by comparing the community-wide mass drug administration (cMDA) and the standard-of-care targeted deworming of pre-school and school-going children. As a part of the trial, treatment coverage evaluation surveys were conducted after each round of six rounds of cMDA in the years 2018–2020 [22,23]. In April 2020, during the nationwide lockdown imposed by the Government of India as a control measure during the COVID-19 pandemic, a phone-based survey was conducted instead of the in-person coverage evaluation survey. Leveraging this change in the

For peer review only

study protocol necessitated by the COVID-19 pandemic, we conducted this analysis to determine the representativeness and contrast the implementation of using phone-based surveys to measure treatment coverage compared to in-person coverage evaluation surveys.

METHODS

DeWorm3 Trial

DeWorm3 is a cluster-randomised, controlled community-based intervention trial that aims to determine the feasibility of interrupting STH transmission in Benin, India, and Malawi [23,24]. In brief, 40 clusters at each study site were randomised to the standard of care, which in India is twice-yearly school-based targeted deworming of school-aged children (NDD) or the intervention which was twice-yearly community-wide MDA to all ages (cMDA, delivered door-to-door) for three years (between 2018 and 2020) (**Figure 1**) [25]. At the start of each of the three years, an annual population census was conducted to obtain accurate population data at the individual and household level and optimise coverage estimates during the MDAs. Following each of the six rounds of cMDA, coverage evaluation surveys were conducted in the intervention and control clusters within a week of cMDA. The most recent census served as the sampling frame for household selection for the coverage evaluation survey. All census and coverage evaluation surveys were collected using the same electronic data collection forms programmed into the SurveyCTO mobile application (Dobility, Inc; Cambridge, MA and Ahmedabad, India) run on encrypted Android smartphones. The survey forms in this application were embedded with logic checks, skip logic,

For peer review only - http://bmjopen.bmj.com/site/about/guidelines.xhtml

numerical constraints and pre-loaded with locating and demographic information to reduce errors while entering data.

Study setting

The DeWorm3 trial site in India includes a sub-site in Timiri, a rural area in Ranipet district (formerly a part of Vellore district), and a subsite in Jawadhu hills, a hilly, hard-to-reach area inhabited predominantly by tribal groups in Tiruvannamalai district. Both sub-sites are located in the southern state of Tamil Nadu. The study covers an annually enumerated population of nearly 141,000, residing in approximately 37,000 households, with a total area of 477 km^2 [22]. Males and females are equally represented, and children below 15 years of age constitute 23% of the population. Nearly a third of the adults received no formal education, with 20% and 90% of the population engaged in agricultural activities in Timiri and Jawadhu hills, respectively [23]. More details on India's study site and population have been published previously [22].

Coverage evaluation surveys

For the coverage evaluation surveys, 50 households were randomly selected to participate in the 40 clusters (n=2000). Questions in the survey aligned with the WHO-endorsed coverage evaluation survey for use by national programs, including individual-level questions on receipt and swallowing of the drug and reasons for not taking treatment if applicable (**Supplementary file**) [18]. The in-person coverage evaluation survey in 2019 was conducted from 27th to 31st August, based on a household sampling frame derived from the previous annual census (25th October 2018 to 2nd February 2019) **(Figure 1).** All households had to be visited up to three

For peer review only

times before they were considered unavailable, and the reasons were recorded. Sampled households that were not located or unavailable were substituted as necessary with households from two randomly-selected staged replacement lists of 20 households per cluster. First, the respondent answered the household-level questions, and then each member present was interviewed. Proxy responses from household members were accepted for children under five years and household members absent on the second or third visit.

The phone-based coverage evaluation survey was conducted from 31st March to 10th April 2020 with a household sampling frame derived from the previous annual census (19th October 2019 to 31st January 2020) (**Figure 1**). All households were eligible for selection, regardless of whether they had provided a phone number during the census. Households without a telephone number, who did not consent, or whose number was not reachable (two attempts made four hours apart were made on the first day and the third and fourth attempts made the next day) were also substituted with households from replacement lists. The interviewers were trained to administer the coverage evaluation survey over the phone while recording the responses on the SurveyCTO mobile application on another device. The next available adult was interviewed using the same phone number if the head of the household (preferred respondent) was unavailable for three attempts. Thereafter, other household members were also interviewed over the phone during the same call. The primary respondent was allowed to provide proxy responses for children under five years and household members absent on any attempt.

For peer review only - http://bmjopen.bmj.com/site/about/guidelines.xhtml

Data analysis

To assess representativeness, we compared the characteristics of households participating in each of the coverage evaluation surveys with those of households in the previous census (among those not randomly-selected for the coverage evaluation survey) and identified determinants of non-response for each of the coverage evaluation surveys. Additional households were sampled to reach the necessary sample size, but here we only included the starting sample of 2000 households in both surveys in the analysis. We compared characteristics of households participating in each of the coverage evaluation surveys with households in the most recent previous annual census based on age, sex, reported education, and marital status of household head; household religion and caste; and time of residence and house type. Principal component analysis (PCA) using household assets was used to arrive at a composite wealth index and divided into five socioeconomic status (SES) quintiles as described previously [22]. Participants who were currently married, separated, widowed or divorced were considered ever married. Chi-squared tests were used to compare distributions of household characteristics between census and surveyed households.

In addition, we contrasted the implementation of each of the coverage evaluation surveys by comparing the number of attempts to contact (visits or calls), time to complete the survey, duration, time of calls, and in-person participation of individual members within a household using a nonparametric K-sample test of medians for continuous measures or Pearson's chi-squared test for categorical measures and compared mean duration weighted by household size using a Wald test. Statistical significance for all tests was set at p-value<0.05.

For peer review only

For each coverage evaluation survey, we identified factors associated with household non-response by fitting modified Poisson regression models with robust errors to adjust for clustering. For this analysis, a household was considered to have a response if at least one household member was present and agreed to respond to the survey. Households not meeting this criterion for any reason were considered non-response. We estimated the crude prevalence ratio (PR) between each candidate predictor variable and non-response. We then used a best-subset selection approach, modelling all possible combinations of candidate predictor variables hypothesised to influence a household's participation and selecting the final model with the smallest Bayesian Information Criterion [26]. All candidate models adjusted for sub-site and household caste were excluded from the multivariable model selection because of collinearity with subsite. In a sensitivity analysis, we examined the inclusion of mobile phone ownership and phone number availability as additional candidate predictors to determine factors associated with household non-response. As a *post hoc* analysis, we also examined the association between households who reported owning a mobile phone and phone number availability. The data were managed and analysed using STATA 16.1 software (StataCorp, TX, USA).

Patient and public involvement

Community members living in STH endemic areas were not involved in the design, conduct, reporting, or dissemination of this study.

RESULTS

For peer review only - http://bmjopen.bmj.com/site/about/guidelines.xhtml

Households available for participation in coverage evaluation surveys

During the 2019 in-person coverage evaluation survey, of the 2000 households sampled, 1780 (89.0%) participated, and during the 2020 phone-based coverage evaluation survey, 1144 (57.2%) of 2000 sampled households participated. The number of households not participating due to various reasons during the in-person and phone-based surveys is illustrated in **Figure 2**. During the phone-based survey, 346 (17.3%) households could not be contacted as phone numbers were unavailable. During the in-person survey, members were unavailable in 203 (10.1%) households even after three visits. During the phone-based survey, 505 (25.2%) households were unavailable after three or more phone calls – these included 61 households where the phone call was not picked up, 269 for whom the 'number not reachable' message was received from the phone service provider as the phone was outside an area of connection, 12 primary respondents informed the interviewers that they were 'out of town' and not present during the cMDA and as a result were unable to answer on behalf of their family members, and 163 were recorded as 'wrong number' as the call was attended by persons not associated with the surveyed household.

Representativeness of coverage evaluation surveys

No differences were observed between households' characteristics and heads participating in the in-person coverage evaluation survey with non-sampled households **(Table 1).** In the phone-based survey, however, participating households in the highest SES quintile were overrepresented (27.5%) compared to non-sampled households (19.9%), with the inverse observed in the poorest quintile ($p<0.01$). Larger households with five or more household members were also overrepresented in the phone-based survey (39.8%) compared to non-sampled households (34.0%, $p<0.01$)**.**

For peer review only - http://bmjopen.bmj.com/site/about/guidelines.xhtml

Table 1 - Comparison of household characteristics between censused and participating households of in-person and phone-based coverage surveys in Vellore, India, 2018-2020

	Census households (2018-19) N=35663 n(%)	In-person coverage survey N=1765 n(%)	P value†	Census households (2019-20) N=35900 n(%)	Phone-based coverage survey N=1134 n(%)	P value†
Site details						
**Study arm**						
Control	17420 (48.8)	880 (49.9)	0.41	17431 (48.6)	564 (49.7)	0.43
Intervention	18243 (51.2)	885 (50.1)		18469 (51.4)	570 (50.3)	
**Study sub-site**						
Timiri	27638 (77.5)	1394 (79.0)	0.14	27959 (77.9)	892 (78.7)	0.53
Jawadhu Hills	8025 (22.5)	371 (21.0)		7941 (22.1)	242 (21.3)	
Household characteristics						
**Religion**						
Other	1086 (3.0)	45 (2.5)	0.24	1065 (3.0)	41 (3.6)	0.21
Hindu	34577 (97.0)	1720 (97.5)		34835 (97.0)	1093 (96.4)	
**Caste**						
Higher caste	404 (1.1)	17 (1.0)	0.66	410 (1.1)	14 (1.2)	0.55
Backward caste	10667 (29.9)	536 (30.4)		10727 (29.9)	364 (32.1)	
Most backward caste	8533 (23.9)	440 (24.9)		8720 (24.3)	274 (24.2)	
Scheduled caste	7960 (22.3)	393 (22.3)		8018 (22.3)	241 (21.3)	
Scheduled tribes	8099 (22.7)	379 (21.5)		8025 (22.4)	241 (21.3)	
**House type***						
Concrete	18525 (51.9)	896 (50.8)	0.78	19001 (52.9)	637 (56.2)	0.13
Mixed	3975 (11.1)	203 (11.5)		3957 (11.0)	120 (10.6)	
Government donated/funded house	3380 (9.5)	167 (9.5)		3318 (9.2)	105 (9.3)	
Thatched	9783 (27.4)	499 (28.3)		9624 (26.8)	272 (24.0)	
**Socio-economic quintiles**						
Least poor quintile	7120 (20.0)	343 (19.4)	0.58	7151 (19.9)	312 (27.5)	**<0.01**
4th quintile	6965 (19.5)	321 (18.2)		7179 (20.0)	229 (20.2)	
3th quintile	7273 (20.4)	375 (21.2)		7134 (19.9)	221 (19.5)	
2nd quintile	7118 (20.0)	363 (20.6)		7212 (20.1)	195 (17.2)	
Poorest quintile	7187 (20.2)	363 (20.6)		7224 (20.1)	177 (15.6)	
**Large household (family size)**						
No (<= 4 members)	24027 (67.4)	1180 (66.9)	0.65	23679 (66.0)	683 (60.2)	**<0.01**

For peer review only

Yes (>= 5 members)	11636 (32.6)	585 (33.1)		12221 (34.0)	451 (39.8)	
Head of the household characteristics						
**Age**						
18-30 years	2143 (6.0)	92 (5.2)	0.68	1516 (4.2)	53 (4.7)	**<0.01**
31-40 years	7071 (19.8)	348 (19.7)		6842 (19.1)	246 (21.7)	
41-50 years	9103 (25.5)	451 (25.6)		9192 (25.6)	331 (29.2)	
51-60 years	7827 (21.9)	402 (22.8)		8417 (23.4)	247 (21.8)	
>61 years	9519 (26.7)	472 (26.7)		9933 (27.7)	257 (22.7)	
**Sex**						
Male	28260 (79.2)	1412 (80.0)	0.44	29198 (81.3)	964 (85.0)	**<0.01**
Female	7403 (20.8)	353 (20.0)		6702 (18.7)	170 (15.0)	
**Education level**						
No education	12047 (33.8)	588 (33.3)	0.55	10994 (30.6)	270 (23.8)	**<0.01**
Any primary	7604 (21.3)	379 (21.5)		8161 (22.7)	256 (22.6)	
Any middle	6210 (17.4)	329 (18.6)		6419 (17.9)	202 (17.8)	
Any secondary or higher	9802 (27.5)	469 (26.6)		10326 (28.8)	406 (35.8)	
**Marriage status**						
Never married	6778 (19.0)	324 (18.4)	0.50	7446 (20.7)	168 (14.8)	**<0.01**
Ever married	28885 (81.0)	1441 (81.6)		28454 (79.3)	966 (85.2)	
**Owns mobile phone**						
No	5243 (14.7)	268 (15.2)	0.75	5430 (15.1)	43 (3.8)	**<0.01**
Yes	27972 (78.4)	1382 (78.3)		30470 (84.9)	1091 (96.2)	
Data not available	2448 (6.9)	115 (6.5)				

*Concrete - concrete walls and roof; Mixed - concrete walls and tiled roof; Government donated/funded house - prebuilt government houses or houses funded through schemes for economically and socially marginalised groups; Thatched - thatched walls and roof.
†Pearson's chi-squared test

When their characteristics were compared, heads of households participating in the phone-based survey were most commonly aged between 41–50 years (29.2%) in contrast to those in the non-sampled households who were mainly older than 60 years (27.7%, p<0.01). Similarly, households with female heads were under-represented in the phone-based survey (15.0%) compared to non-sampled households (18.7%, p<0.01). Households where the heads reported no formal education were also underrepresented in the phone-based survey (23.8%) compared to non-sampled households (30.6%, p<0.01). Households headed by individuals who were never

For peer review only - http://bmjopen.bmj.com/site/about/guidelines.xhtml

married were also significantly underrepresented in the phone-based survey (14.8%) compared to non-sampled households (20.7%, p<0.01). Reported ownership of mobile phones was higher (96.2%) compared to non-sampled households (84.9%, p<0.01) in the phone-based coverage evaluation survey. **(Table 1).**

Factors associated with household non-response

Of the 1983 households with complete data selected for the phone-based survey, 849 (42.8%) were considered non-responders. The selected modified Poisson regression model included SES and the education of the head of the household. Non-response increased with greater poverty, and households in the poorest quintile were more than twice as likely to have not responded as households in the least poor quintile (Adjusted Prevalence Ratios (aPR): 2.3, 95% CI:2.0–2.7), adjusting for site and education of the household head. Non-response was lower in households with heads who reported any education compared to those who reported no education (p<0.01) in the phone-based survey **(Table 2)**, adjusting for site and SES**.** Of the 1976 households with complete data and selected for the in-person survey, 211 (10.7%) were non-responders. Similar to the phone-based survey, the unadjusted estimate for non-response in the in-person survey was lower in the Jawadhu Hills sub-site compared to the Timiri sub-site (aPR:0.5, 95% CI:0.3–0.8), and age was selected (p<0.01) once sub-site was included **(Table 3).**

Table 2: Factors associated with non-response in a phone-based coverage evaluation survey in Vellore, India, 2020

	Sampled households*†	Non response‡	Univariate		Multivariable	
	N=1983 (100) n (%)	n=849 (42.8) n (%)	PR (95% CI)	P value	aPR (95% CI)	P value

For peer review only - http://bmjopen.bmj.com/site/about/guidelines.xhtml

Site details						
**Proportion of households in each arm**						
Control	993 (50.1)	429 (43.2)	REF	0.78		
Intervention	990 (49.9)	420 (42.4)	1.0 (0.9, 1.1)			
**Proportion of households per site**						
Timiri	1586 (80.0)	694 (43.8)	REF	0.45	REF	**<0.01**
Jawadhu Hills	397 (20.0)	155 (39.0)	0.9 (0.7, 1.2)		0.6 (0.4, 0.8)	
Household characteristics						
**Religion**						
Other	68 (3.4)	27 (39.7)	REF	0.67		
Hindu	1915 (96.6)	822 (42.9)	1.1 (0.8, 1.5)			
**Caste**						
Higher caste	20 (1.0)	6 (30.0)	REF	0.50		
Backward caste	635 (32.0)	271 (42.7)	1.4 (0.8, 2.6)			
Most backward caste	487 (24.6)	213 (43.7)	1.5 (0.8, 2.7)			
Scheduled caste	439 (22.1)	198 (45.1)	1.5 (0.9, 2.6)			
Scheduled tribes	402 (20.3)	161 (40.0)	1.3 (0.7, 2.5)			
**House type§**						
Concrete	1054 (53.2)	417 (39.6)	REF	**0.01**		
Mixed	233 (11.7)	113 (48.5)	1.2 (1.1, 1.4)			
Government funded/ donated house	200 (10.1)	95 (47.5)	1.2 (1.0, 1.5)			
Thatched	496 (25.0)	224 (45.2)	1.1 (1.0, 1.3)			
**Socio-economic quintiles**						
Least poor quintile	422 (21.3)	110 (26.1)	REF	**<0.01**	REF	**<0.01**
4th quintile	390 (19.7)	161 (41.3)	1.6 (1.3, 1.9)		1.5 (1.2, 1.8)	
3th quintile	406 (20.5)	185 (45.6)	1.7 (1.4, 2.1)		1.6 (1.4, 2.0)	
2nd quintile	386 (19.5)	191 (49.5)	1.9 (1.5, 2.3)		1.9 (1.6, 2.3)	
Poorest quintile	379 (19.1)	202 (53.3)	2.0 (1.7, 2.5)		2.3 (2.0, 2.7)	
**Large household (family size)**						
No (<= 4 members)	1286 (64.9)	603 (46.9)	REF	**<0.01**		
Yes (>= 5 members)	697 (35.1)	246 (35.3)	0.8 (0.7, 0.8)			
Head of the household characteristics						
**Age**						

18-30 years	84 (4.2)	31 (36.9)	REF	**<0.01**		
31-40 years	388 (19.6)	142 (36.6)	1.0 (0.8, 1.3)			
41-50 years	516 (26.0)	185 (35.9)	1.0 (0.8, 1.2)			
51-60 years	431 (21.7)	184 (42.7)	1.2 (0.9, 1.5)			
>61 years	564 (28.4)	307 (54.4)	1.5 (1.1, 2.0)			
**Sex**						
Male	1602 (80.8)	638 (39.8)	REF	**<0.01**		
Female	381 (19.2)	211 (55.4)	1.4 (1.2, 1.6)			
**Education level**						
No education	587 (29.6)	317 (54.0)	REF	**<0.01**	REF	**<0.01**
Any primary	452 (22.8)	196 (43.4)	0.8 (0.7, 0.9)		0.8 (0.7, 0.9)	
Any middle	343 (17.3)	141 (41.1)	0.8 (0.6, 0.9)		0.8 (0.6, 0.9)	
Any secondary or higher	601 (30.3)	195 (32.4)	0.6 (0.5, 0.7)		0.7 (0.6, 0.8)	
**Marriage status**						
Never married	397 (20.0)	229 (57.7)	REF	**<0.01**		
Ever married	1586 (80.0)	620 (39.1)	0.7 (0.6, 0.8)			

Acronyms: Adjusted prevalence ratio- adjusted for site; PR - Prevalence ratio; CI - confidence interval

*Excludes missing covariate data.

†Number (N) of 'sampled households' used as the denominator to estimate the prevalence of non-response in each corresponding row.

‡Non-response - no household members available for interview, household refused to participate or unable to consent, or the household was not attempted.

§Concrete - concrete walls and roof; Mixed - concrete walls and tiled roof; Government donated/funded house - prebuilt government houses or houses funded through schemes for economically and socially marginalised groups; Thatched - thatched walls and roof.

For peer review only

Table 3: Factors associated with non-response in an in-person coverage evaluation survey in Vellore, India, 2019

	In-person coverage survey					
	**Sampled households*† N=1976 (100) n (%)**	**Non response‡ n=211 (10.7) n (%)**	**Univariate PR (95% CI)**	**P value**	**Multivariable aPR (95% CI)**	**P value**
Site details						
**Proportion of households in each arm**						
Control	990 (50.1)	110 (11.1)	REF	0.65		
Intervention	986 (49.9)	101 (10.2)	0.9 (0.7, 1.3)			
**Proportion of households per site**						
Timiri	1581 (80.0)	187 (11.8)	REF	**<0.01**	REF	**<0.01**
Jawadhu Hills	395 (20.0)	24 (6.1)	0.5 (0.3, 0.8)		0.5 (0.3, 0.8)	
Household characteristics						
**Religion**						
Other	58 (2.9)	13 (22.4)	REF	**<0.01**		
Hindu	1918 (97.1)	198 (10.3)	0.5 (0.3, 0.7)			
**Caste**						
Higher caste	18 (0.9)	1 (5.6)	REF	0.18		
Backward caste	609 (30.8)	73 (12.0)	2.2 (0.3, 16.0)			
Most backward caste	502 (25.4)	62 (12.4)	2.2 (0.3, 16.2)			
Scheduled caste	439 (22.2)	46 (10.5)	1.9 (0.2, 14.4)			
Scheduled tribes	408 (20.6)	29 (7.1)	1.3 (0.2, 9.8)			
**House type§**						
Concrete	1019 (51.6)	123 (12.1)	REF	**0.02**		
Mixed	229 (11.6)	26 (11.4)	0.9 (0.6, 1.4)			
Government funded/ donated house	191 (9.7)	24 (12.6)	1.0 (0.7, 1.5)			
Thatched	537 (27.2)	38 (7.1)	0.6 (0.4, 0.9)			
**Socio-economic quintiles**						
Least poor quintile	391 (19.8)	48 (12.3)	REF	**0.02**		
4th quintile	360 (18.2)	39 (10.8)	0.9 (0.6, 1.3)			
3th quintile	429 (21.7)	54 (12.6)	1.0 (0.7, 1.6)			
2nd quintile	410 (20.7)	47 (11.5)	0.9 (0.6, 1.5)			
Poorest quintile	386 (19.5)	23 (6.0)	0.5 (0.3, 0.8)			
**Large household (family size)**						
No ≤ 4 members)	1339 (67.8)	159 (11.9)	REF	**0.02**		
Yes (≥ 5 members)	637 (32.2)	52 (8.2)	0.7 (0.5, 0.9)			
Head of the household characteristics						

For peer review only

**Age**						
18-30 years	108 (5.5)	16 (14.8)	REF	**0.03**	REF	**<0.01**
31-40 years	403 (20.4)	55 (13.6)	0.9 (0.6, 1.5)		0.8 (0.5, 1.3)	
41-50 years	489 (24.7)	38 (7.8)	0.5 (0.3, 0.9)		0.4 (0.3, 0.7)	
51-60 years	455 (23.0)	53 (11.6)	0.8 (0.5, 1.3)		0.6 (0.4, 1.0)	
>61 years	521 (26.4)	49 (9.4)	0.6 (0.3, 1.2)		0.5 (0.3, 0.9)	
**Sex**						
Male	1567 (79.3)	155 (9.9)	REF	**0.02**		
Female	409 (20.7)	56 (13.7)	1.4 (1.1, 1.8)			
**Education level**						
No education	655 (33.1)	67 (10.2)	REF	0.36		
Any primary	427 (21.6)	48 (11.2)	1.1 (0.8, 1.5)			
Any middle	360 (18.2)	31 (8.6)	0.8 (0.5, 1.4)			
Any secondary or higher	534 (27.0)	65 (12.2)	1.2 (0.9, 1.6)			
**Marriage status**						
Never married	377 (19.1)	53 (14.1)	REF	**0.01**		
Ever married	1599 (80.9)	158 (9.9)	0.7 (0.5, 0.9)			

Acronyms: aPR – Adjusted prevalence ratio- adjusted for site; PR - Prevalence ratio; CI - confidence interval

*Excludes missing covariate data.
†Number (N) of 'sampled households' used as the denominator to estimate prevalence of non-response in each corresponding row.
‡Non-response - no household members available for interview, household refused to participate or unable to consent, or the household was not attempted.
§Concrete - concrete walls and roof; Mixed - concrete walls and tiled roof; Government donated/funded house - prebuilt government houses or houses funded through schemes for economically and socially marginalised groups; Thatched - thatched walls and roof.

As a sensitivity analysis, we examined mobile phone ownership and the availability of phone numbers as additional candidate predictors. The final selected model for non-response in the phone-based survey included mobile phone ownership and SES, but it no longer included head of household education. Including mobile phone ownership attenuated but did not entirely remove the association of SES with non-response (households from the poorest SES quintile: aPR: 1.7, 95% CI:1.4–2.1). Mobile phone ownership was strongly associated with non-response in the phone-based survey independent of SES and adjusting for subsite, but not in the in-person survey, for which the results did not change with the inclusion of mobile phone ownership as a candidate predictor (**Supplementary Tables 1 & 2**). Using phone number availability

For peer review only

in place of mobile phone ownership, the predictor was again selected and had a slightly stronger association with non-response in the phone-based survey model, but there was no change in results for the in-person survey (**Supplementary Tables 3 & 4**). Cross-tabulation of phone number availability among those who reported owning a mobile phone showed that 93.5% (1461/1563) of the phone numbers were available in the in-person survey and decreased slightly to 89.6% (1524/1700) in the phone-based survey **(Supplementary Table 5).**

In 2020, 5728 (15.1%) out of the 37,883 censused households reported not owning a mobile phone. Households in the poorest quintile were 19 times more likely not to own a mobile phone than households in the least poor quintile (aPR:19.3, 95% CI:15.3–24.3). Non-ownership of phones was less likely in households with heads who were more educated compared to those who reported no education (p<0.01). (**Supplementary Table 6).**

Differences in the implementation of in-person and phone-based surveys

During the in-person survey, 2932 attempts were made to reach the 2000 households; 2376 (81.0%) respondents were located and available to participate. In the phone-based survey, 3262 attempts were made to reach the 2000 households, during which 1166 (35.7%) primary respondents were located and available to participate **(Table 4).** The phone-based surveys were most often conducted mid-day between 12:01 to 14:00 and after 18:00 hrs, in contrast to the in-person interviews conducted mainly between 10:01 to 18:00 hrs (p<0.01). The mean duration of each in-person survey visit was longer (3.5 min) than the mean time taken to complete a call during the phone-based survey (1.9 min, p<0.01). During the in-person survey, each visit

For peer review only - http://bmjopen.bmj.com/site/about/guidelines.xhtml

interviewed a median of 2 individuals per household compared to each call during the phone-based survey, when a median of 0 was interviewed per call (which includes all the calls where nobody was present/could be reached) (p<0.01).

Table 4: Comparison of implementation of in-person and phone-based coverage surveys in Vellore, India, 2019-2020

	In-person coverage survey	Phone-based coverage survey	P value*
Discrete household visits or calls	n=2932 (100.0)	n=3262 (100.0)	
**Status**			
Not located	2 (0.1)	1 (0.0)	
Members deceased	7 (0.2)	0 (0.0)	
Members not present	547 (18.7)	2095 (64.2)	**<0.01**
Members present	2376 (81.0)	1166 (35.7)	
**Time of day**			
<10:00	149 (5.1)	83 (2.5)	**<0.01**
10:01-12:00	656 (22.4)	398 (12.2)	
12:01-14:00	832 (28.4)	785 (24.1)	
14:01-16:00	659 (22.5)	535 (16.4)	
16:01-18:00	542 (18.5)	457 (14.0)	
>18:00	94 (3.2)	1004 (30.8)	
**Mean duration per visit or call (min)**	3.5 (1.8 - 6.9)	1.9 (1.0 - 4.4)	**<0.01**
**Individuals completed per visit or call**	2.0 (1.0 - 4.0)	0.0 (0.0 - 3.0)	**<0.01**
**Median residents completed per visit or call (IQR)**	66.7 (25.0 - 100.0)†	0.0 (0.0 - 100.0)†	**<0.01**
Household survey completion	n=1765 (100.0)	n=1134 (100.0)	
**Total duration to complete household survey (min)**	6.3 (3.6 - 10.6)	6.5 (3.8 - 10.8)	0.47
**Weighted mean duration (min) ‡**	9.3 (9.0 - 9.7)	8.8 (8.4 - 9.2)	**0.05**
**Visits or calls to complete household survey**			
1	981 (55.6)	780 (68.8)	**<0.01**
2	730 (41.4)	174 (15.3)	
3	51 (2.9)	61 (5.4)	
4+	3 (0.2)	119 (10.5)	

*Pearson's chi-squared/T-test as appropriate. For status analysis, not located and members deceased were excluded due to small cell counts.
†Median (Interquartile range)
‡Weighted according to the household size

For peer review only

Most in-person survey households were completed in 1 or 2 visits (97.0%), but by phone, households mainly were completed in the first attempt (68.8%). Considering the household size, the weighted mean duration was slightly longer during the in-person survey (9.3 min) than in the phone-based survey (8.8 min, $p<0.01$) **(Table 4).**

DISCUSSION

For peer review only

In this study, we compared the representativeness and implementation of a phone-based survey with an in-person survey to measure treatment coverage following cMDA for STH as part of the ongoing DeWorm3 trial in rural communities in southern India. Household participation in surveys assessing the coverage of the STH program was higher when conducted in person than when done using a phone-based survey (89.0% vs 57.2%). When comparing the characteristics of participating households, larger households, households with higher socioeconomic status and those with younger, more educated, and male heads of household were over-represented in the phone-based survey. Adjusting for sub-site, non-response in the phone-based survey was higher among households from the poorest SES quintile and households with uneducated household heads, while non-response in the in-person survey was associated with the age of the household head.

The overrepresentation of households with higher socioeconomic status in the phone-based survey is likely due to higher mobile phone ownership in that strata, as demonstrated in our findings and other developing countries [27,28]. Furthermore, the phone-based survey was implemented during the COVID-19-related lockdown in India when income and employment were drastically reduced, increasing the challenges

faced by poorer households to recharge phone subscriptions [29][30]. This difficulty could also explain lower overall participation rates in the phone-based survey. Larger households were overrepresented in the phone-based survey as the probability of someone in the family owning a phone may be higher in such households. Households with younger, more educated, and male heads of household were overrepresented in the phone-based survey. The effect of age on participation in phone-based surveys has been well documented in related health surveys from the United States, Ireland, UK and Brazil [8,12,13]. Studies from Germany and the UK also reported an under-representation of socially disadvantaged and/or low-income participants [8,10]. Low phone ownership among females residing in South Asia has been reported previously due to traditional gender norms, technical difficulties in operating a phone, male dominance over phone usage, and economic reasons [31].

Eighty per cent of households for the phone-based survey provided a mobile phone number during the annual census. However, the availability of a phone number does not always translate into a response, often due to poor network coverage, unrenewed subscriptions, or changed subscriptions, as reported in a study on cell phone ownership in Burkina Faso [32]. Nearly twice the number of households from the starting sample of 2000 households participated in the in-person survey compared to the phone-based survey. While calculating the sample size for phone-based surveys in rural communities, it is essential to oversample to adjust for high non-response rates. In the in-person survey, most participants responded during the day, corresponding to the interviewer's visiting hours. However, during implementation, we observed two distinct periods (mid-day between 12:01 to 14:00 and after 18:00 hrs) of response in the phone-based survey, perhaps because participants were engaged in

For peer review only

agricultural/outdoor activities with phones unattended, except during lunchtime and in the evenings when they were free to attend calls. While interviewers initially contacted participants on the phone during the same working hours as the in-person survey, we later adapted the time of the calls to this observed pattern. Phone calls can occur in unexpected instances, and the owner may not be in a suitable environment to respond promptly, such as during working hours, which can impact response rates [33]. While conducting phone-based surveys, we recommend contacting participants in the evenings when they are free to respond to calls. Although phone-based surveys allowed us to adapt and flexible interview timings, the local culture and work patterns should be considered to ensure high response rates.

The in-person survey took marginally longer to complete as it necessitated the interviewer to verify and interview all available household members individually. This observed difference in duration may increase if the travel time to the house for an in-person survey, particularly in hard-to-reach rural areas, is considered. The convenience offered by a phone-based study may also explain the increased number of attempts to establish contact in our phone-based survey. Arguably, the phone-based survey was faster to complete since one participant could respond on behalf of those absent, explaining higher completion rates on the first attempt. There is also a possibility of a bias introduced due to proxy responses. However, the number of attempts to establish a successful contact was more in the phone-based survey because phone calls often failed to connect, similar to phone-based surveys conducted in Ireland and the U.K [8]. There is also a possibility that proxy responses can introduce biased treatment estimates.

For peer review only - http://bmjopen.bmj.com/site/about/guidelines.xhtml

NTDs are more prevalent in socially and economically disadvantaged groups living in poor environmental conditions [34]. In this population, STH infections, particularly hookworm, are more prevalent in households belonging to poorer SES quintiles with less-educated household heads [22]. MDA programs are one of the key public health interventions implemented to control or eliminate NTDs, including STH, and a coverage survey following an MDA program evaluates the performance of the intervention. Therefore, it is imperative that the population included in a coverage evaluation survey does not exclude the same groups at a higher risk of infection to avoid biasing estimates of treatment coverage. In this regard, the phone-based survey clearly under-represents many of these groups. Our findings suggest that in-person surveys better represent the target population in rural communities than phone-based surveys.

The exhaustive and up-to-date census allowed us to robustly assess the representativeness of both survey strategies. This analysis also offered an advantage over others based in LMICs since it was conducted in a censused population in a rural setting with regular community engagement and used the same questionnaire in both surveys within a reasonable time frame, thus allowing us to compare participation confidently [35–37]. This analysis also has some limitations. The phone-based survey was conducted in a state with high teledensity and fair network coverage, even in rural areas. As a result, these findings may not be generalisable to all rural settings. In addition, this survey was conducted during the COVID-19 pandemic and during a time of lockdowns in the study region, which may have negatively impacted responsiveness to the phone survey.

For peer review only - http://bmjopen.bmj.com/site/about/guidelines.xhtml

CONCLUSIONS

In-person surveys appear to more reliably and equitably obtain representative coverage data for MDA programs, as phone-based surveys underrepresent groups at a higher risk of acquiring STH and other NTDs. As a result, phone surveys may bias coverage estimates and could potentially negatively impact the groups that most need treatment. Phone-based surveys remain a convenient alternative, especially when circumstances preclude household visits and resources are limited. If the information on population characteristics is available beforehand, researchers could consider quota sampling, where participants are selected non-randomly according to a fixed quota/percentage of the population based on one or more characteristics or post-stratification weight adjustment after phone-based surveys to ensure that all age groups and socio-economic strata are equally represented [10,38]. Both methods can be employed if non-response remains high following quota sampling. However, in the absence of accurate, up-to-date data on population characteristics, in-person surveys appear more representative.

For peer review only

Ethics statements

Patient consent for publication: Not required.

Ethics approval: The DeWorm3 study was approved by the Institutional Review Board of Christian Medical College, Vellore, India (10392, dated 30.11.2016), Human Subjects Division at the University of Washington (STUDY00000180) and The London School of Hygiene & Tropical Medicine (12013) and conformed to the principles embodied in the Declaration of Helsinki. For the in-person survey, written informed consent was sought from the head of the household or any adult member (≥18 years). For the phone-based surveys, the standardised information sheet was read out to the household head or the next eligible adult household member at the start of the call, and verbal consent over the phone was obtained. The change in protocol and current analysis was submitted to the Institutional Review Board of Christian Medical College, Vellore, as an amendment (IRB-A9-24.11.2021, dated 02.12.2022) and approved by The London School of Hygiene & Tropical Medicine (26333). The trial was registered at ClinicalTrials.gov (NCT03014167)

For peer review only

Acknowledgements

We thank field managers Rajeshkumar Rajendiran (Timiri) and Chinnaduraipandi (Jawadhu hills), Jasmine Farzana, Gokila Palaniswamy, Janarthanan Maniyarasu, Naveen Kumar Sekar and Dhanalakshmi Manoharan (at CMC, Vellore) and David S Kennedy (at LSHTM), for their meticulous work in developing and implementing the coverage data collection forms. We are most grateful to our field supervisors and field workers in Timiri and Jawadhu for exemplary work in completing the surveys despite

the pandemic limitations. We acknowledge the contributions and efforts of the entire DeWorm3 Trials Team, particularly Dr Arianna Means, Dr Kristjana Asbjornsdottir, and Emily Pearman at the University of Washington and Dr Tim Littlewood and Leanne Doran at the Natural History Museum. We are thankful for support extended to this study from the Ministry of Health and Family Welfare, Delhi and the Directorate of Public Health, Chennai, as well as help in implementing study activities from officials at the district and block levels, village leaders and members of our community advisory boards in Timiri and Jawadhu hills. Most importantly, we thank all the participants for their time.

FOOTNOTES

Contributors: RMR and WEO conceptualised the study, conducted the data analysis, and wrote the first draft with support from SSRA and KEH. JLW, the Deworm3 study PI, obtained funding for the study and helped write the paper. KA, SPK and GJI conceptualised the study, oversaw the analysis, and provided feedback on the draft. SG and HL provided access to the data and supported critical interpretation of the findings. All authors (RMR, WEO, GJI, KA, SG, HL, SPK, JLW, KEH and SSRA) provided feedback on the manuscript and approved the final version.

Funding: The DeWorm3 study is funded through a grant to the University of Washington, Seattle, Washington, United States of America, from the Bill and Melinda Gates Foundation (OPP1129535, PI JLW). Under the grant conditions of the Foundation, a Creative Commons Attribution 4.0 Generic License has already been assigned to the Author Accepted Manuscript version that might arise from this submission. The funders were not involved in the decision to publish the manuscript and had no role in data collection, analysis or publication of study results.

For peer review only - http://bmjopen.bmj.com/site/about/guidelines.xhtml

Competing interests: None declared

Provenance and peer review: Not commissioned; externally peer-reviewed.

Supplemental material: This content has been supplied by the author(s). It has not been vetted by BMJ Publishing Group Limited (BMJ) and may not have been peer-reviewed. Any opinions or recommendations discussed are solely those of the author(s) and are not endorsed by BMJ. BMJ disclaims all liability and responsibility arising from any reliance on the content. Where the content includes any translated material, BMJ does not warrant the accuracy and reliability of the translations (including but not limited to local regulations, clinical guidelines, terminology, drug names and drug dosages) and is not responsible for any error or omissions arising from translation and adaptation or otherwise.

Twitter:

Rohan Michael Ramesh: @rohan_mic

William Edward Oswald: @williameoswald

Sitara S.R. Ajjampur: @ajjampur_s

ORCID iDs:

Rohan Michael Ramesh: 0000-0002-1089-6037

William Edward Oswald: 0000-0002-5287-2834

Sitara S.R. Ajjampur: 0000-0003-3419-6577

Figure Legends

Figure 1: Timeline of phone-based coverage evaluation survey in relation to other key activities

Figure 2: Household participation details of in-person and phone-based coverage survey

For peer review only

Data availability statement

Data cannot be shared publicly because the study remains blinded to outcome data.

Following unblinding of the study, data will be available upon reasonable request.

References

1 Kempf AM, Remington PL. New Challenges for Telephone Survey Research in the Twenty-First Century. *Annu Rev Public Health* 2007;**28**:113–26. doi:10.1146/annurev.publhealth.28.021406.144059

2 Centre for Disease Control. Statistical methodology of the National Immunization Survey,1994-2002. CDC, 2005. Available:https://stacks.cdc.gov/view/cdc/6573 (accessed 1 Oct 2021).

3 Remington PL, Smith MY, Williamson DF, *et al.* Design, characteristics, and usefulness of state-based behavioral risk factor surveillance: 1981-87. *Public Health Rep* 1988;**103**:366–75.

4 Coffey D, Hathi P, Khalid N, *et al.* Using phone surveys to measure mental health. Ideas For India, 2020. Available: http://www.ideasforindia.in/topics/human-development/using-phone-surveys-to-measure-mental-health.html (accessed 1 Oct 2021).

5 Link MW, Mokdad AH. Effects of survey mode on self-reports of adult alcohol consumption: a comparison of mail, web and telephone approaches. *J Stud Alcohol* 2005;**66**:239–45.doi:10.15288/jsa.2005.66.239

6 The World Bank. Mobile cellular subscriptions (per 100 people). TWB, 2020. Available: https://data.worldbank.org/indicator/IT.CEL.SETS.P2?view=map (accessed 6 Nov 2021).

7 Gibson DG, Pereira A, Farrenkopf BA, *et al.* Mobile Phone Surveys for Collecting Population-Level Estimates in Low- and Middle-Income Countries: A Literature Review. *JMIR* 2017;**19**:e7428.doi:10.2196/jmir.7428

8 Boland M, Sweeney M, Scallan E, *et al.* Emerging advantages and drawbacks of telephone surveying in public health research in Ireland and the U.K. *BMC Public Health* 2006;**6**:208.doi:10.1186/1471-2458-6-208

9 Block ES, Erskine L. Interviewing by Telephone: Specific Considerations, Opportunities, and Challenges. *Int J Qual Methods* 2012;**11**(4):428–45.doi:10.1177/160940691201100409

For peer review only - http://bmjopen.bmj.com/site/about/guidelines.xhtml

10 Szolnoki G, Hoffmann D. Online, face-to-face and telephone surveys—Comparing different sampling methods in wine consumer research. *Wine Economics and Policy* 2013;**2**:57–66.doi:10.1016/j.wep.2013.10.001

11 Ellis CH, Krosnick JA. Comparing Telephone and Face-to-Face Surveys in Terms of Sample Representativeness: A Meta-Analysis of Demographic Characteristics. American Nation election studies, 1999. Available: http://users.clas.ufl.edu/kenwald/pos6757/spring02/tch59.pdf (accessed 25 Aug 2020).

12 National Center for Health Statistics, Blumberg SJ, Lake JV. Wireless substitution : early release of estimates From the National Health Interview Survey, July-December 2020. National Center for Health Statistics, 2021. doi:10.15620/cdc:108678

13 Moura EC, Claro RM, Bernal R, *et al.* A feasibility study of cell phone and landline phone interviews for monitoring of risk and protection factors for chronic diseases in Brazil. *Cad Saúde Pública* 2011;**27**:277–86.doi:10.1590/S0102-311X2011000200009

14 Telecom Regulatory Authority of India. TRAI releases Telecom Subscription Data as on 31st July, 2021, TRAI 2021. Available: https://www.trai.gov.in/notifications/press-release/trai-releases-telecom-subscription-data-31st-july-2021 (accessed 1 Oct 2021).

15 International Institute for Population Sciences. National Family Health Survey (NFHS-4),India, 2015-16,IIPS 2017.Available: http://rchiips.org/nfhs/nfhs-4Reports/India.pdf (accessed 30 Apr 2020).

16 International Institute for Population Sciences. NFHS-4 Tamil Nadu State Report, IIPS 2017.Available: http://rchiips.org/nfhs/NFHS-4Reports/TamilNadu.pdf (accessed 30 Apr 2020).

17 Crompton DWT. Preventive chemotherapy in human helminthiasis : coordinated use of anthelminthic drugs in control interventions : a manual for health professionals and programme managers. World Health Organization, 2006. Available:https://apps.who.int/iris/handle/10665/43545 (accessed 8 Nov 2021).

18 World Health Organisation. Preventive chemotherapy: Tools for improving the quality of reported data and information, 2019. Available:https://apps.who.int/iris/bitstream/handle/10665/329376/9789241516464-eng.pdf

19 USAID. Post MDA Survey Design: Monitoring treatment coverage of neglected disease control programs, in A toolkit developed for USAID Neglected Tropical Disease Control Project, 2009. Available:https://pdf.usaid.gov/pdf_docs/Pdacq848.pdf

20 World Health Organization. Monitoring and epidemiological assessment of mass drug administration in the global programme to eliminate lymphatic filariasis : a manual for national elimination programmes. *Suivi et évaluation*

For peer review only

épidémiologique du traitement médicamenteux de masse dans le cadre du Programme mondial pour l'élimination de la filariose lymphatique : manuel à l'intention des programmes nationauxd'élimination Published Online First: 2011. Available: https://apps.who.int/iris/handle/10665/44580 (accessed 15 Nov 2021).

21 Evidence Action. World's Largest Deworming Program in India To Start With Support from Evidence Action, 2015. Available:https://www.evidenceaction.org/largest-deworming-program-in-india-to-start-with-support-from-evidence-action/ (accessed 31 Jan 2022).

22 Ajjampur SSR, Kaliappan SP, Halliday KE, *et al.* Epidemiology of soil transmitted helminths and risk analysis of hookworm infections in the community: Results from the DeWorm3 Trial in southern India. *PLoS Negl Trop Dis* 2021;**15**:e0009338.doi:10.1371/journal.pntd.0009338

23 Ásbjörnsdóttir KH, Ajjampur SSR, Anderson RM, *et al.* Assessing the feasibility of interrupting the transmission of soil-transmitted helminths through mass drug administration: The DeWorm3 cluster randomized trial protocol. *PLoS Negl Trop Dis* 2018;**12**:e0006166.doi:10.1371/journal.pntd.0006166

24 DeWorm3 Trials Team. Baseline patterns of infection in regions of Benin, Malawi and India seeking to interrupt transmission of soil transmitted helminths (STH) in the DeWorm3 trial. *PLoS Negl Trop Dis* 2020;**14**:e0008771.doi:10.1371/journal.pntd.0008771

25 National Health Portal Of India. National Deworming Day, 2021. Available: https://www.nhp.gov.in/national-deworming-day-2021_pg (accessed 6 Apr 2022).

26 Zhang Z. Variable selection with stepwise and best subset approaches. *Ann Transl Med* 2016;**4**.doi:10.21037/atm.2016.03.35

27 Tran MC, Labrique AB, Mehra S, *et al.* Analyzing the Mobile "Digital Divide": Changing Determinants of Household Phone Ownership Over Time in Rural Bangladesh. *JMIR mHealth and uHealth* 2015;**3**:e3663.doi:10.2196/mhealth.3663

28 Labrique A, Blynn E, Ahmed S, *et al.* Health Surveys Using Mobile Phones in Developing Countries: Automated Active Strata Monitoring and Other Statistical Considerations for Improving Precision and Reducing Biases. *JMIR* 2017;**19**:e7329.doi:10.2196/jmir.7329

29 Centre for Sustainable Employment. Covid-19 Employment and Income Tracker,2020. Available:https://cse.azimpremjiuniversity.edu.in/covid19-data-tracker/ (accessed 1 Dec 2021).

30 Kumar C. TRAI: India lost 1.7 crore mobile subscribers in lockdown months. The Times of India, 2020. Available: https://timesofindia.indiatimes.com/india/trai-india-lost-1-7-crore-mobile-subscribers-in-lockdown-months/articleshow/79273419.cms

For peer review only

31 Giorgia Barboni, Erica Field, Rohini Pande, *et al.* A Tough Call: Understanding barriers to and impacts of women's mobile phone adoption in India. Harvard Kennedy School, 2018. Available: https://research.hks.harvard.edu/publications/getFile.aspx?Id=2765

32 Greenleaf AR, Ahmed S, Moreau C, *et al.* Cell phone ownership and modern contraceptive use in Burkina Faso: implications for research and interventions using mobile technology. *Contraception* 2019;**99**:170. doi:10.1016/j.contraception.2018.11.006

33 Dal Grande E, Chittleborough CR, Campostrini S, *et al.* Bias of health estimates obtained from chronic disease and risk factor surveillance systems using telephone population surveys in Australia: results from a representative face-to-face survey in Australia from 2010 to 2013. *BMC Med Res Methodol* 2016;**16**:44.doi:10.1186/s12874-016-0145-z

34 8 Aagaard-Hansen J, Chaignat CL. Neglected tropical diseases: equity and social determinants. World Health Organization 2010;**8**:135–157. Available:http://citeseerx.ist.psu.edu/viewdoc/download?doi=10.1.1.394.6922&rep=rep1&type=pdf

35 Nagpal K, Mathur MR, Biswas A, *et al*. Who do phone surveys miss, and how to reduce exclusion: recommendations from phone surveys in nine Indian states. *BMJ Glob Health* 2021;**6**:e005610.

36 Nambiar D, Benny G Usaidali. Telephony and trade-offs in fieldwork with the 'unreached': on the conduct of telephonic interviews with indigenous study participants in southern India. *BMJ Glob Health* 2021;**6:**e006261

37 Mohan D, Scott K, Shah N*, et al.* Can health information through mobile phones close the divide in health behaviours among the marginalised? An equity analysis of Kilkari in Madhya Pradesh, India. *BMJ Glob Health* 2021;**6:**e005512.

35. Quota Sampling. In: *The SAGE Encyclopedia of Educational Research, Measurement, and Evaluation*. 2455 Teller Road, Thousand Oaks, California 91320:: SAGE Publications, Inc. 2018.doi:10.4135/9781506326139.n565

For peer review only

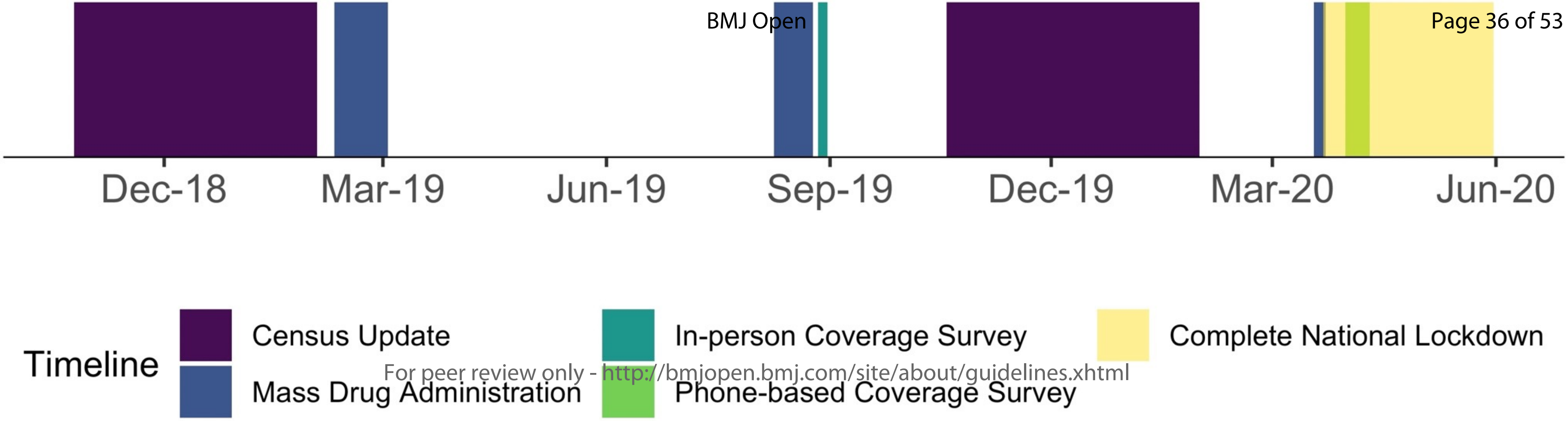

For peer review only - http://bmjopen.bmj.com/site/about/guidelines.xhtml

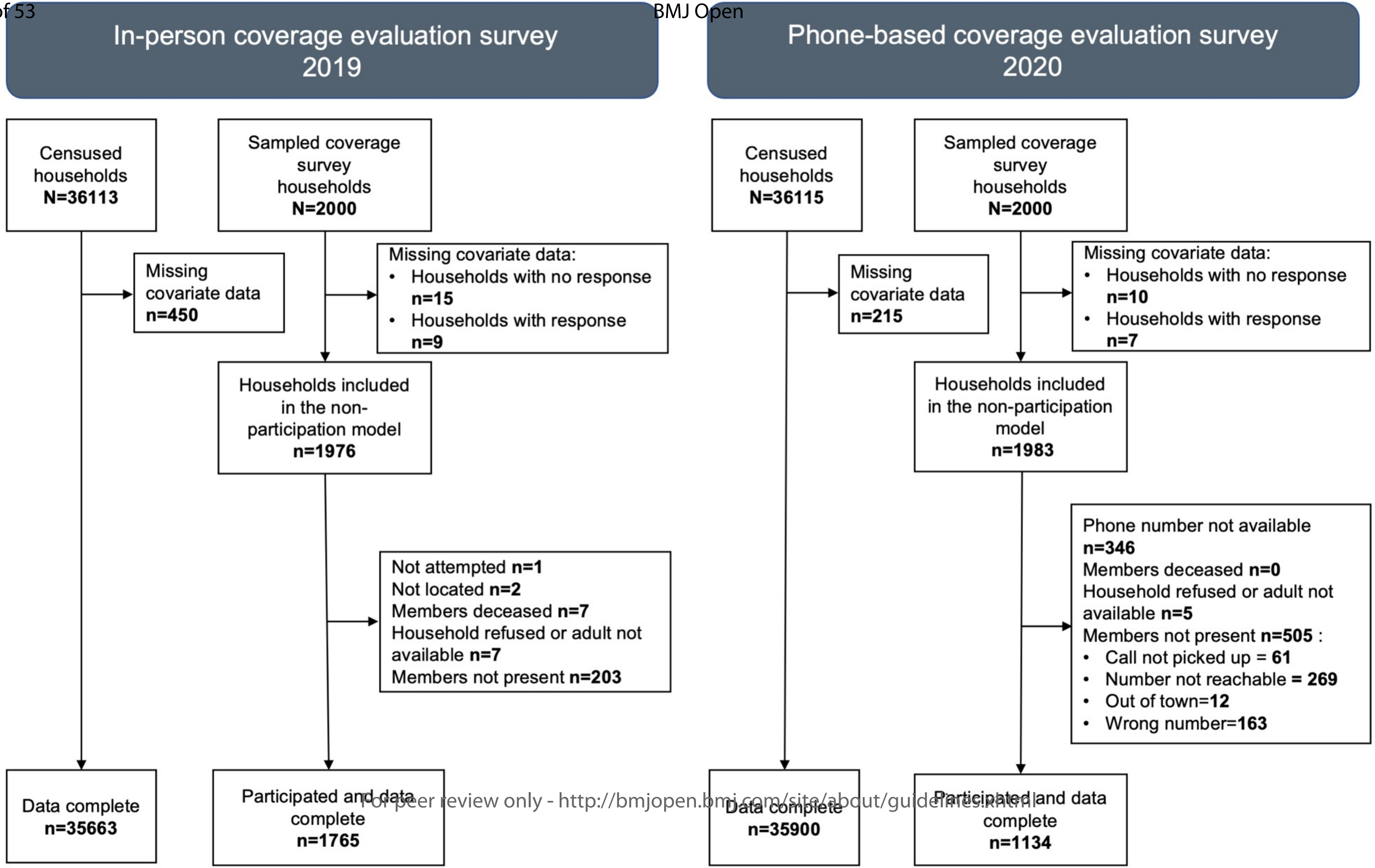

For peer review only - http://bmjopen.bmj.com/site/about/guidelines.xhtml

Supplementary Table 1 - Factors associated with non-response in a phone-based coverage evaluation survey, including mobile phone ownership

	Phone-based coverage survey					
	Sampled households*†	Non response‡	Univariate		Multivariable	
	N=1983 (100) n (%)	n=849 (42.8) n (%)	PR (95% CI)	P value	aPR (95% CI)	P value
Site details						
**Proportion of households in each arm**						
Control	993 (50.1)	429 (43.2)	REF	0.78		
Intervention	990 (49.9)	420 (42.4)	1.0 (0.9, 1.1)			
**Proportion of households per site**						
Timiri	1586 (80.0)	694 (43.8)	REF	0.45	REF	**0.04**
Jawadhu Hills	397 (20.0)	155 (39.0)	0.9 (0.7, 1.2)		0.7 (0.6, 1.0)	
Household characteristics						
**Religion**						
Other	68 (3.4)	27 (39.7)	REF	0.67		
Hindu	1915 (96.6)	822 (42.9)	1.1 (0.8, 1.5)			
**Caste**						
Higher caste	20 (1.0)	6 (30.0)	REF	0.50		
Backward caste	635 (32.0)	271 (42.7)	1.4 (0.8, 2.6)			
Most backward caste	487 (24.6)	213 (43.7)	1.5 (0.8, 2.7)			
Scheduled caste	439 (22.1)	198 (45.1)	1.5 (0.9, 2.6)			
Scheduled tribes	402 (20.3)	161 (40.0)	1.3 (0.7, 2.5)			
**House type§**						
Concrete	1054 (53.2)	417 (39.6)	REF	**0.01**		
Mixed	233 (11.7)	113 (48.5)	1.2 (1.1, 1.4)			
Government funded/ donated house	200 (10.1)	95 (47.5)	1.2 (1.0, 1.5)			
Thatched	496 (25.0)	224 (45.2)	1.1 (1.0, 1.3)			
**Socio-economic quintiles**						
Least poorest quintile	422 (21.3)	110 (26.1)	REF	**<0.01**	REF	**<0.01**
4th quintile	390 (19.7)	161 (41.3)	1.6 (1.3, 1.9)		1.5 (1.2, 1.8)	
3th quintile	406 (20.5)	185 (45.6)	1.7 (1.4, 2.1)		1.5 (1.3, 1.9)	
2th quintile	386 (19.5)	191 (49.5)	1.9 (1.5, 2.3)		1.6 (1.4, 1.9)	
Poorest quintile	379 (19.1)	202 (53.3)	2.0 (1.7, 2.5)		1.7 (1.4, 2.1)	
**Large household (family size)**						
No (<= 4 members)	1286 (64.9)	603 (46.9)	REF	**<0.01**		

For peer review only

Yes (>= 5 members)	697 (35.1)	246 (35.3)	0.8 (0.7, 0.8)			
**Owns mobile phone**						
No	298 (15.0)	255 (85.6)	REF	**<0.01**	REF	**<0.01**
Yes	1685 (85.0)	594 (35.3)	0.4 (0.4, 0.5)		0.5 (0.4, 0.5)	
Head of the household characteristics						
**Age**						
18-30 years	84 (4.2)	31 (36.9)	REF	**<0.01**		
31-40 years	388 (19.6)	142 (36.6)	1.0 (0.8, 1.3)			
41-50 years	516 (26.0)	185 (35.9)	1.0 (0.8, 1.2)			
51-60 years	431 (21.7)	184 (42.7)	1.2 (0.9, 1.5)			
>61 years	564 (28.4)	307 (54.4)	1.5 (1.1, 2.0)			
**Sex**						
Male	1602 (80.8)	638 (39.8)	REF	<0.01		
Female	381 (19.2)	211 (55.4)	1.4 (1.2, 1.6)			
**Education level**						
No education	587 (29.6)	317 (54.0)	REF	**<0.01**		
Any primary	452 (22.8)	196 (43.4)	0.8 (0.7, 0.9)			
Any middle	343 (17.3)	141 (41.1)	0.8 (0.6, 0.9)			
Any secondary or higher	601 (30.3)	195 (32.4)	0.6 (0.5, 0.7)			
**Marriage status**						
Never married	397 (20.0)	229 (57.7)	REF	**<0.01**		
Ever married	1586 (80.0)	620 (39.1)	0.7 (0.6, 0.8)			

Acronyms: aPR – Adjusted prevalence ratio- adjusted for site; PR - Prevalence ratio; CI - confidence interval

* Excludes missing covariate data. For status analysis : not located and members deceased excluded due to small cell counts

†Number (N) of 'sampled households' used as the denominator to estimate prevalence of non-response in each corresponding row.

‡Non-response - no household members available for interview, household refused to participate or unable to consent, or the household was not attempted.

§Concrete - concrete walls and roof; Mixed - concrete walls and tiled roof; Government donated/funded house - prebuilt government houses or houses funded through schemes for economically and socially marginalized groups; Thatched - thatched walls and roof.

For peer review only

Supplementary Table 2 - Factors associated with non-response in an in-person coverage evaluation survey, including mobile phone ownership

	In-person coverage survey					
	**Sampled households*†**	**Non response‡**	**Univariate**		**Multivariable**	
	**N=1976 (100) n (%)**	**n=211 (10.7) n (%)**	**PR (95% CI)**	**P value**	**aPR (95% CI)**	**P value**
Site details						
**Proportion of households in each arm**						
Control	990 (50.1)	110 (11.1)	REF	0.65		
Intervention	986 (49.9)	101 (10.2)	0.9 (0.7, 1.3)			
**Proportion of households per site**						
Timiri	1581 (80.0)	187 (11.8)	REF	**<0.01**	REF	**<0.01**
Jawadhu Hills	395 (20.0)	24 (6.1)	0.5 (0.3, 0.8)		0.5 (0.3, 0.8)	
Household characteristics						
**Religion**						
Other	58 (2.9)	13 (22.4)	REF	**<0.01**		
Hindu	1918 (97.1)	198 (10.3)	0.5 (0.3, 0.7)			
**Caste**						
Higher caste	18 (0.9)	1 (5.6)	REF	0.18		
Backward caste	609 (30.8)	73 (12.0)	2.2 (0.3, 16.0)			
Most backward caste	502 (25.4)	62 (12.4)	2.2 (0.3, 16.2)			
Scheduled caste	439 (22.2)	46 (10.5)	1.9 (0.2, 14.4)			
Scheduled tribes	408 (20.6)	29 (7.1)	1.3 (0.2, 9.8)			
**House type§**						
Concrete	1019 (51.6)	123 (12.1)	REF	0.02		
Mixed	229 (11.6)	26 (11.4)	0.9 (0.6, 1.4)			
Government funded/ donated house	191 (9.7)	24 (12.6)	1.0 (0.7, 1.5)			
Thatched	537 (27.2)	38 (7.1)	0.6 (0.4, 0.9)			
**Socio-economic quintiles**						
Least poorest quintile	391 (19.8)	48 (12.3)	REF	**0.02**		
4th quintile	360 (18.2)	39 (10.8)	0.9 (0.6, 1.3)			
3th quintile	429 (21.7)	54 (12.6)	1.0 (0.7, 1.6)			
2th quintile	410 (20.7)	47 (11.5)	0.9 (0.6, 1.5)			
Poorest quintile	386 (19.5)	23 (6.0)	0.5 (0.3, 0.8)			
**Large household (family size)**						

For peer review only

No (<= 4 members)	1339 (67.8)	159 (11.9)	REF	**0.02**		
Yes (>= 5 members)	637 (32.2)	52 (8.2)	0.7 (0.5, 0.9)			
**Owns mobile phone**						
No	293 (14.8)	25 (8.5)	REF	0.16		
Yes	167 (10.8)	1.3 (0.9, 1.8)	1.3 (0.9, 1.8)			
Data not available	134 (6.8)	19 (14.2)	1.7 (1.0, 2.8)			
Head of the household characteristics						
**Age**						
18-30 years	108 (5.5)	16 (14.8)	REF	**0.03**	REF	**<0.01**
31-40 years	403 (20.4)	55 (13.6)	0.9 (0.6, 1.5)		0.8 (0.5, 1.3)	
41-50 years	489 (24.7)	38 (7.8)	0.5 (0.3, 0.9)		0.4 (0.3, 0.7)	
51-60 years	455 (23.0)	53 (11.6)	0.8 (0.5, 1.3)		0.6 (0.4, 1.0)	
>61 years	521 (26.4)	49 (9.4)	0.6 (0.3, 1.2)		0.5 (0.3, 0.9)	
**Sex**						
Male	1567 (79.3)	155 (9.9)	REF	**0.02**		
Female	409 (20.7)	56 (13.7)	1.4 (1.1, 1.8)			
**Education level**						
No education	655 (33.1)	67 (10.2)	REF	0.36		
Any primary	427 (21.6)	48 (11.2)	1.1 (0.8, 1.5)			
Any middle	360 (18.2)	31 (8.6)	0.8 (0.5, 1.4)			
Any secondary or higher	534 (27.0)	65 (12.2)	1.2 (0.9, 1.6)			
**Marriage status**						
Never married	377 (19.1)	53 (14.1)	REF	**0.01**		
Ever married	1599 (80.9)	158 (9.9)	0.7 (0.5, 0.9)			

Acronyms: Adjusted prevalence ratio- adjusted for site; PR - Prevalence ratio; CI - confidence interval

*Excludes missing covariate data.
†Number (N) of 'sampled households' used as the denominator to estimate prevalence of non-response in each corresponding row.
‡Non-response - no household members available for interview, household refused to participate or unable to consent, or the household was not attempted.
§Concrete - concrete walls and roof; Mixed - concrete walls and tiled roof; Government donated/funded house - prebuilt government houses or houses funded through schemes for economically and socially marginalized groups; Thatched - thatched walls and roof.

For peer review only

Supplementary Table 3 - Factors associated with non-response in a phone-based coverage survey, including phone number availability

	Phone-based coverage survey					
	**Sampled households*†**	**Non response‡**	**Univariate**		**Multivariable**	
	**N=1983 (100) n (%)**	**n=849 (42.8) n (%)**	**PR (95% CI)**	**P value**	**aPR (95% CI)**	**P value**
Site details						
**Proportion of households in each arm**						
Control	993 (50.1)	429 (43.2)	REF	0.78		
Intervention	990 (49.9)	420 (42.4)	1.0 (0.9, 1.1)			
**Proportion of households per site**						
Timiri	1586 (80.0)	694 (43.8)	REF	0.45	REF	**0.08**
Jawadhu Hills	397 (20.0)	155 (39.0)	0.9 (0.7, 1.2)		0.8 (0.6, 1.0)	
Household characteristics						
**Religion**						
Other	68 (3.4)	27 (39.7)	REF	0.67		
Hindu	1915 (96.6)	822 (42.9)	1.1 (0.8, 1.5)			
**Caste**						
Higher caste	20 (1.0)	6 (30.0)	REF	0.50		
Backward caste	635 (32.0)	271 (42.7)	1.4 (0.8, 2.6)			
Most backward caste	487 (24.6)	213 (43.7)	1.5 (0.8, 2.7)			
Scheduled caste	439 (22.1)	198 (45.1)	1.5 (0.9, 2.6)			
Scheduled tribes	402 (20.3)	161 (40.0)	1.3 (0.7, 2.5)			
**House type§**						
Concrete	1054 (53.2)	417 (39.6)	REF	**0.01**		
Mixed	233 (11.7)	113 (48.5)	1.2 (1.1, 1.4)			
Government funded/ donated house	200 (10.1)	95 (47.5)	1.2 (1.0, 1.5)			
Thatched	496 (25.0)	224 (45.2)	1.1 (1.0, 1.3)			
**Socio-economic quintiles**						
Least poorest quintile	422 (21.3)	110 (26.1)	REF	**<0.01**	REF	**<0.01**
4th quintile	390 (19.7)	161 (41.3)	1.6 (1.3, 1.9)		1.5 (1.3, 1.9)	
3th quintile	406 (20.5)	185 (45.6)	1.7 (1.4, 2.1)		1.5 (1.2, 1.7)	
2th quintile	386 (19.5)	191 (49.5)	1.9 (1.5, 2.3)		1.6 (1.4, 1.9)	
Poorest quintile	379 (19.1)	202 (53.3)	2.0 (1.7, 2.5)		1.7 (1.4, 2.0)	
**Large household (family size)**						
No (<= 4 members)	1286 (64.9)	603 (46.9)	REF	**<0.01**		

Yes (>= 5 members)	697 (35.1)	246 (35.3)	0.8 (0.7, 0.8)			
**Phone number available**						
No	405 (20.4)	382 (94.3)	REF	**<0.01**	REF	**<0.01**
Yes	1578 (79.6)	467 (29.6)	0.3 (0.3, 0.4)		0.3 (0.3, 0.4)	
Head of the household characteristics						
**Age**						
18-30 years	84 (4.2)	31 (36.9)	REF	**<0.01**		
31-40 years	388 (19.6)	142 (36.6)	1.0 (0.8, 1.3)			
41-50 years	516 (26.0)	185 (35.9)	1.0 (0.8, 1.2)			
51-60 years	431 (21.7)	184 (42.7)	1.2 (0.9, 1.5)			
>61 years	564 (28.4)	307 (54.4)	1.5 (1.1, 2.0)			
**Sex**						
Male	1602 (80.8)	638 (39.8)	REF	**<0.01**		
Female	381 (19.2)	211 (55.4)	1.4 (1.2, 1.6)			
**Education level**						
No education	587 (29.6)	317 (54.0)	REF	**<0.01**		
Any primary	452 (22.8)	196 (43.4)	0.8 (0.7, 0.9)			
Any middle	343 (17.3)	141 (41.1)	0.8 (0.6, 0.9)			
Any secondary or higher	601 (30.3)	195 (32.4)	0.6 (0.5, 0.7)			
**Marriage status**						
Never married	397 (20.0)	229 (57.7)	REF	**<0.01**		
Ever married	1586 (80.0)	620 (39.1)	0.7 (0.6, 0.8)			

Acronyms: aPR – Adjusted prevalence ratio- adjusted for site; PR - Prevalence ratio; CI - confidence interval

*Excludes missing covariate data.
†Number (N) of 'sampled households' used as the denominator to estimate prevalence of non-response in each corresponding row.
‡Non-response - no household members available for interview, household refused to participate or unable to consent, or the household was not attempted.
§Concrete - concrete walls and roof; Mixed - concrete walls and tiled roof; Government donated/funded house - prebuilt government houses or houses funded through schemes for economically and socially marginalized groups; Thatched - thatched walls and roof.

For peer review only

Supplementary Table 4 - Factors associated with non-response in an in-person coverage survey, including phone number availability

	In-person coverage survey					
	**Sampled households*†**	**Non response‡**	**Univariate**		**Multivariable**	
	**N=1976 (100) n (%)**	**n=211 (10.7) n (%)**	**PR (95% CI)**	**P value**	**aPR (95% CI)**	**P value**
Site details						
**Proportion of households in each arm**						
Control	990 (50.1)	110 (11.1)	REF	0.65		
Intervention	986 (49.9)	101 (10.2)	0.9 (0.7, 1.3)			
**Proportion of households per site**						
Timiri	1581 (80.0)	187 (11.8)	REF	**<0.01**	REF	**<0.01**
Jawadhu Hills	395 (20.0)	24 (6.1)	0.5 (0.3, 0.8)		0.5 (0.3, 0.8)	
Household characteristics						
**Religion**						
Other	58 (2.9)	13 (22.4)	REF	**<0.01**		
Hindu	1918 (97.1)	198 (10.3)	0.5 (0.3, 0.7)			
**Caste**						
Higher caste	18 (0.9)	1 (5.6)	REF	0.18		
Backward caste	609 (30.8)	73 (12.0)	2.2 (0.3, 16.0)			
Most backward caste	502 (25.4)	62 (12.4)	2.2 (0.3, 16.2)			
Scheduled caste	439 (22.2)	46 (10.5)	1.9 (0.2, 14.4)			
Scheduled tribes	408 (20.6)	29 (7.1)	1.3 (0.2, 9.8)			
**House type§**						
Concrete	1019 (51.6)	123 (12.1)	REF	0.02		
Mixed	229 (11.6)	26 (11.4)	0.9 (0.6, 1.4)			
Government funded/ donated house	191 (9.7)	24 (12.6)	1.0 (0.7, 1.5)			
Thatched	537 (27.2)	38 (7.1)	0.6 (0.4, 0.9)			
**Socio-economic quintiles**						
Least poor quintile	391 (19.8)	48 (12.3)	REF	**0.02**		
4th quintile	360 (18.2)	39 (10.8)	0.9 (0.6, 1.3)			
3th quintile	429 (21.7)	54 (12.6)	1.0 (0.7, 1.6)			
2th quintile	410 (20.7)	47 (11.5)	0.9 (0.6, 1.5)			
Poorest quintile	386 (19.5)	23 (6.0)	0.5 (0.3, 0.8)			
**Large household (family size)**						
No (<= 4 members)	1339 (67.8)	159 (11.9)	REF	**0.02**		

For peer review only

Yes (>= 5 members)	637 (32.2)	52 (8.2)	0.7 (0.5, 0.9)			
**Phone number available**						
No	273 (13.8)	26 (9.5)	REF	**<0.01**		
Yes	1703 (86.2)	185 (10.9)	1.1 (0.8, 1.6)			
Head of the household characteristics						
**Age**						
18-30 years	108 (5.5)	16 (14.8)	REF	**0.03**	REF	**<0.01**
31-40 years	403 (20.4)	55 (13.6)	0.9 (0.6, 1.5)		0.8 (0.5, 1.3)	
41-50 years	489 (24.7)	38 (7.8)	0.5 (0.3, 0.9)		0.4 (0.3, 0.7)	
51-60 years	455 (23.0)	53 (11.6)	0.8 (0.5, 1.3)		0.6 (0.4, 1.0)	
>61 years	521 (26.4)	49 (9.4)	0.6 (0.3, 1.2)		0.5 (0.3, 0.9)	
**Sex**						
Male	1567 (79.3)	155 (9.9)	REF	**0.02**		
Female	409 (20.7)	56 (13.7)	1.4 (1.1, 1.8)			
**Education level**						
No education	655 (33.1)	67 (10.2)	REF	0.36		
Any primary	427 (21.6)	48 (11.2)	1.1 (0.8, 1.5)			
Any middle	360 (18.2)	31 (8.6)	0.8 (0.5, 1.4)			
Any secondary or higher	534 (27.0)	65 (12.2)	1.2 (0.9, 1.6)			
**Marriage status**						
Never married	377 (19.1)	53 (14.1)	REF	**0.01**		
Ever married	1599 (80.9)	158 (9.9)	0.7 (0.5, 0.9)			

Acronyms: Adjusted prevalence ratio- adjusted for site; PR - Prevalence ratio; CI - confidence interval

*Excludes missing covariate data.
†Number (N) of 'sampled households' used as the denominator to estimate prevalence of non-response in each corresponding row.
‡Non-response - no household members available for interview, household refused to participate or unable to consent, or the household was not attempted.
§Concrete - concrete walls and roof; Mixed - concrete walls and tiled roof; Government donated/funded house - prebuilt government houses or houses funded through schemes for economically and socially marginalized groups; Thatched - thatched walls and roof.

For peer review only

Supplementary Table 5 - Phone number availability among mobile phone ownership reported in the annual census

Owns mobile phone	In-person coverage survey households (n=1563)	Phone-based coverage survey households (n=1700)	P value
Household phone number available			
**No**	102 (6.5)	176 (10.4)	**<0.01**
**Yes**	1461 (93.5)	1524 (89.6)	

For peer review only

Supplementary Table 6 - Factors associated with reported non-ownership of a mobile phone in Tamil Nadu, 2019

	Censused households 2019-2020 N=37883 (100) n (%)	Non ownership N=5728 (15.1) n (%)	Univariate PR (95% CI)	P value	Multivariable aPR (95% CI)	P value
Site details						
**Proportion of households in each arm**						
Control	18424 (48.6)	2800 (15.2)	REF	0.91		
Intervention	19459 (51.4)	2928 (15.0)	1.0 (0.8, 1.2)			
**Proportion of households per site**						
Timiri	29545 (78.0)	4159 (14.1)	REF	**<0.01**	REF	**<0.01**
Jawadhu Hills	8338 (22.0)	1569 (18.8)	1.3 (1.1, 1.7)		0.4 (0.3, 0.4)	
Household characteristics						
**Religion**						
Other	1133 (3.0)	93 (8.2)	REF	**<0.01**		
Hindu	36750 (97.0)	5635 (15.3)	1.9 (1.5, 2.4)			
**Caste**						
Higher	430 (1.1)	50 (11.6)	REF	**<0.01**		
Backward	11362 (30.0)	1406 (12.4)	1.1 (0.8, 1.5)			
Most backward	9207 (24.3)	1334 (14.5)	1.2 (0.9, 1.8)			
Scheduled	8457 (22.3)	1264 (14.9)	1.3 (0.9, 1.8)			
Scheduled tribes	8427 (22.2)	1674 (19.9)	1.7 (1.2, 2.4)			
**House type*** **						
Concrete	20055 (52.9)	1861 (9.3)	REF	**<0.01**		
Mixed	4190 (11.1)	750 (17.9)	1.9 (1.8, 2.1)			
Government funded/ donated house	3518 (9.3)	626 (17.8)	1.9 (1.7, 2.1)			
Thatched	10120 (26.7)	2491 (24.6)	2.7 (2.4, 2.9)			
**Socio-economic quintiles**						
Least poor quintile	7573 (20.0)	132 (1.7)	REF	**<0.01**	REF	**<0.01**
4th quintile	7569 (20.0)	302 (4.0)	2.3 (1.8, 2.9)		2.0 (1.6, 2.5)	
3th quintile	7540 (19.9)	876 (11.6)	6.7 (5.3, 8.4)		5.2 (4.1, 6.5)	
2th quintile	7598 (20.1)	1750 (23.0)	13.2 (9.9, 17.7)		10.9 (8.5, 14.1)	
Poorest quintile	7603 (20.1)	2668 (35.1)	20.1 (15.4, 26.3)		19.3 (15.3, 24.3)	
**Large household (family size)**						

No (<= 4 members)	24965 (65.9)	4947 (19.8)	REF	**<0.01**		
Yes (>= 5 members)	12918 (34.1)	781 (6.0)	0.3 (0.3, 0.4)			
**Head of the household characteristics**						
**Age**						
18-30 years	1600 (4.2)	144 (9.0)	REF	**<0.01**		
31-40 years	7230 (19.1)	538 (7.4)	0.8 (0.7, 1.0)			
41-50 years	9708 (25.6)	783 (8.1)	0.9 (0.7, 1.1)			
51-60 years	8848 (23.4)	1160 (13.1)	1.5 (1.1, 1.9)			
>61 years	10497 (27.7)	3103 (29.6)	3.3 (2.5, 4.2)			
**Sex**						
Male	30800 (81.3)	3203 (10.4)	REF	**<0.01**		
Female	7083 (18.7)	2525 (35.6)	3.4 (3.1, 3.7)			
**Education level**						
No education	11581 (30.6)	3518 (30.4)	REF	**<0.01**	REF	**<0.01**
Any primary	8613 (22.7)	1240 (14.4)	0.5 (0.4, 0.5)		0.6 (0.6, 0.6)	
Any middle	6762 (17.8)	517 (7.6)	0.3 (0.2, 0.3)		0.4 (0.3, 0.4)	
Any secondary or higher	10927 (28.8)	453 (4.1)	0.1 (0.1, 0.2)		0.3 (0.3, 0.3)	
**Marriage status**						
Never married	7843 (20.7)	2750 (35.1)	REF	**<0.01**		
Ever married†	30040 (79.3)	2978 (9.9)	0.3 (0.3, 0.3)			

Acronyms: Adjusted prevalence ratio- adjusted for site; PR - Prevalence ratio; CI - confidence interval

* Number (N) of 'sampled households' used as the denominator to estimate prevalence of non-ownership in each corresponding row.
*** Concrete - concrete walls and roof; Mixed - concrete walls and tiled roof; Government donated/funded house - prebuilt government houses or houses funded through schemes for economically and socially marginalized groups; Thatched - thatched walls and roof

For peer review only

Coverage Evaluation Survey Questionnaire

1	Name of the participant		
2	Sex if the participant		
3	Age of the participant in years		
4.1	Can the participant answer the questions now?	1	Yes, agrees to participate
		2	Not present
		3	No, refused
		4	No, other
		5	Proxy respondent
4.2	Why has household refused to participate?	1	Refused to speak/Not interested
		2	Prior negative experience
		3	Unwilling to provide detailed household information
		4	Unwilling because of concerns related to risks, confidentiality, etc.
		5	Unwilling to sign/provide thumb impression
		6.	Other
4.3	If unavailable, where is the participant?	1	At school
		2	At college
		3	At work
		4	Not at home (e.g at shop)
		5	Moved away permanently
		6	Temporarily migrated
		7	Died
		8.	Other
5	Name of the proxy respondent		
6	Sex of the proxy respondent		
7	Age of the proxy respondent		
8	Did someone give you a tablet in the past month to treat worms (at home, at school, or in the community)?	1	Yes
		2	No
		3.	Don't know/Cannot recall
		4.	Refused
8.1	If 'No', Why didn't you receive the tablet?	1	Unaware of MDA
		2	Distributor did not come to my community
		3	Distributor came to my community but did not come to my house
		4	Distributor did not come to my school
		5	The distributor was out of tablets and did not return
		6	Pregnant
		7	Breastfeeding
		8	Ill
		9	Told I could not take a tablet that day for other reasons
		10	Too old
		11	Underage (less than 1yo)
		12	I was busy
		13	I was too far away
		14	Other
		15	Don't know/Cannot recall
		16	Refused
8.2	If 'Yes', Did you swallow the tablet?	1	Yes
		2	No
		3	Don't know/Cannot recall
		4	Refused
8.3	Why did you not swallow the tablet?	1	Pregnant
		2	Breastfeeding
		3	Ill
		4	Too old
		5	Underage (less than 1yo)
		6	Afraid of side effects
		7	I am not at risk for this disease
		8	I am taking other medications
		9	Medicine doesn't work
		10	Other
		11	Don't know/Cannot recall
		12	Refused
8.4	Why did you swallow the tablet?	1	To be healthy
		2	Afraid of getting the disease
		3	Someone told me to
		4	Everyone else is taking it
		5	Don't know/Cannot recall

For peer review only

		6	Refused
		7	Other
9	Did you swallow the tablet in front of the person who gave it to you?	1	Yes
		2	No
		3	Don't know/Cannot recall
		4	Refused
10	Who gave you the tablet?	1	Family member
		2	Health staff
		3	Field worker
		4	Drug distributor
		5	Community leader
		6	Teacher /Anganwadi worker
		7	Other
		8	Don’t know/Cannot recall
		9	Refused
11	Where did they give you the tablet?	1	At home
		2	At school
		3	At Anganwadi centre
		4	Elsewhere in community
		5	Other
		6	Don’t know/Cannot recall
		7	Refused
12	Did you feel any side effects after swallowing the tablet?	1	Yes
		2	No
		3	Don't know/Cannot recall
		4	Refused
13.1	Which of the following statements are true about side effects you experienced after swallowing the tablet?	1	Mild (did not interfere with daily activities)
		2	Moderate (interfered with daily activities)
		3	Serious (required hospitalization)
		4	Don’t know/Cannot recall
		5	Refused
14	How many of these tablets did you swallow?	1	1/2
		2	1
		3	2
		4	3
		5	4
		6	5+
		7	Don’t know/Cannot recall
		8	Refused
15	Were you aware that tablets would be distributed?	1	Yes
		2	No
		3	Don't know/Cannot recall
		4	Refused
16	How did you know that the tablets would be distributed?	1	Family member/friend/neighbor
		2	Professional health staff
		3	Community drug distributor/community health worker/teacher
		4	Community or religious leader
		5	Brochures/flyers
		6	Posters
		7	Banners
		8	Radio
		9	TV
		10	Social media
		11	Community Theatre/Drama
		12	Gram sabha
		13	Don't know/Cannot recall
		14	Refused to answer
		15	Other
17	Did any of your neighbors take the distributed tablet?	1	Yes
		2	No
		3	Don't know/Cannot recall
		4	Refused
18	What did you like about the community treatment programme?	1	House to house treatment makes it easy
		2	Trusted distributors
		3	No long wait for drugs
		4	Free drugs
		5	No specific part I liked
		6	Don't know/Cannot recall
		7	Refused to answer

For peer review only

		8	Other
19	What did you not like about the community treatment programme?	1<br>2<br>3<br>4<br>5<br>6<br>7<br>8<br>9<br>10	Inconvenient time<br>Drugs ran out or were not available<br>Unfriendly distributor<br>Took too much time<br>Adverse reaction to drug<br>Did not also give treatment for other diseases<br>No specific part I disliked<br>Don't know/Cannot recall<br>Refused to answer<br>Other
20	Next time the tablets are given, how would you want them distributed?	1<br>2<br>3<br>4<br>5<br>6	The same door-to-door<br>All individuals go to school<br>Distributors in central places in the village<br>Don't know/Cannot recall<br>Refused to answer<br>Other
21	Did you have to change your daily routine to participate in the treatment day?	1<br>2<br>3<br>4	Yes<br>No<br>Don't know/Cannot recall<br>Refused
22	About how much of your time did it take to participate in the treatment day, including the time you spent waiting for the drug distributor to arrive?	1<br>2<br>3<br>4<br>5<br>6<br>7<br>8	Less than one hour<br>About one hour<br>Half of a day<br>A full day<br>More than one day, distributor did not come on day planned<br>Don’t know/Cannot recall<br>Refused to say<br>Other
23	About how many minutes did the drug distributor spend at your house on the treatment day?	1<br>2<br>3<br>4<br>5<br>6<br>7<br>8<br>9<br>10<br>11	Less than 5 minutes<br>About 10 minutes<br>About 20 minutes<br>About 30 minutes<br>About 45 minutes<br>About one hour<br>More than one hour<br>Drugs were left here when we were not home<br>Don't know/Cannot recall<br>Refused to answer<br>Other
24	If there are no adult members available, where are they?	1<br>2<br>3	Not at home (e.g., at shop)<br>Moved away permanently<br>Temporarily migrated

For peer review only

STROBE Statement—Checklist of items that should be included in reports of ***cross-sectional studies***

	Item No	Recommendation	Page No
**Title and abstract**	1	*(a)* Indicate the study's design with a commonly used term in the title or the abstract **DONE – in the Abstract**	3
		(b) Provide in the abstract an informative and balanced summary of what was done and what was found **DONE – in the Abstract**	3
**Introduction**			
Background/rationale	2	Explain the scientific background and rationale for the investigation being reported **DONE – See Introduction section**	5-6
Objectives	3	State specific objectives, including any prespecified hypotheses **DONE – See Introduction section**	6
**Methods**			
Study design	4	Present key elements of study design early in the paper **DONE – See Methods section**	7-9
Setting	5	Describe the setting, locations, and relevant dates, including periods of recruitment, exposure, follow-up, and data collection **DONE – See Methods section**	7-9
Participants	6	*(a)* Give the eligibility criteria, and the sources and methods of selection of participants **DONE – See Methods section**	7-9
Variables	7	Clearly define all outcomes, exposures, predictors, potential confounders, and effect modifiers. Give diagnostic criteria, if applicable **DONE – See Methods section**	8-11
Data sources/ measurement	8*	For each variable of interest, give sources of data and details of methods of assessment (measurement). Describe comparability of assessment methods if there is more than one group **DONE – See Methods section**	*10-11*
Bias	9	Describe any efforts to address potential sources of bias **N/A**	
Study size	10	Explain how the study size was arrived at **DONE – See Methods section**	8-11
Quantitative variables	11	Explain how quantitative variables were handled in the analyses. If applicable, describe which groupings were chosen and why **DONE – See Methods section**	10-11
Statistical methods	12	*(a)* Describe all statistical methods, including those used to control for confounding **DONE – See Methods section**	10-11
		(b) Describe any methods used to examine subgroups and interactions **DONE – See Methods section**	10-11
		(c) Explain how missing data were addressed **N/A**	-

For peer review only - http://bmjopen.bmj.com/site/about/guidelines.xhtml

		(d) If applicable, describe analytical methods taking account of sampling strategy<br>**N/A**	-
		(e) Describe any sensitivity analyses<br>**DONE – See Methods and Analysis section**	11
**Results**			
Participants	13*	(a) Report numbers of individuals at each stage of study—eg numbers potentially eligible, examined for eligibility, confirmed eligible, included in the study, completing follow-up, and analysed<br>**DONE – See Results section/Figure 2**	12-13
		(b) Give reasons for non-participation at each stage<br>**DONE – See Results section**	12-13
		(c) Consider use of a flow diagram<br>**DONE – See Results section/ Figure 2**	12-13
Descriptive data	14*	(a) Give characteristics of study participants (eg demographic, clinical, social) and information on exposures and potential confounders<br>**DONE – See Results section**	13-15
		(b) Indicate number of participants with missing data for each variable of interest<br>**DONE – See Results section/Figure 2**	12-13
Outcome data	15*	Report numbers of outcome events or summary measures<br>**DONE – See Results section**	13-23
Main results	16	*(a)* Give unadjusted estimates and, if applicable, confounder-adjusted estimates and their precision (eg, 95% confidence interval). Make clear which confounders were adjusted for and why they were included<br>**DONE – See Results section**	13-23
		(b) Report category boundaries when continuous variables were categorized<br>**DONE – See Results section**	13-23
		(c) If relevant, consider translating estimates of relative risk into absolute risk for a meaningful time period<br>**N/A**	-
Other analyses	17	Report other analyses done—eg analyses of subgroups and interactions, and sensitivity analyses<br>**DONE – See Results section**	20-21
**Discussion**			
Key results	18	Summarise key results with reference to study objectives<br>**DONE – see Discussion section**	23-26
Limitations	19	Discuss limitations of the study, taking into account sources of potential bias or imprecision. Discuss both direction and magnitude of any potential bias<br>**DONE – see Discussion section**	26-27

For peer review only

Interpretation	20	Give a cautious overall interpretation of results considering objectives, limitations, multiplicity of analyses, results from similar studies, and other relevant evidence<br>**DONE – see Discussion section**	23-26
Generalisability	21	Discuss the generalisability (external validity) of the study results<br>**DONE – see Discussion section**	23-27
**Other information**			
Funding	22	Give the source of funding and the role of the funders for the present study and, if applicable, for the original study on which the present article is based<br>**DONE – see Funding section**	29

*Give information separately for exposed and unexposed groups.

Note: An Explanation and Elaboration article discusses each checklist item and gives methodological background and published examples of transparent reporting. The STROBE checklist is best used in conjunction with this article (freely available on the Web sites of PLoS Medicine at http://www.plosmedicine.org/, Annals of Internal Medicine at http://www.annals.org/, and Epidemiology at http://www.epidem.com/). Information on the STROBE Initiative is available at www.strobe-statement.org.

For peer review only

BMJ Open

Representativeness of a mobile phone-based coverage evaluation survey following mass drug administration for soil-transmitted helminths: a comparison of participation between two cross-sectional surveys

Journal:	*BMJ Open*
Manuscript ID	bmjopen-2022-070077.R1
Article Type:	Original research
Date Submitted by the Author:	07-Aug-2023
Complete List of Authors:	Ramesh, Rohan; Christian Medical College Vellore, The Wellcome Trust Research Laboratory, Division of Gastrointestinal Sciences<br>Oswald, William; London School of Hygiene & Tropical Medicine, Department of Disease Control<br>Israel, Gideon ; Christian Medical College Vellore, The Wellcome Trust Research Laboratory, Division of Gastrointestinal Sciences<br>Aruldas, Kumudha; Christian Medical College Vellore, The Wellcome Trust Research Laboratory, Division of Gastrointestinal Sciences<br>Galagan, Sean ; University of Washington, Department of Global Health<br>Legge, Hugo; London School of Hygiene & Tropical Medicine, Department of Disease Control<br>PUTHUPALAYAM KALIAPPAN, SARAVANAKUMAR; Christian Medical College Vellore, The Wellcome Trust Research Laboratory, Division of Gastrointestinal Sciences<br>Walson, Judd; University of Washington, Departments of Global Health, Medicine, Pediatrics and Epidemiology<br>Halliday, Katherine; London School of Hygiene and Tropical Medicine Faculty of Infectious and Tropical Diseases, Department of Disease Control<br>Ajjampur, Sitara; Christian Medical College Vellore, The Wellcome Trust Research Laboratory, Division of Gastrointestinal Sciences
**Primary Subject Heading**:	Public health
Secondary Subject Heading:	Research methods, Qualitative research, Global health
Keywords:	Public health < INFECTIOUS DISEASES, Tropical medicine < INFECTIOUS DISEASES, QUALITATIVE RESEARCH

SCHOLARONE™
Manuscripts

I, the Submitting Author has the right to grant and does grant on behalf of all authors of the Work (as defined in the below author licence), an exclusive licence and/or a non-exclusive licence for contributions from authors who are: i) UK Crown employees; ii) where BMJ has agreed a CC-BY licence shall apply, and/or iii) in accordance with the terms applicable for US Federal Government officers or employees acting as part of their official duties; on a worldwide, perpetual, irrevocable, royalty-free basis to BMJ Publishing Group Ltd ("BMJ") its licensees and where the relevant Journal is co-owned by BMJ to the co-owners of the Journal, to publish the Work in this journal and any other BMJ products and to exploit all rights, as set out in our licence.

The Submitting Author accepts and understands that any supply made under these terms is made by BMJ to the Submitting Author unless you are acting as an employee on behalf of your employer or a postgraduate student of an affiliated institution which is paying any applicable article publishing charge ("APC") for Open Access articles. Where the Submitting Author wishes to make the Work available on an Open Access basis (and intends to pay the relevant APC), the terms of reuse of such Open Access shall be governed by a Creative Commons licence – details of these licences and which Creative Commons licence will apply to this Work are set out in our licence referred to above.

Other than as permitted in any relevant BMJ Author's Self Archiving Policies, I confirm this Work has not been accepted for publication elsewhere, is not being considered for publication elsewhere and does not duplicate material already published. I confirm all authors consent to publication of this Work and authorise the granting of this licence.

For peer review only

BMJ Open (Original article)

Title page

Representativeness of a mobile phone-based coverage evaluation survey following mass drug administration for soil-transmitted helminths: a comparison of participation between two cross-sectional surveys

Corresponding author

Sitara S.R. Ajjampur: The Wellcome Trust Research Laboratory, Division of Gastrointestinal Sciences, Christian Medical College, Vellore, Tamil Nadu, India-632002. Email: sitararao@cmcvellore.ac.in; Ph: +914162282052

Authors

Rohan Michael Ramesh*: The Wellcome Trust Research Laboratory, Division of Gastrointestinal Sciences, Christian Medical College, Vellore, Tamil Nadu, India

William Edward Oswald*: Department of Disease Control, Faculty of Infectious and Tropical Diseases, London School of Hygiene & Tropical Medicine, London, United Kingdom; Global Health Division, International Development Group, RTI International, Research Triangle Park, NC, USA.

Gideon John Israel: The Wellcome Trust Research Laboratory, Division of Gastrointestinal Sciences, Christian Medical College, Vellore, Tamil Nadu, India

Kumudha Aruldas: The Wellcome Trust Research Laboratory, Division of Gastrointestinal Sciences, Christian Medical College, Vellore, Tamil Nadu, India

For peer review only

Sean Galagan: Department of Global Health, University of Washington, Seattle, Washington, United States of America

Hugo Legge: Department of Disease Control, Faculty of Infectious and Tropical Diseases, London School of Hygiene & Tropical Medicine, London, United Kingdom

Saravanakumar Puthupalayam Kaliappan: The Wellcome Trust Research Laboratory, Division of Gastrointestinal Sciences, Christian Medical College, Vellore, Tamil Nadu, India

Judd L Walson: Departments of Global Health, Medicine, Pediatrics and Epidemiology, University of Washington, Seattle, Washington, United States of America

Katherine Elizabeth Halliday: Department of Disease Control, Faculty of Infectious and Tropical Diseases, London School of Hygiene & Tropical Medicine, London, United Kingdom

Sitara S.R. Ajjampur: The Wellcome Trust Research Laboratory, Division of Gastrointestinal Sciences, Christian Medical College, Vellore, Tamil Nadu, India

* These authors contributed equally to the work.

For peer review only

Keywords

Phone-based survey, coverage evaluation survey, in-person survey, low-and middle-income countries, representativeness, non-response, participation, India

Word Count: 4049

ABSTRACT

Objectives: With increasing mobile phone subscriptions, phone-based surveys are gaining popularity with public health programs. Despite advantages, systematic exclusion of participants may limit representativeness. Similar to control programs for neglected tropical diseases, the DeWorm3 trial of biannual community-wide mass drug administration (MDA) for elimination of soil-transmitted helminth (STH) infection used in-person coverage evaluation surveys to measure the proportion of the at-risk population treated during MDA. Due to lockdown during the COVID-19 pandemic, a phone-based coverage evaluation survey was necessary, providing an opportunity for the current study to compare representativeness and implementation (including non-response) of these two survey modes.

Design: Comparison of two cross-sectional surveys

Setting: The DeWorm3 trial site in Tamil Nadu, India, includes Timiri, a rural sub-site, and Jawadhu hills, a hilly, hard-to-reach sub-site inhabited predominantly by a tribal population.

Participants: In the phone-based and in-person coverage evaluation surveys, all individuals residing in 2000 randomly selected households (50 in each of the 40 trial clusters) were eligible to participate. Here we characterise household participation.

Results: Of 2000 households, 1780 (89.0%) participated during the in-person survey. Of 2000 households selected for the phone survey, 346 (17.3%) could not be contacted as they had not provided a telephone number during the census and 1144 (57.2%) participated. Smaller households, households with lower socioeconomic status, and those with older, female or less educated household-heads were under-represented in the phone-based survey compared to censused households. Regression analysis revealed non-response in the phone-based survey was higher

For peer review only - http://bmjopen.bmj.com/site/about/guidelines.xhtml

among households from the poorest socioeconomic quintile (Prevalence Ratio (PR): 2.3,95% Confidence Interval (CI):2.0–2.7) and lower when heads of households had completed secondary school or higher education (PR: 0.7,95% CI:0.6–0.8).

Conclusions: Our findings suggest phone-based surveys underrepresent households likely to be at higher risk of NTDs and in-person surveys are more appropriate for measuring MDA coverage within programmatic settings.

Trial registration number: NCT03014167 (ClinicalTrials.gov)

For peer review only

Strengths and limitations

- A detailed and up-to-date census in the study population allowed us to robustly assess representativeness of both survey modes.
- Both surveys conducted six months apart used the same questionnaire which allows us to compare participation confidently.
- Generalizability may be limited as the study was conducted in a setting with high mobile phone usage and network coverage and during the COVID-19 pandemic and resulting lockdown.

INTRODUCTION

Telephone-based surveys have been widely used in public health programs and research, for purposes such as determining immunisation coverage of children under three years, conducting surveillance for risk factors related to leading causes of death, assessing mental health, and self-reporting alcohol consumption [1-5]. This survey mode has become popular with increasing mobile phone subscription rates in low-middle-income countries, where cell phone coverage in some regions compares with that in high-income countries [6,7]. Cost-effectiveness, time efficiency, increased reach, and willingness to share sensitive information are some advantages of phone-based surveys [8,9]. Disadvantages of phone-based surveys include the inability to collect visual data, lower response rates in some communities, and exclusion of participants without access to phones [9,10]. Mobile phone ownership is not universal, and the practical appeal of phone-based surveys may come at a cost by systematically excluding segments of the population, raising questions about the representativeness of the estimates they yield [8,11–13]. India's average tele-density, or the number of telephone connections (wireless and wired) for every hundred individuals living within an area, is 88.5, with a slight disparity between most rural and urban areas [14,15]. Tamil Nadu, in southern India, with a population of 72 million, is the fifth most tele-dense state of the 28 states in India with 108.5 telephone connections per 100 people, which is higher than the Indian average, with high mobile phone penetration even in rural households [14,15].

Neglected tropical diseases (NTDs) predominantly affect impoverished, rural communities, and preventive chemotherapy (PC) through mass drug administration

For peer review only

(MDA) is one of the key public health interventions implemented to control soil-transmitted helminth (STH) infections, lymphatic filariasis (LF), trachoma, schistosomiasis, and onchocerciasis [16]. The World Health Organization (WHO) refers to coverage evaluation surveys as population-based surveys that offer a simple and effective method to accurately assess programme performance and provide precise estimates of PC coverage, or the proportion of individuals who swallowed the medicine or combination of medicines, for targeted NTDs [17,18]. These surveys are a valuable tool for evaluating the performance of NTD control programs. For programs targeting lymphatic filariasis, trachoma and onchocerciasis, community-wide MDA (cMDA) coverage is assessed by household surveys using a standardised WHO tool [17,19].

DeWorm3 is a trial of cMDA for interrupting transmission of STH, during which household coverage evaluation surveys were conducted after each of six cMDA rounds in the years 2018–2020 [20,21]. In April 2020, however, during the nationwide lockdown imposed by the Government of India as a control measure during the COVID-19 pandemic, a phone-based survey was conducted instead of an in-person survey as in the previous four rounds. Leveraging this change in the trial protocol, necessitated by the COVID-19 pandemic, we conducted this analysis to contrast the representativeness, or household participation, and implementation of a phone-based survey with a previous in-person survey conducted six months earlier.

For peer review only

METHODS

DeWorm3 Trial

DeWorm3 is a cluster-randomised, controlled, community-based intervention trial that aims to determine the feasibility of interrupting STH transmission in Benin, India, and Malawi [21,22]. In brief, 40 clusters at each study site were randomised to control clusters, to receive the standard of care, which in India is twice-yearly targeted deworming of children 1 – 19 years of age during National Deworming Days (NDDs) [23], or intervention clusters, to receive twice-yearly cMDA (delivered door-to-door) for three years (between 2018 and 2020) (**Figure 1**) [24]. At the start of each of the three years, an annual census was conducted to obtain accurate population data at the individual and household level and optimise coverage estimates during cMDAs. In all intervention clusters, cMDA was implemented simultaneously the day after NDD, followed by a mop-up revisiting previously unavailable households. Following each of the six rounds of cMDA, coverage evaluation surveys were conducted in the intervention and control clusters within a week of cMDA. Surveys in control clusters were intended to measure the coverage of treatment provided through the NDD program. All census and coverage evaluation surveys (in-person and phone-based) were collected using the same electronic data collection forms programmed into the SurveyCTO mobile application (Dobility, Inc; Cambridge, Massachusetts and Washington, DC) run on encrypted Android smartphones. The survey forms in this application were embedded with logic checks, skip logic, numerical constraints and pre-loaded with location and demographic information to reduce errors while entering data.

For peer review only

Study setting

The DeWorm3 trial site in India includes a sub-site in Timiri, a rural area in Ranipet district (formerly a part of Vellore district), and another sub-site in Jawadhu hills in Tiruvannamalai district, a hilly, hard-to-reach area inhabited predominantly by tribal groups. Both sub-sites are located in the southern state of Tamil Nadu. The study covers an annually enumerated population of nearly 141,000, residing in approximately 37,000 households, with a total area of 477 km^2 [20]. Males and females are equally represented, and children below 15 years of age constitute 23% of the population. Nearly a third of the adults received no formal education, with 20% and 90% of the population engaged in agricultural activities in Timiri and Jawadhu hills, respectively [21]. More details on India's study site and population have been published previously [20].

Coverage evaluation surveys

For the coverage evaluation surveys, 50 households were randomly selected to participate from each of the 40 clusters (n=2,000). Questions in the survey aligned with the WHO-endorsed coverage evaluation survey for use by national programs, including individual-level questions on receipt and swallowing of the drug and reasons for not taking treatment, if applicable (**Supplementary file**) [18]. The in-person coverage evaluation survey in 2019 was conducted from 27th to 31st August, based on a household sampling frame derived from the previous annual census (25th October 2018 to 2nd February 2019) **(Figure 1).** All households had to be visited up to three times before they were considered unavailable, and the reasons were recorded. Sampled households that were not located or unavailable were substituted as necessary with households from two randomly-selected staged replacement lists of

For peer review only - http://bmjopen.bmj.com/site/about/guidelines.xhtml

20 households per cluster. First, the respondent answered the household-level questions, and then each member present was interviewed. Proxy responses from household members were accepted for children under five years of age and household members absent on the second or third visit.

The phone-based coverage evaluation survey was conducted from 31st March to 10th April 2020 with a household sampling frame derived from the previous annual census (19th October 2019 to 31st January 2020) (**Figure 1**). All households were eligible for selection, regardless of whether they had provided a phone number during the census or not. Households without a telephone number, who did not consent, or whose number was not reachable (two attempts made four hours apart were made on the first day and the third and fourth attempts made the next day) were also substituted with households from replacement lists. The interviewers were trained to administer the coverage evaluation survey over the phone while recording responses on the SurveyCTO mobile application on another device. The next available adult was interviewed using the same phone number if the head of the household (preferred respondent) was unavailable for three attempts. Thereafter, other household members were also interviewed over the phone during the same call. The primary respondent was allowed to provide proxy responses for children under five years and household members absent on any attempt.

Data analysis

To assess representativeness, we compared the characteristics of households that participated in each of the coverage evaluation surveys with those of households in the previous census not randomly-selected for the coverage evaluation survey and

For peer review only - http://bmjopen.bmj.com/site/about/guidelines.xhtml

identified factors associated with household non-response during both of the coverage evaluation surveys. To achieve the sample size for each coverage evaluation survey, additional lists of households were randomly selected and released to the survey teams to compensate for unavailable households. For the current study contrasting representativeness, we included only the initial 2,000 households sampled for each survey, and restricted analyses to households with complete data. We compared characteristics of households participating in each of the coverage evaluation surveys with households in the most recent previous annual census considering age, sex, reported education, and marital status of household head; household religion and caste; and period of time in residence and house type. Principal component analysis (PCA) using household assets was used to arrive at a composite wealth index and divided into five socioeconomic status (SES) quintiles, as described previously [20]. Participants who were currently married, separated, widowed or divorced were considered ever married. Chi-squared tests were used to compare distributions of household characteristics between census and surveyed households.

For each coverage evaluation survey, we identified factors associated with household non-response by fitting modified Poisson regression models with robust errors to adjust for clustering [25]. For this analysis, a household was considered to have a response if at least one household member was present and agreed to respond to the survey. Households not meeting this criterion for any reason were considered a non-response. We estimated the prevalence ratio (PR) between each candidate predictor variable and non-response. We then used a best-subset selection approach, modelling all possible combinations of candidate predictor variables hypothesised to influence a household's participation and selecting the final model with the smallest

For peer review only - http://bmjopen.bmj.com/site/about/guidelines.xhtml

Bayesian Information Criterion [26]. All candidate models included sub-site, and household caste was excluded from the multivariable model selection because of collinearity with sub-site. In a sensitivity analysis, we examined the inclusion of mobile phone ownership and phone number availability as additional candidate predictors to determine factors associated with household non-response. As a *post hoc* analysis, we also examined the association between households who reported owning a mobile phone and phone number availability.

In addition, we contrasted the implementation of each of the coverage evaluation surveys by comparing the number of attempts to contact (visits or calls), time of the day the visits/calls were made, duration per visit or call, number of individuals interviewed per attempt, proportion of household residents interviewed per attempt, total duration to complete the household's survey, and number of visits or calls to complete the household survey. We used a nonparametric K-sample test of medians for continuous measures, a Pearson's chi-squared test for categorical measures, and compared mean total duration weighted by household size using a Wald test. Statistical significance for all tests was set at p-value<0.05. The data were managed and analysed using STATA 16.1 software (StataCorp, TX, USA).

Patient and public involvement

Community members living in STH endemic areas were not involved in the design, conduct, reporting, or dissemination of this study.

RESULTS

For peer review only - http://bmjopen.bmj.com/site/about/guidelines.xhtml

Households available for participation in coverage evaluation surveys

During the 2019 in-person coverage evaluation survey, of the 2000 households sampled, 1780 (89.0%) participated, and during the 2020 phone-based coverage evaluation survey, 1144 (57.2%) of 2000 sampled households participated. Data were incomplete for 24 and 17 households of those sampled for the in-person and phone-based surveys, respectively. The number of households not participating due to various reasons during the in-person and phone-based surveys is illustrated in **Figure 2**. During the in-person survey, members were unavailable in 203 (10.1%) households even after three visits. During the phone-based survey, 346 (17.3%) households could not be contacted as phone numbers were unavailable. Further, 505 (25.2%) households were unavailable after three or more phone calls – these included 61 households where the phone call was not picked up, 269 for whom the 'number not reachable' message was received from the phone service provider as the phone was outside an area of connection, 12 primary respondents informed the interviewers that they were 'out of town' and not present during the cMDA and as a result were unable to answer on behalf of their family members, and 163 were recorded as 'wrong number' as the call was attended by persons not associated with the surveyed household.

Representativeness of coverage evaluation surveys

No differences were observed when the characteristics of households and heads of households participating in the in-person coverage evaluation survey were compared to censused households that were not sampled **(Table 1).** In the phone-based survey, however, participating households in the highest SES quintile were overrepresented (27.5%) compared to non-sampled households (19.9%), with the inverse observed in

For peer review only - http://bmjopen.bmj.com/site/about/guidelines.xhtml

the poorest quintile (p<0.01). Larger households with five or more household members were also overrepresented in the phone-based survey (39.8%) compared to non-sampled households (34.0%, p<0.01).

Table 1 - Comparison of household characteristics between censused and participating households of in-person and phone-based coverage surveys in Vellore, India, 2018-2020

	Census households (2018-19) N=35663 n(%)	In-person coverage survey N=1765 n(%)	P value†	Census households (2019-20) N=35900 n(%)	Phone-based coverage survey N=1134 n(%)	P value†
Site details						
**Study arm**						
Control	17420 (48.8)	880 (49.9)	0.41	17431 (48.6)	564 (49.7)	0.43
Intervention	18243 (51.2)	885 (50.1)		18469 (51.4)	570 (50.3)	
**Study sub-site**						
Timiri	27638 (77.5)	1394 (79.0)	0.14	27959 (77.9)	892 (78.7)	0.53
Jawadhu Hills	8025 (22.5)	371 (21.0)		7941 (22.1)	242 (21.3)	
Household characteristics						
**Religion**						
Other	1086 (3.0)	45 (2.5)	0.24	1065 (3.0)	41 (3.6)	0.21
Hindu	34577 (97.0)	1720 (97.5)		34835 (97.0)	1093 (96.4)	
**Caste**						
Higher caste	404 (1.1)	17 (1.0)	0.66	410 (1.1)	14 (1.2)	0.55
Backward caste	10667 (29.9)	536 (30.4)		10727 (29.9)	364 (32.1)	
Most backward caste	8533 (23.9)	440 (24.9)		8720 (24.3)	274 (24.2)	
Scheduled caste	7960 (22.3)	393 (22.3)		8018 (22.3)	241 (21.3)	
Scheduled tribes	8099 (22.7)	379 (21.5)		8025 (22.4)	241 (21.3)	
**House type***						
Concrete	18525 (51.9)	896 (50.8)	0.78	19001 (52.9)	637 (56.2)	0.13
Mixed	3975 (11.1)	203 (11.5)		3957 (11.0)	120 (10.6)	
Government donated/funded house	3380 (9.5)	167 (9.5)		3318 (9.2)	105 (9.3)	
Thatched	9783 (27.4)	499 (28.3)		9624 (26.8)	272 (24.0)	
**Socio-economic quintiles**						
Least poor quintile	7120 (20.0)	343 (19.4)	0.58	7151 (19.9)	312 (27.5)	**<0.01**
4th quintile	6965 (19.5)	321 (18.2)		7179 (20.0)	229 (20.2)	
3th quintile	7273 (20.4)	375 (21.2)		7134 (19.9)	221 (19.5)	

2nd quintile	7118 (20.0)	363 (20.6)		7212 (20.1)	195 (17.2)	
Poorest quintile	7187 (20.2)	363 (20.6)		7224 (20.1)	177 (15.6)	
**Large household (family size)**						
No (<= 4 members)	24027 (67.4)	1180 (66.9)	0.65	23679 (66.0)	683 (60.2)	**<0.01**
Yes (>= 5 members)	11636 (32.6)	585 (33.1)		12221 (34.0)	451 (39.8)	
Head of the household characteristics						
**Age**						
18-30 years	2143 (6.0)	92 (5.2)	0.68	1516 (4.2)	53 (4.7)	**<0.01**
31-40 years	7071 (19.8)	348 (19.7)		6842 (19.1)	246 (21.7)	
41-50 years	9103 (25.5)	451 (25.6)		9192 (25.6)	331 (29.2)	
51-60 years	7827 (21.9)	402 (22.8)		8417 (23.4)	247 (21.8)	
>61 years	9519 (26.7)	472 (26.7)		9933 (27.7)	257 (22.7)	
**Sex**						
Male	28260 (79.2)	1412 (80.0)	0.44	29198 (81.3)	964 (85.0)	**<0.01**
Female	7403 (20.8)	353 (20.0)		6702 (18.7)	170 (15.0)	
**Education level**						
No education	12047 (33.8)	588 (33.3)	0.55	10994 (30.6)	270 (23.8)	**<0.01**
Any primary	7604 (21.3)	379 (21.5)		8161 (22.7)	256 (22.6)	
Any middle	6210 (17.4)	329 (18.6)		6419 (17.9)	202 (17.8)	
Any secondary or higher	9802 (27.5)	469 (26.6)		10326 (28.8)	406 (35.8)	
**Marriage status**						
Never married	6778 (19.0)	324 (18.4)	0.50	7446 (20.7)	168 (14.8)	**<0.01**
Ever married	28885 (81.0)	1441 (81.6)		28454 (79.3)	966 (85.2)	
**Owns mobile phone**						
No	5243 (14.7)	268 (15.2)	0.75	5430 (15.1)	43 (3.8)	**<0.01**
Yes	27972 (78.4)	1382 (78.3)		30470 (84.9)	1091 (96.2)	
Data not available	2448 (6.9)	115 (6.5)				

*Concrete - concrete walls and roof; Mixed - concrete walls and tiled roof; Government donated/funded house - prebuilt government houses or houses funded through schemes for economically and socially marginalised groups; Thatched - thatched walls and roof.
†Pearson's chi-squared test

When their characteristics were compared, heads of households participating in the phone-based survey were most commonly aged between 41–50 years (29.2%) in contrast to those in the non-sampled households who were mainly older than 60 years (27.7%, p<0.01). Similarly, households with female heads were under-represented in the phone-based survey (15.0%) compared to non-sampled households (18.7%,

For peer review only

p<0.01). Households where the heads reported no formal education were also underrepresented in the phone-based survey (23.8%) compared to non-sampled households (30.6%, p<0.01). Households headed by individuals who were never married were also significantly underrepresented in the phone-based survey (14.8%) compared to non-sampled households (20.7%, p<0.01). Reported ownership of mobile phones was higher (96.2%) compared to non-sampled households (84.9%, p<0.01) in the phone-based coverage evaluation survey. **(Table 1).**

Factors associated with household participation

Of the 1983 households with complete data sampled for the phone-based survey, 849 (42.8%) were considered non-responders. The selected modified Poisson regression model included SES and the education of the head of the household. Non-response increased with greater poverty, and households in the poorest quintile were more than twice as likely to have not responded as households in the least poor quintile (PR: 2.3, 95% CI:2.0–2.7), adjusting for site and education of the household head. Non-response was lower in households with heads who reported any education compared to those who reported no education (p<0.01) in the phone-based survey **(Table 2)**, adjusting for site and SES**.** Of the 1976 households sampled for the in-person survey and with complete data, 211 (10.7%) were non-responders. Similar to the phone-based survey, the unadjusted estimate for non-response in the in-person survey was lower in the Jawadhu Hills sub-site compared to the Timiri sub-site (PR: 0.5, 95% CI:0.3–0.8), and age was selected (p<0.01) once sub-site was included **(Table 3).**

Table 2: Factors associated with non-response in a phone-based coverage evaluation survey in Vellore, India, 2020

For peer review only - http://bmjopen.bmj.com/site/about/guidelines.xhtml

	Households† N=1983 (100) n (%)	Non-response‡ n=849 (42.8) n (%)	Univariate PR (95% CI)	P value	Multivariable PR (95% CI)	P value
Site details						
**Proportion of households in each arm**						
Control	993 (50.1)	429 (43.2)	REF	0.78		
Intervention	990 (49.9)	420 (42.4)	1.0 (0.9, 1.1)			
**Proportion of households per site**						
Timiri	1586 (80.0)	694 (43.8)	REF	0.45	REF	**<0.01**
Jawadhu Hills	397 (20.0)	155 (39.0)	0.9 (0.7, 1.2)		0.6 (0.4, 0.8)	
Household characteristics						
**Religion**						
Other	68 (3.4)	27 (39.7)	REF	0.67		
Hindu	1915 (96.6)	822 (42.9)	1.1 (0.8, 1.5)			
**Caste**						
Higher caste	20 (1.0)	6 (30.0)	REF	0.50	--	--
Backward caste	635 (32.0)	271 (42.7)	1.4 (0.8, 2.6)		--	--
Most backward caste	487 (24.6)	213 (43.7)	1.5 (0.8, 2.7)		--	--
Scheduled caste	439 (22.1)	198 (45.1)	1.5 (0.9, 2.6)		--	--
Scheduled tribes	402 (20.3)	161 (40.0)	1.3 (0.7, 2.5)		--	--
**House type§**						
Concrete	1054 (53.2)	417 (39.6)	REF	**0.01**		
Mixed	233 (11.7)	113 (48.5)	1.2 (1.1, 1.4)			
Government funded/ donated house	200 (10.1)	95 (47.5)	1.2 (1.0, 1.5)			
Thatched	496 (25.0)	224 (45.2)	1.1 (1.0, 1.3)			
**Socio-economic quintiles**						
Least poor quintile	422 (21.3)	110 (26.1)	REF	**<0.01**	REF	**<0.01**
4th quintile	390 (19.7)	161 (41.3)	1.6 (1.3, 1.9)		1.5 (1.2, 1.8)	
3th quintile	406 (20.5)	185 (45.6)	1.7 (1.4, 2.1)		1.6 (1.4, 2.0)	
2nd quintile	386 (19.5)	191 (49.5)	1.9 (1.5, 2.3)		1.9 (1.6, 2.3)	
Poorest quintile	379 (19.1)	202 (53.3)	2.0 (1.7, 2.5)		2.3 (2.0, 2.7)	
**Large household (family size)**						
No (<= 4 members)	1286 (64.9)	603 (46.9)	REF	**<0.01**		

For peer review only

Yes (>= 5 members)	697 (35.1)	246 (35.3)	0.8 (0.7, 0.8)			
Head of the household characteristics						
**Age**						
18-30 years	84 (4.2)	31 (36.9)	REF	**<0.01**		
31-40 years	388 (19.6)	142 (36.6)	1.0 (0.8, 1.3)			
41-50 years	516 (26.0)	185 (35.9)	1.0 (0.8, 1.2)			
51-60 years	431 (21.7)	184 (42.7)	1.2 (0.9, 1.5)			
>61 years	564 (28.4)	307 (54.4)	1.5 (1.1, 2.0)			
**Sex**						
Male	1602 (80.8)	638 (39.8)	REF	**<0.01**		
Female	381 (19.2)	211 (55.4)	1.4 (1.2, 1.6)			
**Education level**						
No education	587 (29.6)	317 (54.0)	REF	**<0.01**	REF	**<0.01**
Any primary	452 (22.8)	196 (43.4)	0.8 (0.7, 0.9)		0.8 (0.7, 0.9)	
Any middle	343 (17.3)	141 (41.1)	0.8 (0.6, 0.9)		0.8 (0.6, 0.9)	
Any secondary or higher	601 (30.3)	195 (32.4)	0.6 (0.5, 0.7)		0.7 (0.6, 0.8)	
**Marriage status**						
Never married	397 (20.0)	229 (57.7)	REF	**<0.01**		
Ever married	1586 (80.0)	620 (39.1)	0.7 (0.6, 0.8)			

Acronyms: PR - Prevalence Ratio; CI - Confidence ISnterval

†Number (N) of sampled households with complete covariate data used to estimate prevalence of non-response in each category.

‡Non-response - no household members available for interview, household refused to participate or unable to consent, or the household was not attempted.

§Concrete - concrete walls and roof; Mixed - concrete walls and tiled roof; Government donated/funded house - prebuilt government houses or houses funded through schemes for economically and socially marginalised groups; Thatched - thatched walls and roof.

For peer review only

Table 3: Factors associated with non-response in an in-person coverage evaluation survey in Vellore, India, 2019

	In-person coverage survey					
	**Households†**	**Non-response‡**	**Univariate**		**Multivariable**	
	**N=1976 (100) n (%)**	**n=211 (10.7) n (%)**	**PR (95% CI)**	**P value**	**PR (95% CI)**	**P value**
Site details						
**Proportion of households in each arm**						
Control	990 (50.1)	110 (11.1)	REF	0.65		
Intervention	986 (49.9)	101 (10.2)	0.9 (0.7, 1.3)			
**Proportion of households per site**						
Timiri	1581 (80.0)	187 (11.8)	REF	**<0.01**	REF	**<0.01**
Jawadhu Hills	395 (20.0)	24 (6.1)	0.5 (0.3, 0.8)		0.5 (0.3, 0.8)	
Household characteristics						
**Religion**						
Other	58 (2.9)	13 (22.4)	REF	**<0.01**		
Hindu	1918 (97.1)	198 (10.3)	0.5 (0.3, 0.7)			
**Caste**						
Higher caste	18 (0.9)	1 (5.6)	REF	0.18	--	--
Backward caste	609 (30.8)	73 (12.0)	2.2 (0.3, 16.0)		--	--
Most backward caste	502 (25.4)	62 (12.4)	2.2 (0.3, 16.2)		--	--
Scheduled caste	439 (22.2)	46 (10.5)	1.9 (0.2, 14.4)		--	--
Scheduled tribes	408 (20.6)	29 (7.1)	1.3 (0.2, 9.8)		--	--
**House type§**						
Concrete	1019 (51.6)	123 (12.1)	REF	**0.02**		
Mixed	229 (11.6)	26 (11.4)	0.9 (0.6, 1.4)			
Government funded/ donated house	191 (9.7)	24 (12.6)	1.0 (0.7, 1.5)			
Thatched	537 (27.2)	38 (7.1)	0.6 (0.4, 0.9)			
**Socio-economic quintiles**						
Least poor quintile	391 (19.8)	48 (12.3)	REF	**0.02**		
4th quintile	360 (18.2)	39 (10.8)	0.9 (0.6, 1.3)			
3th quintile	429 (21.7)	54 (12.6)	1.0 (0.7, 1.6)			
2nd quintile	410 (20.7)	47 (11.5)	0.9 (0.6, 1.5)			
Poorest quintile	386 (19.5)	23 (6.0)	0.5 (0.3, 0.8)			
**Large household (family size)**						

For peer review only

No ≤ 4 members)	1339 (67.8)	159 (11.9)	REF	**0.02**		
Yes (≥ 5 members)	637 (32.2)	52 (8.2)	0.7 (0.5, 0.9)			
Head of the household characteristics						
**Age**						
18-30 years	108 (5.5)	16 (14.8)	REF	**0.03**	REF	**<0.01**
31-40 years	403 (20.4)	55 (13.6)	0.9 (0.6, 1.5)		0.8 (0.5, 1.3)	
41-50 years	489 (24.7)	38 (7.8)	0.5 (0.3, 0.9)		0.4 (0.3, 0.7)	
51-60 years	455 (23.0)	53 (11.6)	0.8 (0.5, 1.3)		0.6 (0.4, 1.0)	
>61 years	521 (26.4)	49 (9.4)	0.6 (0.3, 1.2)		0.5 (0.3, 0.9)	
**Sex**						
Male	1567 (79.3)	155 (9.9)	REF	**0.02**		
Female	409 (20.7)	56 (13.7)	1.4 (1.1, 1.8)			
**Education level**						
No education	655 (33.1)	67 (10.2)	REF	0.36		
Any primary	427 (21.6)	48 (11.2)	1.1 (0.8, 1.5)			
Any middle	360 (18.2)	31 (8.6)	0.8 (0.5, 1.4)			
Any secondary or higher	534 (27.0)	65 (12.2)	1.2 (0.9, 1.6)			
**Marriage status**						
Never married	377 (19.1)	53 (14.1)	REF	**0.01**		
Ever married	1599 (80.9)	158 (9.9)	0.7 (0.5, 0.9)			

Acronyms: PR - Prevalence Ratio; CI - Confidence Interval

†Number (N) of sampled households with complete covariate data used to estimate prevalence of non-response in each category.
‡Non-response - no household members available for interview, household refused to participate or unable to consent, or the household was not attempted.
§Concrete - concrete walls and roof; Mixed - concrete walls and tiled roof; Government donated/funded house - prebuilt government houses or houses funded through schemes for economically and socially marginalised groups; Thatched - thatched walls and roof.

For peer review only

As a sensitivity analysis, we examined mobile phone ownership and the availability of phone numbers as additional candidate predictors. The final selected model for non-response in the phone-based survey included mobile phone ownership and SES, but it no longer included head of household education. Including mobile phone ownership attenuated but did not entirely remove the association of SES with non-response (households from the poorest SES quintile: PR: 1.7, 95% CI:1.4–2.1). Mobile phone

ownership was strongly associated with non-response in the phone-based survey independent of SES and adjusting for sub-site, but not in the in-person survey, for which the results did not change with the inclusion of mobile phone ownership as a candidate predictor (**Supplementary Tables 1 & 2**). Using phone number availability in place of mobile phone ownership, the predictor was again selected and had a slightly stronger association with non-response in the phone-based survey model, but there was no change in results for the in-person survey (**Supplementary Tables 3 & 4**). Cross-tabulation of phone number availability among those who reported owning a mobile phone showed that 93.5% (1461/1563) of the phone numbers were available in the in-person survey and decreased slightly to 89.6% (1524/1700) in the phone-based survey **(Supplementary Table 5).**

In 2020, 5728 (15.1%) out of the 37,883 censused households reported not owning a mobile phone. Households in the poorest quintile were 19 times more likely not to own a mobile phone than households in the least poor quintile (PR:19.3, 95% CI:15.3–24.3). Non-ownership of phones was less likely in households with heads who were more educated compared to those who reported no education ($p<0.01$). (**Supplementary Table 6).**

Differences in the implementation of in-person and phone-based surveys

For the in-person survey, 2932 attempts were made to reach the 2000 households, during 2376 (81.0%) of which household respondents were located and available to participate. In the phone-based survey, 3262 attempts were made to reach the 2000 households, during which primary respondents were located and available to participate in 1166 (35.7%) attempts **(Table 4).** The phone-based surveys were most

For peer review only - http://bmjopen.bmj.com/site/about/guidelines.xhtml

often conducted after 18:00 hrs, in contrast to the in-person interviews conducted mainly between 10:01 to 18:00 hrs (p<0.01). The mean duration of each in-person survey visit was longer (3.5 min) than the mean time taken to complete a call during the phone-based survey (1.9 min, p<0.01). During the in-person survey, each visit interviewed a median of 2.0 individuals per household or 66.7 of residents compared to each call during the phone-based survey, when a median of 0 was interviewed per call (which includes all the calls where nobody was present/could be reached) (p<0.01).

Table 4: Comparison of implementation of in-person and phone-based coverage surveys in Vellore, India, 2019-2020

	**In-person coverage survey**	**Phone-based coverage survey**	**P value***
Discrete household visits or calls	n=2932 (100.0)	n=3262 (100.0)	
**Household status during visit or call**			
Not located	2 (0.1)	1 (0.0)	
Members deceased	7 (0.2)	0 (0.0)	
Members not present	547 (18.7)	2095 (64.2)	**<0.01**
Members present	2376 (81.0)	1166 (35.7)	
**Time of day**			
<10:00	149 (5.1)	83 (2.5)	**<0.01**
10:01-12:00	656 (22.4)	398 (12.2)	
12:01-14:00	832 (28.4)	785 (24.1)	
14:01-16:00	659 (22.5)	535 (16.4)	
16:01-18:00	542 (18.5)	457 (14.0)	
>18:00	94 (3.2)	1004 (30.8)	
**Mean duration per visit or call (min)**	3.5 (1.8 - 6.9)	1.9 (1.0 - 4.4)	**<0.01**
**Median number of resident interviews completed per visit or call**	2.0 (1.0 - 4.0)	0.0 (0.0 - 3.0)	**<0.01**
**Median proportion of resident interviews completed per visit or call (IQR)**	66.7 (25.0 - 100.0)†	0.0 (0.0 - 100.0)†	**<0.01**
Household survey completion	n=1765 (100.0)	n=1134 (100.0)	
**Total duration to complete household survey (min)**	6.3 (3.6 - 10.6)	6.5 (3.8 - 10.8)	0.47
**Weighted mean total duration (min) ‡**	9.3 (9.0 - 9.7)	8.8 (8.4 - 9.2)	**0.05**

For peer review only - http://bmjopen.bmj.com/site/about/guidelines.xhtml

**Visits or calls to complete household survey**			
1	981 (55.6)	780 (68.8)	**<0.01**
2	730 (41.4)	174 (15.3)	
3	51 (2.9)	61 (5.4)	
4+	3 (0.2)	119 (10.5)	

*Pearson's chi-squared/T-test as appropriate. For status analysis, not located and members deceased were excluded due to small cell counts.

†Median (Interquartile range)

‡Weighted according to the household size

Most in-person survey households were completed in 1 or 2 visits (97.0%), but by phone, households were mainly completed in the first attempt (68.8%). Considering the household size, the weighted total time to complete the household's survey was slightly longer during the in-person survey (9.3 min) than in the phone-based survey (8.8 min, p=0.05) **(Table 4).**

DISCUSSION

In this study, we compared representativeness and implementation of a phone-based survey with an in-person survey to measure treatment coverage following cMDA for STH as part of the DeWorm3 trial in rural communities in southern India. Household participation in surveys assessing the coverage of the STH program was higher when conducted in person than when done using a phone-based survey (89.0% versus 57.2%). When comparing the characteristics of participating households, larger households, households with higher socioeconomic status and those with younger, more educated, and male heads of household were over-represented in the phone-based survey. Adjusting for sub-site, non-response in the phone-based survey was higher among households from the poorest SES quintile and households with

For peer review only

uneducated household heads, while non-response in the in-person survey was associated with the age of the household head.

We observed that households with higher socioeconomic status were overrepresented in the phone-based survey. Studies conducted in other low-middle-income countries have demonstrated that households with higher socioeconomic status are more likely to own mobile phones [27,28]. Furthermore, the phone-based survey was implemented during the COVID-19 lockdown in India when income and employment were drastically reduced, potentially increasing the challenges faced by poorer households to recharge phone subscriptions [29,30]. Both issues could likely explain lower overall participation rates among the poorest households in the phone-based survey, who were approximately half as likely to respond as the least poor households, based on the non-response model. Larger households were overrepresented in the phone-based survey, potentially because the probability of someone in the family owning a phone may be higher in such households, but this characteristic was not selected during modelling. Similar to our findings related to SES, studies in Germany and the UK also reported an under-representation of socially disadvantaged and/or low-income participants in phone-based surveys [8,10].

We also noted that participation of households headed by individuals in certain demographic groups differed between in-person and phone-based surveys. The phone-based survey had a higher proportion of respondents from households with younger, more educated, and male heads of household compared to the censused population. The effect of education was also noted in our model revealing that non-response in the phone-based survey was lower in households with heads who

For peer review only - http://bmjopen.bmj.com/site/about/guidelines.xhtml

reported any education compared to those who reported no education. The effect of age on participation in phone-based surveys has been well documented in related health surveys from the United States, Ireland, UK and Brazil [8,12,13]. In terms of gender, low phone ownership among women residing in South Asia has been reported previously due to traditional gender norms, technical illiteracy in operating a phone, male dominance over phone usage, and economic reasons [31].

Of the 1983 sampled households with complete data in the phone-based survey, 1685 (85.0%) had provided a mobile phone number during the annual census. The availability of a phone number does not always result in a response, however, often due to poor network coverage, unrenewed subscriptions, or changed subscriptions, as reported in a study on cell phone ownership in Burkina Faso [32]. From the starting sample of 2000 households, nearly twice as many households participated in the in-person survey compared to the phone-based survey. If information on population characteristics is available beforehand, researchers conducting phone-based surveys could consider quota sampling, where participants are selected non-randomly according to a fixed quota or percentage of the population based on one or more characteristics, or post-stratification weight adjustment following data collection to ensure that all age groups and socio-economic strata are equally represented [10,33]. Both methods can be employed if non-response remains high following quota sampling.

Interviewers initially contacted participants on the phone during the same working hours as the in-person survey. However, during implementation, we observed two distinct periods (mid-day between 12:01 to 14:00 and after 18:00 hrs) of response in

For peer review only

the phone-based survey (data not shown) and adapted the time of the calls to this observed pattern. Phone calls can occur in unexpected instances, and the owner may not be in a suitable environment to answer, such as during working hours, which can impact response rates [34]. Although phone-based surveys allowed us to adapt interview timings, we recommend considering the local culture and work patterns of the target population during planning to ensure high response rates. As observed in our study, phone-based surveys provide the advantage of flexibility in terms of survey timing, enabling data collection beyond the typical working hours and daylight constraints associated with in-person surveys, particularly in the context of safety considerations in many countries.

The in-person survey took marginally longer to complete as it necessitated the interviewer to verify and interview all available household members individually. This observed difference in duration would increase if the travel time to the house for an in-person survey, particularly in hard-to-reach rural areas, is considered. Arguably, the phone-based survey was faster to complete since one participant could respond on behalf of those absent, explaining higher completion rates on the first attempt. However, the number of attempts to establish a successful contact was higher in the phone-based survey because phone calls often failed to connect, similar to phone-based surveys conducted in Ireland and the UK [8].

NTDs are more prevalent in socially and economically disadvantaged groups living in poor environmental conditions [35]. In this population, STH infections, particularly hookworm, are more prevalent in households belonging to poorer SES quintiles with less-educated household heads [20]. MDA programs are one of the key public health

For peer review only - http://bmjopen.bmj.com/site/about/guidelines.xhtml

interventions implemented to control or eliminate NTDs, including STH, and a coverage survey following an MDA program evaluates the performance of the intervention. Therefore, it is imperative that the population included in a coverage evaluation survey does not exclude the same groups at a higher risk of infection to avoid biasing estimates of treatment coverage. In this regard, the phone-based survey under-represents many of these groups. Our findings suggest that in-person surveys better represent the target population in rural communities than phone-based surveys.

There are several advantages and limitations to this analysis. Our study within a trial setting provided the opportunity to compare both survey modes with exhaustive and up-to-date census information, allowing us to robustly assess the representativeness of both survey strategies. This analysis also offered an advantage over others based in low-and-middle-income countries since it was conducted in a rural setting with regular community engagement and used the same questionnaire in both surveys within a 6-month time frame, thus allowing us to compare participation confidently [36–38]. The phone-based survey was conducted in a state with high teledensity and fair network coverage, however, even in rural areas. As a result, these findings may not be generalisable to all rural settings. In addition, this survey was conducted during the COVID-19 pandemic and during a time of lockdowns in the study region, which may have negatively impacted responsiveness to the phone survey. Our exploration of representativeness and characteristics associated with non-response included a range of measures collected during the trial census, but there may be other unmeasured factors that we have not accounted for in our analyses.

CONCLUSIONS

For peer review only - http://bmjopen.bmj.com/site/about/guidelines.xhtml

We found that phone-based surveys underrepresent groups at a higher risk of acquiring STH and other NTDs. As a result, phone surveys may bias coverage estimates and could potentially negatively impact the groups that most need treatment. Phone-based surveys are a convenient alternative, especially when circumstances preclude household visits and resources are limited, but in the absence of accurate, up-to-date data on population characteristics, in-person surveys appear more representative.

For peer review only

Ethics statements

Patient consent for publication: Not required.

Ethics approval: The DeWorm3 study was approved by the Institutional Review Board of Christian Medical College, Vellore, India (10392, dated 30.11.2016), Human Subjects Division at the University of Washington (STUDY00000180) and The London School of Hygiene & Tropical Medicine (12013) and conformed to the principles embodied in the Declaration of Helsinki. For the in-person survey, written informed consent was sought from the head of the household or any adult member (≥18 years). For the phone-based surveys, the standardised information sheet was read out to the household head or the next eligible adult household member at the start of the call, and verbal consent over the phone was obtained. The change in protocol and current analysis was submitted to the Institutional Review Board of Christian Medical College, Vellore, as an amendment (IRB-A9-24.11.2021, dated 02.12.2022) and approved by The London School of Hygiene & Tropical Medicine (26333). The trial was registered at ClinicalTrials.gov (NCT03014167)

Acknowledgements

We thank field managers Rajeshkumar Rajendiran (Timiri) and Chinnaduraipandi (Jawadhu hills), Jasmine Farzana, Gokila Palaniswamy, Janarthanan Maniyarasu, Naveen Kumar Sekar and Dhanalakshmi Manoharan (at CMC, Vellore) and David S Kennedy (at LSHTM), for their meticulous work in developing and implementing the coverage data collection forms. We are most grateful to our field supervisors and field workers in Timiri and Jawadhu for exemplary work in completing the surveys despite the pandemic limitations. We acknowledge the contributions and efforts of the entire DeWorm3 Trials Team, particularly Dr Arianna Means, Dr Kristjana Ásbjörnsdóttir, and

For peer review only

Emily Pearman at the University of Washington and Dr Tim Littlewood and Leanne Doran at the Natural History Museum. We are thankful for support extended to this study from the Ministry of Health and Family Welfare, Delhi and the Directorate of Public Health, Chennai, as well as help in implementing study activities from officials at the district and block levels, village leaders and members of our community advisory boards in Timiri and Jawadhu hills. Most importantly, we thank all the participants for their time.

FOOTNOTES

Contributors: RMR and WEO conceptualised the study, conducted the data analysis, and wrote the first draft with support from SSRA and KEH. JLW, the Deworm3 study PI, obtained funding for the study and helped write the paper. KA, SPK and GJI conceptualised the study, oversaw the analysis, and provided feedback on the draft. SG and HL provided access to the data and supported critical interpretation of the findings. All authors (RMR, WEO, GJI, KA, SG, HL, SPK, JLW, KEH and SSRA) provided feedback on the manuscript and approved the final version.

Funding: The DeWorm3 study is funded through a grant to the University of Washington, Seattle, Washington, United States of America, from the Bill and Melinda Gates Foundation (OPP1129535, PI JLW). Under the grant conditions of the Foundation, a Creative Commons Attribution 4.0 Generic License has already been assigned to the Author Accepted Manuscript version that might arise from this submission. The funders were not involved in the decision to publish the manuscript and had no role in data collection, analysis or publication of study results.

Competing interests: None declared

Provenance and peer review: Not commissioned; externally peer-reviewed.

For peer review only

Supplemental material: This content has been supplied by the author(s). It has not been vetted by BMJ Publishing Group Limited (BMJ) and may not have been peer-reviewed. Any opinions or recommendations discussed are solely those of the author(s) and are not endorsed by BMJ. BMJ disclaims all liability and responsibility arising from any reliance on the content. Where the content includes any translated material, BMJ does not warrant the accuracy and reliability of the translations (including but not limited to local regulations, clinical guidelines, terminology, drug names and drug dosages) and is not responsible for any error or omissions arising from translation and adaptation or otherwise.

Twitter:

Rohan Michael Ramesh: @rohan_mic

William Edward Oswald: @williameoswald

Sitara S.R. Ajjampur: @ajjampur_s

ORCID iDs:

Rohan Michael Ramesh: 0000-0002-1089-6037

William Edward Oswald: 0000-0002-5287-2834

Sitara S.R. Ajjampur: 0000-0003-3419-6577

Figure Legends

Figure 1: Timeline of phone-based coverage evaluation survey in relation to other key activities

Figure 2: Household participation details of in-person and phone-based coverage surveys

Data availability statement

For peer review only

Data cannot be shared publicly because the study remains blinded to outcome data.

Following unblinding of the study, data will be available upon reasonable request.

References

1 Kempf AM, Remington PL. New Challenges for Telephone Survey Research in the Twenty-First Century. *Annu Rev Public Health* 2007;**28**:113–26. doi:10.1146/annurev.publhealth.28.021406.144059

2 Centre for Disease Control. Statistical methodology of the National Immunization Survey,1994-2002. CDC, 2005. Available:https://stacks.cdc.gov/view/cdc/6573 (accessed 1 Oct 2021).

3 Remington PL, Smith MY, Williamson DF, *et al.* Design, characteristics, and usefulness of state-based behavioral risk factor surveillance: 1981-87. *Public Health Rep* 1988;**103**:366–75.

4 Coffey D, Hathi P, Khalid N, *et al.* Using phone surveys to measure mental health. Ideas For India, 2020. Available: http://www.ideasforindia.in/topics/human-development/using-phone-surveys-to-measure-mental-health.html (accessed 1 Oct 2021).

5 Link MW, Mokdad AH. Effects of survey mode on self-reports of adult alcohol consumption: a comparison of mail, web and telephone approaches. *J Stud Alcohol* 2005;**66**:239–45.doi:10.15288/jsa.2005.66.239

6 The World Bank. Mobile cellular subscriptions (per 100 people). TWB, 2020. Available: https://data.worldbank.org/indicator/IT.CEL.SETS.P2?view=map (accessed 6 Nov 2021).

7 Gibson DG, Pereira A, Farrenkopf BA, *et al.* Mobile Phone Surveys for Collecting Population-Level Estimates in Low- and Middle-Income Countries: A Literature Review. *JMIR* 2017;**19**:e7428.doi:10.2196/jmir.7428

8 Boland M, Sweeney M, Scallan E, *et al.* Emerging advantages and drawbacks of telephone surveying in public health research in Ireland and the U.K. *BMC Public Health* 2006;**6**:208.doi:10.1186/1471-2458-6-208

9 Block ES, Erskine L. Interviewing by Telephone: Specific Considerations, Opportunities, and Challenges. *Int J Qual Methods* 2012;**11**(4):428–45.doi:10.1177/160940691201100409

10 Szolnoki G, Hoffmann D. Online, face-to-face and telephone surveys—Comparing different sampling methods in wine consumer research. *Wine Economics and Policy* 2013;**2**:57–66.doi:10.1016/j.wep.2013.10.001

11 Ellis CH, Krosnick JA. Comparing Telephone and Face-to-Face Surveys in Terms of Sample Representativeness: A Meta-Analysis of Demographic

For peer review only - http://bmjopen.bmj.com/site/about/guidelines.xhtml

Characteristics. American Nation election studies, 1999. Available: http://users.clas.ufl.edu/kenwald/pos6757/spring02/tch59.pdf (accessed 25 Aug 2020).

12 National Center for Health Statistics, Blumberg SJ, Lake JV. Wireless substitution : early release of estimates From the National Health Interview Survey, July-December 2020. National Center for Health Statistics, 2021. doi:10.15620/cdc:108678

13 Moura EC, Claro RM, Bernal R, *et al.* A feasibility study of cell phone and landline phone interviews for monitoring of risk and protection factors for chronic diseases in Brazil. *Cad Saúde Pública* 2011;**27**:277–86.doi:10.1590/S0102-311X2011000200009

14 Telecom Regulatory Authority of India. TRAI releases Telecom Subscription Data as on 31st July, 2021, TRAI 2021. Available: https://www.trai.gov.in/notifications/press-release/trai-releases-telecom-subscription-data-31st-july-2021 (accessed 1 Oct 2021).

15 International Institute for Population Sciences. National Family Health Survey (NFHS-4),India, 2015-16,IIPS 2017.Available: http://rchiips.org/nfhs/nfhs-4Reports/India.pdf (accessed 30 Apr 2020).

16 Crompton DWT. Preventive chemotherapy in human helminthiasis : coordinated use of anthelminthic drugs in control interventions : a manual for health professionals and programme managers. World Health Organization, 2006. Available:https://apps.who.int/iris/handle/10665/43545 (accessed 8 Nov 2021).

17 World Health Organisation. Preventive chemotherapy: Tools for improving the quality of reported data and information, 2019. Available:https://apps.who.int/iris/bitstream/handle/10665/329376/9789241516464-eng.pdf (accessed 8 Nov 2021).

18 USAID. Post MDA Survey Design: Monitoring treatment coverage of neglected disease control programs, in A toolkit developed for USAID Neglected Tropical Disease Control Project, 2009. Available:https://pdf.usaid.gov/pdf_docs/Pdacq848.pdf

19 World Health Organization. Monitoring and epidemiological assessment of mass drug administration in the global programme to eliminate lymphatic filariasis : a manual for national elimination programmes. *Suivi et évaluation épidémiologique du traitement médicamenteux de masse dans le cadre du Programme mondial pour l'élimination de la filariose lymphatique : manuel à l'intention des programmes nationauxd'élimination* Published Online First: 2011. Available: https://apps.who.int/iris/handle/10665/44580 (accessed 15 Nov 2021).

20 Ajjampur SSR, Kaliappan SP, Halliday KE, *et al.* Epidemiology of soil transmitted helminths and risk analysis of hookworm infections in the community: Results from the DeWorm3 Trial in southern India. *PLoS Negl Trop Dis* 2021;**15**:e0009338.doi:10.1371/journal.pntd.0009338

For peer review only - http://bmjopen.bmj.com/site/about/guidelines.xhtml

21 Ásbjörnsdóttir KH, Ajjampur SSR, Anderson RM, *et al.* Assessing the feasibility of interrupting the transmission of soil-transmitted helminths through mass drug administration: The DeWorm3 cluster randomized trial protocol. *PLoS Negl Trop Dis* 2018;**12**:e0006166.doi:10.1371/journal.pntd.0006166

22 DeWorm3 Trials Team. Baseline patterns of infection in regions of Benin, Malawi and India seeking to interrupt transmission of soil transmitted helminths (STH) in the DeWorm3 trial. *PLoS Negl Trop Dis* 2020;**14**:e0008771.doi:10.1371/journal.pntd.0008771

23 Evidence Action. World's Largest Deworming Program in India To Start With Support from Evidence Action, 2015. Available:https://www.evidenceaction.org/largest-deworming-program-in-india-to-start-with-support-from-evidence-action/ (accessed 31 Jan 2022).

24 National Health Portal Of India. National Deworming Day, 2021. Available: https://www.nhp.gov.in/national-deworming-day-2021_pg (accessed 6 Apr 2022).

25. Zou G. A modified poisson regression approach to prospective studies with binary data. *Am J Epidemiol*. 2004; **159**:702-6. doi: 10.1093/aje/kwh090.

26. Heinze G, Wallisch C, Dunkler D. Variable selection - A review and recommendations for the practicing statistician. *Biom J.* 2018;**60**:431-449. doi: 10.1002/bimj.201700067

27 Tran MC, Labrique AB, Mehra S, *et al.* Analyzing the Mobile "Digital Divide": Changing Determinants of Household Phone Ownership Over Time in Rural Bangladesh. *JMIR mHealth and uHealth* 2015;**3**:e3663.doi:10.2196/mhealth.3663

28 Labrique A, Blynn E, Ahmed S, *et al.* Health Surveys Using Mobile Phones in Developing Countries: Automated Active Strata Monitoring and Other Statistical Considerations for Improving Precision and Reducing Biases. *JMIR* 2017;**19**:e7329.doi:10.2196/jmir.7329

29 Kumar C. TRAI: India lost 1.7 crore mobile subscribers in lockdown months. The Times of India, 2020. Available: https://timesofindia.indiatimes.com/india/trai-india-lost-1-7-crore-mobile-subscribers-in-lockdown-months/articleshow/79273419.cms (accessed 8 Nov 2021).

30 Centre for Sustainable Employment. Covid-19 Employment and Income Tracker,2020. Available:https://cse.azimpremjiuniversity.edu.in/covid19-data-tracker/ (accessed 1 Dec 2021).

31 Giorgia Barboni, Erica Field, Rohini Pande, *et al.* A Tough Call: Understanding barriers to and impacts of women's mobile phone adoption in India. Harvard Kennedy School, 2018. Available: https://research.hks.harvard.edu/publications/getFile.aspx?Id=2765

For peer review only

32 Greenleaf AR, Ahmed S, Moreau C, *et al.* Cell phone ownership and modern contraceptive use in Burkina Faso: implications for research and interventions using mobile technology. *Contraception* 2019;**99**:170. doi:10.1016/j.contraception.2018.11.006

33. Quota Sampling. In: *The SAGE Encyclopedia of Educational Research, Measurement, and Evaluation*. 2455 Teller Road, Thousand Oaks, California 91320: SAGE Publications, Inc. 2018.doi:10.4135/9781506326139.n565

34 Dal Grande E, Chittleborough CR, Campostrini S, *et al.* Bias of health estimates obtained from chronic disease and risk factor surveillance systems using telephone population surveys in Australia: results from a representative face-to-face survey in Australia from 2010 to 2013. *BMC Med Res Methodol* 2016;**16**:44.doi:10.1186/s12874-016-0145-z

35 Aagaard-Hansen J, Chaignat CL. Neglected tropical diseases: equity and social determinants. World Health Organization 2010;**8**:135–157. Available:http://citeseerx.ist.psu.edu/viewdoc/download?doi=10.1.1.394.6922&rep=rep1&type=pdf (accessed 1 Dec 2021).

36 Nagpal K, Mathur MR, Biswas A, *et al*. Who do phone surveys miss, and how to reduce exclusion: recommendations from phone surveys in nine Indian states. *BMJ Glob Health* 2021;**6**:e005610.

37 Nambiar D, Benny G Usaidali. Telephony and trade-offs in fieldwork with the ‘unreached’: on the conduct of telephonic interviews with indigenous study participants in southern India. *BMJ Glob Health* 2021;**6:**e006261

38 Mohan D, Scott K, Shah N*, et al.* Can health information through mobile phones close the divide in health behaviours among the marginalised? An equity analysis of Kilkari in Madhya Pradesh, India. *BMJ Glob Health* 2021;**6:**e005512.

For peer review only

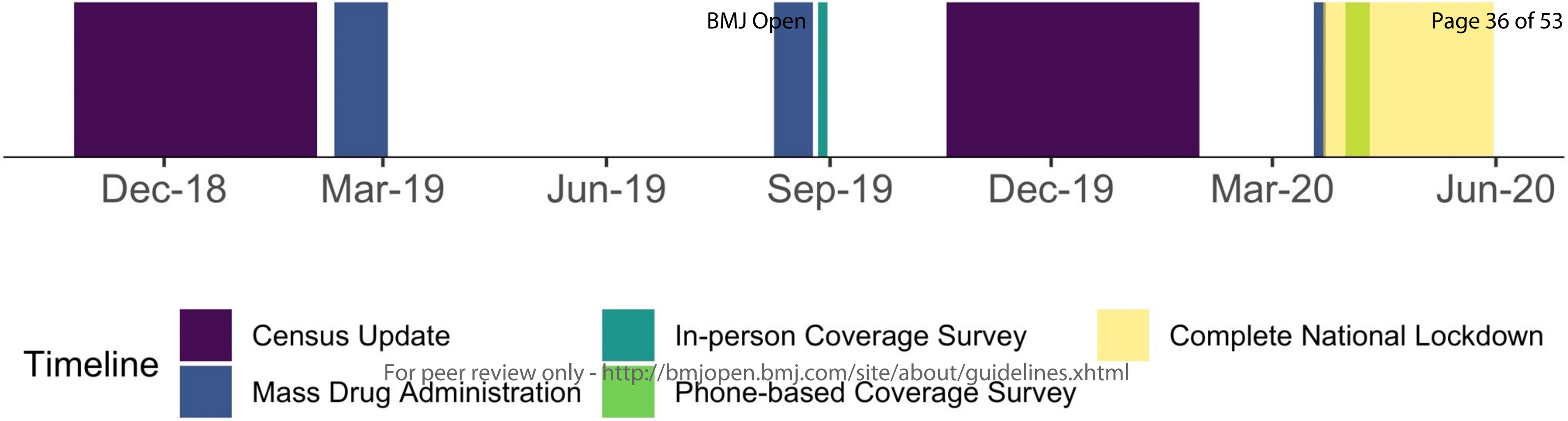

For peer review only - http://bmjopen.bmj.com/site/about/guidelines.xhtml

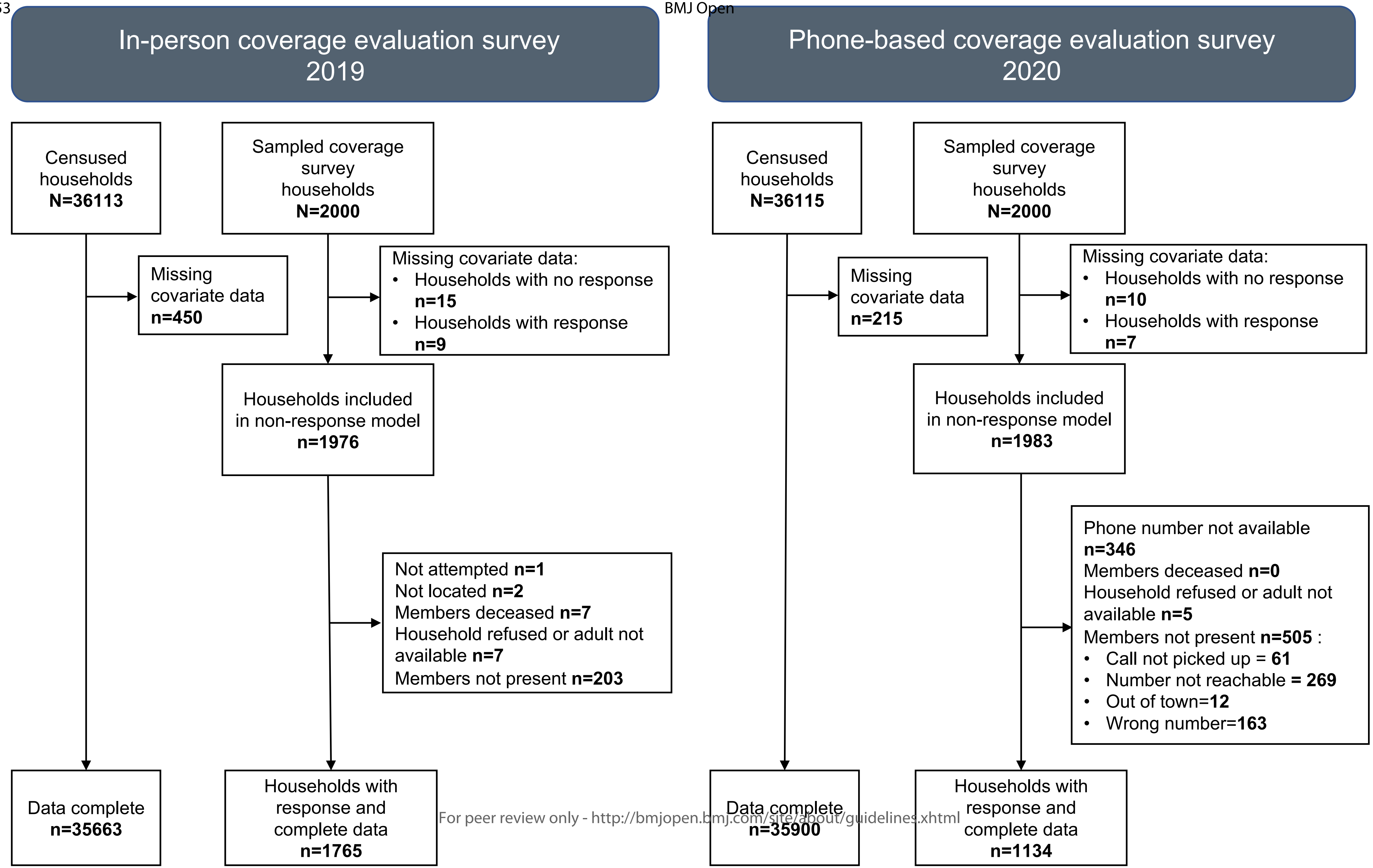
In-person coverage evaluation survey 2019
Censused households N=36113
Sampled coverage survey households N=2000
Missing covariate data n=450
Missing covariate data:
• Households with no response n=15
• Households with response n=9
Households included in non-response model n=1976
Not attempted n=1
Not located n=2
Members deceased n=7
Household refused or adult not available n=7
Members not present n=203
Data complete n=35663
Households with response and complete data n=1765
Phone-based coverage evaluation survey 2020
Censused households N=36115
Sampled coverage survey households N=2000
Missing covariate data n=215
Missing covariate data:
• Households with no response n=10
• Households with response n=7
Households included in non-response model n=1983
Phone number not available n=346
Members deceased n=0
Household refused or adult not available n=5
Members not present n=505 :
• Call not picked up = 61
• Number not reachable = 269
• Out of town=12
• Wrong number=163
Data complete n=35900
Households with response and complete data n=1134

Supplementary Table 1 - Factors associated with non-response in a phone-based coverage evaluation survey, including mobile phone ownership

	Phone-based coverage survey					
	Sampled households†	Non response‡	Univariate		Multivariable	
	N=1983 (100) n (%)	n=849 (42.8) n (%)	PR (95% CI)	P value	PR (95% CI)	P value
Site details						
**Proportion of households in each arm**						
Control	993 (50.1)	429 (43.2)	REF	0.78		
Intervention	990 (49.9)	420 (42.4)	1.0 (0.9, 1.1)			
**Proportion of households per site**						
Timiri	1586 (80.0)	694 (43.8)	REF	0.45	REF	**0.04**
Jawadhu Hills	397 (20.0)	155 (39.0)	0.9 (0.7, 1.2)		0.7 (0.6, 1.0)	
Household characteristics						
**Religion**						
Other	68 (3.4)	27 (39.7)	REF	0.67		
Hindu	1915 (96.6)	822 (42.9)	1.1 (0.8, 1.5)			
**Caste**						
Higher caste	20 (1.0)	6 (30.0)	REF	0.50		
Backward caste	635 (32.0)	271 (42.7)	1.4 (0.8, 2.6)			
Most backward caste	487 (24.6)	213 (43.7)	1.5 (0.8, 2.7)			
Scheduled caste	439 (22.1)	198 (45.1)	1.5 (0.9, 2.6)			
Scheduled tribes	402 (20.3)	161 (40.0)	1.3 (0.7, 2.5)			
**House type§**						
Concrete	1054 (53.2)	417 (39.6)	REF	**0.01**		
Mixed	233 (11.7)	113 (48.5)	1.2 (1.1, 1.4)			
Government funded/ donated house	200 (10.1)	95 (47.5)	1.2 (1.0, 1.5)			
Thatched	496 (25.0)	224 (45.2)	1.1 (1.0, 1.3)			
**Socio-economic quintiles**						
Least poorest quintile	422 (21.3)	110 (26.1)	REF	**<0.01**	REF	**<0.01**
4th quintile	390 (19.7)	161 (41.3)	1.6 (1.3, 1.9)		1.5 (1.2, 1.8)	
3rd quintile	406 (20.5)	185 (45.6)	1.7 (1.4, 2.1)		1.5 (1.3, 1.9)	
2th quintile	386 (19.5)	191 (49.5)	1.9 (1.5, 2.3)		1.6 (1.4, 1.9)	
Poorest quintile	379 (19.1)	202 (53.3)	2.0 (1.7, 2.5)		1.7 (1.4, 2.1)	
**Large household (family size)**						
No (<= 4 members)	1286 (64.9)	603 (46.9)	REF	**<0.01**		

Yes (>= 5 members)	697 (35.1)	246 (35.3)	0.8 (0.7, 0.8)			
**Owns mobile phone**						
No	298 (15.0)	255 (85.6)	REF	**<0.01**	REF	**<0.01**
Yes	1685 (85.0)	594 (35.3)	0.4 (0.4, 0.5)		0.5 (0.4, 0.5)	
Head of the household characteristics						
**Age**						
18-30 years	84 (4.2)	31 (36.9)	REF	**<0.01**		
31-40 years	388 (19.6)	142 (36.6)	1.0 (0.8, 1.3)			
41-50 years	516 (26.0)	185 (35.9)	1.0 (0.8, 1.2)			
51-60 years	431 (21.7)	184 (42.7)	1.2 (0.9, 1.5)			
>61 years	564 (28.4)	307 (54.4)	1.5 (1.1, 2.0)			
**Sex**						
Male	1602 (80.8)	638 (39.8)	REF	<0.01		
Female	381 (19.2)	211 (55.4)	1.4 (1.2, 1.6)			
**Education level**						
No education	587 (29.6)	317 (54.0)	REF	**<0.01**		
Any primary	452 (22.8)	196 (43.4)	0.8 (0.7, 0.9)			
Any middle	343 (17.3)	141 (41.1)	0.8 (0.6, 0.9)			
Any secondary or higher	601 (30.3)	195 (32.4)	0.6 (0.5, 0.7)			
**Marriage status**						
Never married	397 (20.0)	229 (57.7)	REF	**<0.01**		
Ever married	1586 (80.0)	620 (39.1)	0.7 (0.6, 0.8)			

Acronyms: PR - Prevalence Ratio; CI - Confidence Interval

†Number (N) of sampled households with complete covariate data used to estimate prevalence of non-response in each category.

‡Non-response - no household members available for interview, household refused to participate or unable to consent, or the household was not attempted.

§Concrete - concrete walls and roof; Mixed - concrete walls and tiled roof; Government donated/funded house - prebuilt government houses or houses funded through schemes for economically and socially marginalized groups; Thatched - thatched walls and roof.

For peer review only

Supplementary Table 2 - Factors associated with non-response in an in-person coverage evaluation survey, including mobile phone ownership

	In-person coverage survey					
	**Sampled households†**	**Non response‡**	**Univariate**		**Multivariable**	
	**N=1976 (100) n (%)**	**n=211 (10.7) n (%)**	**PR (95% CI)**	**P value**	**PR (95% CI)**	**P value**
Site details						
**Proportion of households in each arm**						
Control	990 (50.1)	110 (11.1)	REF	0.65		
Intervention	986 (49.9)	101 (10.2)	0.9 (0.7, 1.3)			
**Proportion of households per site**						
Timiri	1581 (80.0)	187 (11.8)	REF	**<0.01**	REF	**<0.01**
Jawadhu Hills	395 (20.0)	24 (6.1)	0.5 (0.3, 0.8)		0.5 (0.3, 0.8)	
Household characteristics						
**Religion**						
Other	58 (2.9)	13 (22.4)	REF	**<0.01**		
Hindu	1918 (97.1)	198 (10.3)	0.5 (0.3, 0.7)			
**Caste**						
Higher caste	18 (0.9)	1 (5.6)	REF	0.18		
Backward caste	609 (30.8)	73 (12.0)	2.2 (0.3, 16.0)			
Most backward caste	502 (25.4)	62 (12.4)	2.2 (0.3, 16.2)			
Scheduled caste	439 (22.2)	46 (10.5)	1.9 (0.2, 14.4)			
Scheduled tribes	408 (20.6)	29 (7.1)	1.3 (0.2, 9.8)			
**House type§**						
Concrete	1019 (51.6)	123 (12.1)	REF	0.02		
Mixed	229 (11.6)	26 (11.4)	0.9 (0.6, 1.4)			
Government funded/ donated house	191 (9.7)	24 (12.6)	1.0 (0.7, 1.5)			
Thatched	537 (27.2)	38 (7.1)	0.6 (0.4, 0.9)			
**Socio-economic quintiles**						
Least poorest quintile	391 (19.8)	48 (12.3)	REF	**0.02**		
4th quintile	360 (18.2)	39 (10.8)	0.9 (0.6, 1.3)			
3th quintile	429 (21.7)	54 (12.6)	1.0 (0.7, 1.6)			
2th quintile	410 (20.7)	47 (11.5)	0.9 (0.6, 1.5)			
Poorest quintile	386 (19.5)	23 (6.0)	0.5 (0.3, 0.8)			
**Large household (family size)**						

For peer review only

No (<= 4 members)	1339 (67.8)	159 (11.9)	REF	**0.02**		
Yes (>= 5 members)	637 (32.2)	52 (8.2)	0.7 (0.5, 0.9)			
**Owns mobile phone**						
No	293 (14.8)	25 (8.5)	REF	0.16		
Yes	167 (10.8)	1.3 (0.9, 1.8)	1.3 (0.9, 1.8)			
Data not available	134 (6.8)	19 (14.2)	1.7 (1.0, 2.8)			
Head of the household characteristics						
**Age**						
18-30 years	108 (5.5)	16 (14.8)	REF	**0.03**	REF	**<0.01**
31-40 years	403 (20.4)	55 (13.6)	0.9 (0.6, 1.5)		0.8 (0.5, 1.3)	
41-50 years	489 (24.7)	38 (7.8)	0.5 (0.3, 0.9)		0.4 (0.3, 0.7)	
51-60 years	455 (23.0)	53 (11.6)	0.8 (0.5, 1.3)		0.6 (0.4, 1.0)	
>61 years	521 (26.4)	49 (9.4)	0.6 (0.3, 1.2)		0.5 (0.3, 0.9)	
**Sex**						
Male	1567 (79.3)	155 (9.9)	REF	**0.02**		
Female	409 (20.7)	56 (13.7)	1.4 (1.1, 1.8)			
**Education level**						
No education	655 (33.1)	67 (10.2)	REF	0.36		
Any primary	427 (21.6)	48 (11.2)	1.1 (0.8, 1.5)			
Any middle	360 (18.2)	31 (8.6)	0.8 (0.5, 1.4)			
Any secondary or higher	534 (27.0)	65 (12.2)	1.2 (0.9, 1.6)			
**Marriage status**						
Never married	377 (19.1)	53 (14.1)	REF	**0.01**		
Ever married	1599 (80.9)	158 (9.9)	0.7 (0.5, 0.9)			

Acronyms: PR - Prevalence Ratio; CI - Confidence Interval

†Number (N) of sampled households with complete covariate data used to estimate prevalence of non-response in each category.
‡Non-response - no household members available for interview, household refused to participate or unable to consent, or the household was not attempted.
§Concrete - concrete walls and roof; Mixed - concrete walls and tiled roof; Government donated/funded house - prebuilt government houses or houses funded through schemes for economically and socially marginalized groups; Thatched - thatched walls and roof.

For peer review only

Supplementary Table 3 - Factors associated with non-response in a phone-based coverage survey, including phone number availability

	Phone-based coverage survey					
	**Sampled households†**	**Non response‡**	**Univariate**		**Multivariable**	
	**N=1983 (100) n (%)**	**n=849 (42.8) n (%)**	**PR (95% CI)**	**P value**	**PR (95% CI)**	**P value**
Site details						
**Proportion of households in each arm**						
Control	993 (50.1)	429 (43.2)	REF	0.78		
Intervention	990 (49.9)	420 (42.4)	1.0 (0.9, 1.1)			
**Proportion of households per site**						
Timiri	1586 (80.0)	694 (43.8)	REF	0.45	REF	**0.08**
Jawadhu Hills	397 (20.0)	155 (39.0)	0.9 (0.7, 1.2)		0.8 (0.6, 1.0)	
Household characteristics						
**Religion**						
Other	68 (3.4)	27 (39.7)	REF	0.67		
Hindu	1915 (96.6)	822 (42.9)	1.1 (0.8, 1.5)			
**Caste**						
Higher caste	20 (1.0)	6 (30.0)	REF	0.50		
Backward caste	635 (32.0)	271 (42.7)	1.4 (0.8, 2.6)			
Most backward caste	487 (24.6)	213 (43.7)	1.5 (0.8, 2.7)			
Scheduled caste	439 (22.1)	198 (45.1)	1.5 (0.9, 2.6)			
Scheduled tribes	402 (20.3)	161 (40.0)	1.3 (0.7, 2.5)			
**House type§**						
Concrete	1054 (53.2)	417 (39.6)	REF	**0.01**		
Mixed	233 (11.7)	113 (48.5)	1.2 (1.1, 1.4)			
Government funded/ donated house	200 (10.1)	95 (47.5)	1.2 (1.0, 1.5)			
Thatched	496 (25.0)	224 (45.2)	1.1 (1.0, 1.3)			
**Socio-economic quintiles**						
Least poorest quintile	422 (21.3)	110 (26.1)	REF	**<0.01**	REF	**<0.01**
4th quintile	390 (19.7)	161 (41.3)	1.6 (1.3, 1.9)		1.5 (1.3, 1.9)	
3th quintile	406 (20.5)	185 (45.6)	1.7 (1.4, 2.1)		1.5 (1.2, 1.7)	
2th quintile	386 (19.5)	191 (49.5)	1.9 (1.5, 2.3)		1.6 (1.4, 1.9)	
Poorest quintile	379 (19.1)	202 (53.3)	2.0 (1.7, 2.5)		1.7 (1.4, 2.0)	
**Large household (family size)**						
No (<= 4 members)	1286 (64.9)	603 (46.9)	REF	**<0.01**		

For peer review only

Yes (>= 5 members)	697 (35.1)	246 (35.3)	0.8 (0.7, 0.8)			
**Phone number available**						
No	405 (20.4)	382 (94.3)	REF	**<0.01**	REF	**<0.01**
Yes	1578 (79.6)	467 (29.6)	0.3 (0.3, 0.4)		0.3 (0.3, 0.4)	
Head of the household characteristics						
**Age**						
18-30 years	84 (4.2)	31 (36.9)	REF	**<0.01**		
31-40 years	388 (19.6)	142 (36.6)	1.0 (0.8, 1.3)			
41-50 years	516 (26.0)	185 (35.9)	1.0 (0.8, 1.2)			
51-60 years	431 (21.7)	184 (42.7)	1.2 (0.9, 1.5)			
>61 years	564 (28.4)	307 (54.4)	1.5 (1.1, 2.0)			
**Sex**						
Male	1602 (80.8)	638 (39.8)	REF	**<0.01**		
Female	381 (19.2)	211 (55.4)	1.4 (1.2, 1.6)			
**Education level**						
No education	587 (29.6)	317 (54.0)	REF	**<0.01**		
Any primary	452 (22.8)	196 (43.4)	0.8 (0.7, 0.9)			
Any middle	343 (17.3)	141 (41.1)	0.8 (0.6, 0.9)			
Any secondary or higher	601 (30.3)	195 (32.4)	0.6 (0.5, 0.7)			
**Marriage status**						
Never married	397 (20.0)	229 (57.7)	REF	**<0.01**		
Ever married	1586 (80.0)	620 (39.1)	0.7 (0.6, 0.8)			

Acronyms: PR - Prevalence Ratio; CI - Confidence Interval

†Number (N) of sampled households with complete covariate data used to estimate prevalence of non-response in each category.
‡Non-response - no household members available for interview, household refused to participate or unable to consent, or the household was not attempted.
§Concrete - concrete walls and roof; Mixed - concrete walls and tiled roof; Government donated/funded house - prebuilt government houses or houses funded through schemes for economically and socially marginalized groups; Thatched - thatched walls and roof.

For peer review only

Supplementary Table 4 - Factors associated with non-response in an in-person coverage survey, including phone number availability

	In-person coverage survey					
	**Sampled households†**	**Non response‡**	**Univariate**		**Multivariable**	
	**N=1976 (100) n (%)**	**n=211 (10.7) n (%)**	**PR (95% CI)**	**P value**	**aPR (95% CI)**	**P value**
Site details						
**Proportion of households in each arm**						
Control	990 (50.1)	110 (11.1)	REF	0.65		
Intervention	986 (49.9)	101 (10.2)	0.9 (0.7, 1.3)			
**Proportion of households per site**						
Timiri	1581 (80.0)	187 (11.8)	REF	**<0.01**	REF	**<0.01**
Jawadhu Hills	395 (20.0)	24 (6.1)	0.5 (0.3, 0.8)		0.5 (0.3, 0.8)	
Household characteristics						
**Religion**						
Other	58 (2.9)	13 (22.4)	REF	**<0.01**		
Hindu	1918 (97.1)	198 (10.3)	0.5 (0.3, 0.7)			
**Caste**						
Higher caste	18 (0.9)	1 (5.6)	REF	0.18		
Backward caste	609 (30.8)	73 (12.0)	2.2 (0.3, 16.0)			
Most backward caste	502 (25.4)	62 (12.4)	2.2 (0.3, 16.2)			
Scheduled caste	439 (22.2)	46 (10.5)	1.9 (0.2, 14.4)			
Scheduled tribes	408 (20.6)	29 (7.1)	1.3 (0.2, 9.8)			
**House type§**						
Concrete	1019 (51.6)	123 (12.1)	REF	0.02		
Mixed	229 (11.6)	26 (11.4)	0.9 (0.6, 1.4)			
Government funded/ donated house	191 (9.7)	24 (12.6)	1.0 (0.7, 1.5)			
Thatched	537 (27.2)	38 (7.1)	0.6 (0.4, 0.9)			
**Socio-economic quintiles**						
Least poor quintile	391 (19.8)	48 (12.3)	REF	**0.02**		
4th quintile	360 (18.2)	39 (10.8)	0.9 (0.6, 1.3)			
3th quintile	429 (21.7)	54 (12.6)	1.0 (0.7, 1.6)			
2th quintile	410 (20.7)	47 (11.5)	0.9 (0.6, 1.5)			
Poorest quintile	386 (19.5)	23 (6.0)	0.5 (0.3, 0.8)			
**Large household (family size)**						
No (<= 4 members)	1339 (67.8)	159 (11.9)	REF	**0.02**		

Yes (>= 5 members)	637 (32.2)	52 (8.2)	0.7 (0.5, 0.9)			
**Phone number available**						
No	273 (13.8)	26 (9.5)	REF	**<0.01**		
Yes	1703 (86.2)	185 (10.9)	1.1 (0.8, 1.6)			
Head of the household characteristics						
**Age**						
18-30 years	108 (5.5)	16 (14.8)	REF	**0.03**	REF	**<0.01**
31-40 years	403 (20.4)	55 (13.6)	0.9 (0.6, 1.5)		0.8 (0.5, 1.3)	
41-50 years	489 (24.7)	38 (7.8)	0.5 (0.3, 0.9)		0.4 (0.3, 0.7)	
51-60 years	455 (23.0)	53 (11.6)	0.8 (0.5, 1.3)		0.6 (0.4, 1.0)	
>61 years	521 (26.4)	49 (9.4)	0.6 (0.3, 1.2)		0.5 (0.3, 0.9)	
**Sex**						
Male	1567 (79.3)	155 (9.9)	REF	**0.02**		
Female	409 (20.7)	56 (13.7)	1.4 (1.1, 1.8)			
**Education level**						
No education	655 (33.1)	67 (10.2)	REF	0.36		
Any primary	427 (21.6)	48 (11.2)	1.1 (0.8, 1.5)			
Any middle	360 (18.2)	31 (8.6)	0.8 (0.5, 1.4)			
Any secondary or higher	534 (27.0)	65 (12.2)	1.2 (0.9, 1.6)			
**Marriage status**						
Never married	377 (19.1)	53 (14.1)	REF	**0.01**		
Ever married	1599 (80.9)	158 (9.9)	0.7 (0.5, 0.9)			

Acronyms: PR - Prevalence Ratio; CI - Confidence Interval

†Number (N) of sampled households with complete covariate data used to estimate prevalence of non-response in each category.
‡Non-response - no household members available for interview, household refused to participate or unable to consent, or the household was not attempted.
§Concrete - concrete walls and roof; Mixed - concrete walls and tiled roof; Government donated/funded house - prebuilt government houses or houses funded through schemes for economically and socially marginalized groups; Thatched - thatched walls and roof.

For peer review only

Supplementary Table 5 - Phone number availability among mobile phone ownership reported in the annual census

Owns mobile phone	In-person coverage survey households (n=1563)	Phone-based coverage survey households (n=1700)	P value
Household phone number available			
**No**	102 (6.5)	176 (10.4)	**<0.01**
**Yes**	1461 (93.5)	1524 (89.6)	

For peer review only

Supplementary Table 6 - Factors associated with reported non-ownership of a mobile phone in Tamil Nadu, 2019

	Censused households 2019-2020 N=37883 (100) n (%)	Non ownership N=5728 (15.1) n (%)	Univariate PR (95% CI)	P value	Multivariable PR (95% CI)	P value
Site details						
**Proportion of households in each arm**						
Control	18424 (48.6)	2800 (15.2)	REF	0.91		
Intervention	19459 (51.4)	2928 (15.0)	1.0 (0.8, 1.2)			
**Proportion of households per site**						
Timiri	29545 (78.0)	4159 (14.1)	REF	**<0.01**	REF	**<0.01**
Jawadhu Hills	8338 (22.0)	1569 (18.8)	1.3 (1.1, 1.7)		0.4 (0.3, 0.4)	
Household characteristics						
**Religion**						
Other	1133 (3.0)	93 (8.2)	REF	**<0.01**		
Hindu	36750 (97.0)	5635 (15.3)	1.9 (1.5, 2.4)			
**Caste**						
Higher	430 (1.1)	50 (11.6)	REF	**<0.01**		
Backward	11362 (30.0)	1406 (12.4)	1.1 (0.8, 1.5)			
Most backward	9207 (24.3)	1334 (14.5)	1.2 (0.9, 1.8)			
Scheduled	8457 (22.3)	1264 (14.9)	1.3 (0.9, 1.8)			
Scheduled tribes	8427 (22.2)	1674 (19.9)	1.7 (1.2, 2.4)			
**House type†**						
Concrete	20055 (52.9)	1861 (9.3)	REF	**<0.01**		
Mixed	4190 (11.1)	750 (17.9)	1.9 (1.8, 2.1)			
Government funded/ donated house	3518 (9.3)	626 (17.8)	1.9 (1.7, 2.1)			
Thatched	10120 (26.7)	2491 (24.6)	2.7 (2.4, 2.9)			
**Socio-economic quintiles**						
Least poor quintile	7573 (20.0)	132 (1.7)	REF	**<0.01**	REF	**<0.01**
4th quintile	7569 (20.0)	302 (4.0)	2.3 (1.8, 2.9)		2.0 (1.6, 2.5)	
3th quintile	7540 (19.9)	876 (11.6)	6.7 (5.3, 8.4)		5.2 (4.1, 6.5)	
2th quintile	7598 (20.1)	1750 (23.0)	13.2 (9.9, 17.7)		10.9 (8.5, 14.1)	
Poorest quintile	7603 (20.1)	2668 (35.1)	20.1 (15.4, 26.3)		19.3 (15.3, 24.3)	
**Large household (family size)**						

For peer review only

No (<= 4 members)	24965 (65.9)	4947 (19.8)	REF	**<0.01**		
Yes (>= 5 members)	12918 (34.1)	781 (6.0)	0.3 (0.3, 0.4)			
**Head of the household characteristics**						
**Age**						
18-30 years	1600 (4.2)	144 (9.0)	REF	**<0.01**		
31-40 years	7230 (19.1)	538 (7.4)	0.8 (0.7, 1.0)			
41-50 years	9708 (25.6)	783 (8.1)	0.9 (0.7, 1.1)			
51-60 years	8848 (23.4)	1160 (13.1)	1.5 (1.1, 1.9)			
>61 years	10497 (27.7)	3103 (29.6)	3.3 (2.5, 4.2)			
**Sex**						
Male	30800 (81.3)	3203 (10.4)	REF	**<0.01**		
Female	7083 (18.7)	2525 (35.6)	3.4 (3.1, 3.7)			
**Education level**						
No education	11581 (30.6)	3518 (30.4)	REF	**<0.01**	REF	**<0.01**
Any primary	8613 (22.7)	1240 (14.4)	0.5 (0.4, 0.5)		0.6 (0.6, 0.6)	
Any middle	6762 (17.8)	517 (7.6)	0.3 (0.2, 0.3)		0.4 (0.3, 0.4)	
Any secondary or higher	10927 (28.8)	453 (4.1)	0.1 (0.1, 0.2)		0.3 (0.3, 0.3)	
**Marriage status**						
Never married	7843 (20.7)	2750 (35.1)	REF	**<0.01**		
Ever married†	30040 (79.3)	2978 (9.9)	0.3 (0.3, 0.3)			

Acronyms: PR - Prevalence Ratio; CI - Confidence Interval

† Concrete - concrete walls and roof; Mixed - concrete walls and tiled roof; Government donated/funded house - prebuilt government houses or houses funded through schemes for economically and socially marginalized groups; Thatched - thatched walls and roof

For peer review only

Coverage Evaluation Survey Questionnaire

1	Name of the participant		
2	Sex if the participant		
3	Age of the participant in years		
4.1	Can the participant answer the questions now?	1	Yes, agrees to participate
		2	Not present
		3	No, refused
		4	No, other
		5	Proxy respondent
4.2	Why has household refused to participate?	1	Refused to speak/Not interested
		2	Prior negative experience
		3	Unwilling to provide detailed household information
		4	Unwilling because of concerns related to risks, confidentiality, etc.
		5	Unwilling to sign/provide thumb impression
		6.	Other
4.3	If unavailable, where is the participant?	1	At school
		2	At college
		3	At work
		4	Not at home (e.g at shop)
		5	Moved away permanently
		6	Temporarily migrated
		7	Died
		8.	Other
5	Name of the proxy respondent		
6	Sex of the proxy respondent		
7	Age of the proxy respondent		
8	Did someone give you a tablet in the past month to treat worms (at home, at school, or in the community)?	1	Yes
		2	No
		3.	Don't know/Cannot recall
		4.	Refused
8.1	If 'No', Why didn't you receive the tablet?	1	Unaware of MDA
		2	Distributor did not come to my community
		3	Distributor came to my community but did not come to my house
		4	Distributor did not come to my school
		5	The distributor was out of tablets and did not return
		6	Pregnant
		7	Breastfeeding
		8	Ill
		9	Told I could not take a tablet that day for other reasons
		10	Too old
		11	Underage (less than 1yo)
		12	I was busy
		13	I was too far away
		14	Other
		15	Don't know/Cannot recall
		16	Refused
8.2	If 'Yes', Did you swallow the tablet?	1	Yes
		2	No
		3	Don't know/Cannot recall
		4	Refused
8.3	Why did you not swallow the tablet?	1	Pregnant
		2	Breastfeeding
		3	Ill
		4	Too old
		5	Underage (less than 1yo)
		6	Afraid of side effects
		7	I am not at risk for this disease
		8	I am taking other medications
		9	Medicine doesn't work
		10	Other
		11	Don't know/Cannot recall
		12	Refused
8.4	Why did you swallow the tablet?	1	To be healthy
		2	Afraid of getting the disease
		3	Someone told me to
		4	Everyone else is taking it
		5	Don't know/Cannot recall

For peer review only

For peer review only - http://bmjopen.bmj.com/site/about/guidelines.xhtml

		6	Refused
		7	Other
9	Did you swallow the tablet in front of the person who gave it to you?	1	Yes
		2	No
		3	Don't know/Cannot recall
		4	Refused
10	Who gave you the tablet?	1	Family member
		2	Health staff
		3	Field worker
		4	Drug distributor
		5	Community leader
		6	Teacher /Anganwadi worker
		7	Other
		8	Don't know/Cannot recall
		9	Refused
11	Where did they give you the tablet?	1	At home
		2	At school
		3	At Anganwadi centre
		4	Elsewhere in community
		5	Other
		6	Don't know/Cannot recall
		7	Refused
12	Did you feel any side effects after swallowing the tablet?	1	Yes
		2	No
		3	Don't know/Cannot recall
		4	Refused
13.1	Which of the following statements are true about side effects you experienced after swallowing the tablet?	1	Mild (did not interfere with daily activities)
		2	Moderate (interfered with daily activities)
		3	Serious (required hospitalization)
		4	Don't know/Cannot recall
		5	Refused
14	How many of these tablets did you swallow?	1	1/2
		2	1
		3	2
		4	3
		5	4
		6	5+
		7	Don't know/Cannot recall
		8	Refused
15	Were you aware that tablets would be distributed?	1	Yes
		2	No
		3	Don't know/Cannot recall
		4	Refused
16	How did you know that the tablets would be distributed?	1	Family member/friend/neighbor
		2	Professional health staff
		3	Community drug distributor/community health worker/teacher
		4	Community or religious leader
		5	Brochures/flyers
		6	Posters
		7	Banners
		8	Radio
		9	TV
		10	Social media
		11	Community Theatre/Drama
		12	Gram sabha
		13	Don't know/Cannot recall
		14	Refused to answer
		15	Other
17	Did any of your neighbors take the distributed tablet?	1	Yes
		2	No
		3	Don't know/Cannot recall
		4	Refused
18	What did you like about the community treatment programme?	1	House to house treatment makes it easy
		2	Trusted distributors
		3	No long wait for drugs
		4	Free drugs
		5	No specific part I liked
		6	Don't know/Cannot recall
		7	Refused to answer

For peer review only

		8	Other
19	What did you not like about the community treatment programme?	1	Inconvenient time
		2	Drugs ran out or were not available
		3	Unfriendly distributor
		4	Took too much time
		5	Adverse reaction to drug
		6	Did not also give treatment for other diseases
		7	No specific part I disliked
		8	Don't know/Cannot recall
		9	Refused to answer
		10	Other
20	Next time the tablets are given, how would you want them distributed?	1	The same door-to-door
		2	All individuals go to school
		3	Distributors in central places in the village
		4	Don't know/Cannot recall
		5	Refused to answer
		6	Other
21	Did you have to change your daily routine to participate in the treatment day?	1	Yes
		2	No
		3	Don't know/Cannot recall
		4	Refused
22	About how much of your time did it take to participate in the treatment day, including the time you spent waiting for the drug distributor to arrive?	1	Less than one hour
		2	About one hour
		3	Half of a day
		4	A full day
		5	More than one day, distributor did not come on day planned
		6	Don't know/Cannot recall
		7	Refused to say
		8	Other
23	About how many minutes did the drug distributor spend at your house on the treatment day?	1	Less than 5 minutes
		2	About 10 minutes
		3	About 20 minutes
		4	About 30 minutes
		5	About 45 minutes
		6	About one hour
		7	More than one hour
		8	Drugs were left here when we were not home
		9	Don't know/Cannot recall
		10	Refused to answer
		11	Other
24	If there are no adult members available, where are they?	1	Not at home (e.g., at shop)
		2	Moved away permanently
		3	Temporarily migrated

For peer review only

STROBE Statement—Checklist of items that should be included in reports of ***cross-sectional studies***

	Item No	Recommendation	Page No
**Title and abstract**	1	*(a)* Indicate the study's design with a commonly used term in the title or the abstract **DONE – in the Abstract**	3
		(b) Provide in the abstract an informative and balanced summary of what was done and what was found **DONE – in the Abstract**	3
**Introduction**			
Background/rationale	2	Explain the scientific background and rationale for the investigation being reported **DONE – See Introduction section**	5-6
Objectives	3	State specific objectives, including any prespecified hypotheses **DONE – See Introduction section**	6
**Methods**			
Study design	4	Present key elements of study design early in the paper **DONE – See Methods section**	7-9
Setting	5	Describe the setting, locations, and relevant dates, including periods of recruitment, exposure, follow-up, and data collection **DONE – See Methods section**	7-9
Participants	6	*(a)* Give the eligibility criteria, and the sources and methods of selection of participants **DONE – See Methods section**	7-9
Variables	7	Clearly define all outcomes, exposures, predictors, potential confounders, and effect modifiers. Give diagnostic criteria, if applicable **DONE – See Methods section**	8-11
Data sources/ measurement	8*	For each variable of interest, give sources of data and details of methods of assessment (measurement). Describe comparability of assessment methods if there is more than one group **DONE – See Methods section**	*10-11*
Bias	9	Describe any efforts to address potential sources of bias **N/A**	
Study size	10	Explain how the study size was arrived at **DONE – See Methods section**	8-11
Quantitative variables	11	Explain how quantitative variables were handled in the analyses. If applicable, describe which groupings were chosen and why **DONE – See Methods section**	10-11
Statistical methods	12	*(a)* Describe all statistical methods, including those used to control for confounding **DONE – See Methods section**	10-11
		(b) Describe any methods used to examine subgroups and interactions **DONE – See Methods section**	10-11
		(c) Explain how missing data were addressed **N/A**	-

For peer review only - http://bmjopen.bmj.com/site/about/guidelines.xhtml

		(d) If applicable, describe analytical methods taking account of sampling strategy **N/A**	-
		(e) Describe any sensitivity analyses **DONE – See Methods and Analysis section**	11
**Results**			
Participants	13*	(a) Report numbers of individuals at each stage of study—eg numbers potentially eligible, examined for eligibility, confirmed eligible, included in the study, completing follow-up, and analysed **DONE – See Results section/Figure 2**	12-13
		(b) Give reasons for non-participation at each stage **DONE – See Results section**	12-13
		(c) Consider use of a flow diagram **DONE – See Results section/ Figure 2**	12-13
Descriptive data	14*	(a) Give characteristics of study participants (eg demographic, clinical, social) and information on exposures and potential confounders **DONE – See Results section**	13-15
		(b) Indicate number of participants with missing data for each variable of interest **DONE – See Results section/Figure 2**	12-13
Outcome data	15*	Report numbers of outcome events or summary measures **DONE – See Results section**	13-23
Main results	16	*(a)* Give unadjusted estimates and, if applicable, confounder-adjusted estimates and their precision (eg, 95% confidence interval). Make clear which confounders were adjusted for and why they were included **DONE – See Results section**	13-23
		(b) Report category boundaries when continuous variables were categorized **DONE – See Results section**	13-23
		(c) If relevant, consider translating estimates of relative risk into absolute risk for a meaningful time period **N/A**	-
Other analyses	17	Report other analyses done—eg analyses of subgroups and interactions, and sensitivity analyses **DONE – See Results section**	20-21
**Discussion**			
Key results	18	Summarise key results with reference to study objectives **DONE – see Discussion section**	23-26
Limitations	19	Discuss limitations of the study, taking into account sources of potential bias or imprecision. Discuss both direction and magnitude of any potential bias **DONE – see Discussion section**	26-27

For peer review only - http://bmjopen.bmj.com/site/about/guidelines.xhtml

Interpretation	20	Give a cautious overall interpretation of results considering objectives, limitations, multiplicity of analyses, results from similar studies, and other relevant evidence<br>**DONE – see Discussion section**	23-26
Generalisability	21	Discuss the generalisability (external validity) of the study results<br>**DONE – see Discussion section**	23-27
**Other information**			
Funding	22	Give the source of funding and the role of the funders for the present study and, if applicable, for the original study on which the present article is based<br>**DONE – see Funding section**	29

*Give information separately for exposed and unexposed groups.

Note: An Explanation and Elaboration article discusses each checklist item and gives methodological background and published examples of transparent reporting. The STROBE checklist is best used in conjunction with this article (freely available on the Web sites of PLoS Medicine at http://www.plosmedicine.org/, Annals of Internal Medicine at http://www.annals.org/, and Epidemiology at http://www.epidem.com/). Information on the STROBE Initiative is available at www.strobe-statement.org.

For peer review only

BMJ Open

Representativeness of a mobile phone-based coverage evaluation survey following mass drug administration for soil-transmitted helminths: a comparison of participation between two cross-sectional surveys

Journal:	*BMJ Open*
Manuscript ID	bmjopen-2022-070077.R2
Article Type:	Original research
Date Submitted by the Author:	27-Sep-2023

Complete List of Authors:	Ramesh, Rohan; Christian Medical College Vellore, The Wellcome Trust Research Laboratory, Division of Gastrointestinal Sciences<br>Oswald, William; London School of Hygiene & Tropical Medicine, Department of Disease Control<br>Israel, Gideon ; Christian Medical College Vellore, The Wellcome Trust Research Laboratory, Division of Gastrointestinal Sciences<br>Aruldas, Kumudha; Christian Medical College Vellore, The Wellcome Trust Research Laboratory, Division of Gastrointestinal Sciences<br>Galagan, Sean ; University of Washington, Department of Global Health<br>Legge, Hugo; London School of Hygiene & Tropical Medicine, Department of Disease Control<br>PUTHUPALAYAM KALIAPPAN, SARAVANAKUMAR; Christian Medical College Vellore, The Wellcome Trust Research Laboratory, Division of Gastrointestinal Sciences<br>Walson, Judd; University of Washington, Departments of Global Health, Medicine, Pediatrics and Epidemiology<br>Halliday, Katherine; London School of Hygiene and Tropical Medicine Faculty of Infectious and Tropical Diseases, Department of Disease Control<br>Ajjampur, Sitara; Christian Medical College Vellore, The Wellcome Trust Research Laboratory, Division of Gastrointestinal Sciences

**Primary Subject Heading**:	Public health
Secondary Subject Heading:	Research methods, Qualitative research, Global health
Keywords:	Public health < INFECTIOUS DISEASES, Tropical medicine < INFECTIOUS DISEASES, QUALITATIVE RESEARCH

SCHOLARONE™
Manuscripts

I, the Submitting Author has the right to grant and does grant on behalf of all authors of the Work (as defined in the below author licence), an exclusive licence and/or a non-exclusive licence for contributions from authors who are: i) UK Crown employees; ii) where BMJ has agreed a CC-BY licence shall apply, and/or iii) in accordance with the terms applicable for US Federal Government officers or employees acting as part of their official duties; on a worldwide, perpetual, irrevocable, royalty-free basis to BMJ Publishing Group Ltd ("BMJ") its licensees and where the relevant Journal is co-owned by BMJ to the co-owners of the Journal, to publish the Work in this journal and any other BMJ products and to exploit all rights, as set out in our licence.

The Submitting Author accepts and understands that any supply made under these terms is made by BMJ to the Submitting Author unless you are acting as an employee on behalf of your employer or a postgraduate student of an affiliated institution which is paying any applicable article publishing charge ("APC") for Open Access articles. Where the Submitting Author wishes to make the Work available on an Open Access basis (and intends to pay the relevant APC), the terms of reuse of such Open Access shall be governed by a Creative Commons licence – details of these licences and which Creative Commons licence will apply to this Work are set out in our licence referred to above.

Other than as permitted in any relevant BMJ Author's Self Archiving Policies, I confirm this Work has not been accepted for publication elsewhere, is not being considered for publication elsewhere and does not duplicate material already published. I confirm all authors consent to publication of this Work and authorise the granting of this licence.

For peer review only

Representativeness of a mobile phone-based coverage evaluation survey following mass drug administration for soil-transmitted helminths: a comparison of participation between two cross-sectional surveys

Authors

Rohan Michael Ramesh*: The Wellcome Trust Research Laboratory, Division of Gastrointestinal Sciences, Christian Medical College, Vellore, Tamil Nadu, India

William Edward Oswald*: Department of Disease Control, Faculty of Infectious and Tropical Diseases, London School of Hygiene & Tropical Medicine, London, United Kingdom; Global Health Division, International Development Group, RTI International, Research Triangle Park, NC, USA.

Gideon John Israel: The Wellcome Trust Research Laboratory, Division of Gastrointestinal Sciences, Christian Medical College, Vellore, Tamil Nadu, India

Kumudha Aruldas: The Wellcome Trust Research Laboratory, Division of Gastrointestinal Sciences, Christian Medical College, Vellore, Tamil Nadu, India

Sean Galagan: Department of Global Health, University of Washington, Seattle, Washington, United States of America

Hugo Legge: Department of Disease Control, Faculty of Infectious and Tropical Diseases, London School of Hygiene & Tropical Medicine, London, United Kingdom

Saravanakumar Puthupalayam Kaliappan: The Wellcome Trust Research Laboratory, Division of Gastrointestinal Sciences, Christian Medical College, Vellore, Tamil Nadu, India

For peer review only

Judd L Walson: Departments of Global Health, Medicine, Pediatrics and Epidemiology, University of Washington, Seattle, Washington, United States of America

Katherine Elizabeth Halliday: Department of Disease Control, Faculty of Infectious and Tropical Diseases, London School of Hygiene & Tropical Medicine, London, United Kingdom

Sitara S.R. Ajjampur: The Wellcome Trust Research Laboratory, Division of Gastrointestinal Sciences, Christian Medical College, Vellore, Tamil Nadu, India

* These authors contributed equally to the work.

Correspondence to:

Sitara S.R. Ajjampur, The Wellcome Trust Research Laboratory, Division of Gastrointestinal Sciences, Christian Medical College, Vellore, Tamil Nadu, 632002, India

Email: sitararao@cmcvellore.ac.in

For peer review only

Keywords

Phone-based survey, coverage evaluation survey, in-person survey, low- and -middle-income countries, representativeness, non-response, participation, India

Word Count: 4049

ABSTRACT

Objectives: With increasing mobile phone subscriptions, phone-based surveys are gaining popularity with public health programs. Despite advantages, systematic exclusion of participants may limit representativeness. Similar to control programs for neglected tropical diseases, the DeWorm3 trial of biannual community-wide mass drug administration (MDA) for elimination of soil-transmitted helminth (STH) infection used in-person coverage evaluation surveys to measure the proportion of the at-risk population treated during MDA. Due to lockdown during the COVID-19 pandemic, a phone-based coverage evaluation survey was necessary, providing an opportunity for the current study to compare representativeness and implementation (including non-response) of these two survey modes.

Design: Comparison of two cross-sectional surveys.

Setting: The DeWorm3 trial site in Tamil Nadu, India, includes Timiri, a rural sub-site, and Jawadhu hills, a hilly, hard-to-reach sub-site inhabited predominantly by a tribal population.

Participants: In the phone-based and in-person coverage evaluation surveys, all individuals residing in 2000 randomly selected households (50 in each of the 40 trial clusters) were eligible to participate. Here we characterise household participation.

Results: Of 2000 households, 1780 (89.0%) participated during the in-person survey. Of 2000 households selected for the phone survey, 346 (17.3%) could not be contacted as they had not provided a telephone number during the census and 1144 (57.2%) participated. Smaller households, households with lower socioeconomic status, and those with older, female or less educated household-heads were under-represented in the phone-based survey compared to censused households. Regression analysis revealed non-response in the phone-based survey was higher

For peer review only

among households from the poorest socioeconomic quintile (Prevalence Ratio [PR] 2.3, 95% Confidence Interval [CI] 2.0–2.7) and lower when heads of households had completed secondary school or higher education (PR 0.7, 95% CI:0.6–0.8).

Conclusions: Our findings suggest phone-based surveys underrepresent households likely to be at higher risk of NTDs and in-person surveys are more appropriate for measuring MDA coverage within programmatic settings.

Trial registration: ClinicalTrials.gov, NCT03014167.

Strengths and limitations of this study

- A detailed and up-to-date census in the study population allowed us to robustly assess representativeness of both survey modes.
- The two surveys were conducted six months apart using the same questionnaire, allowing us to compare participation confidently.
- Generalizability may be limited as the study was conducted in a setting with high mobile phone usage and network coverage, and during the COVID-19 pandemic and resulting lockdown.

For peer review only

INTRODUCTION

Telephone-based surveys have been widely used in public health programs and research, for purposes such as determining immunisation coverage of children under three years, conducting surveillance for risk factors related to leading causes of death, assessing mental health, and self-reporting alcohol consumption [1-5]. This survey mode has become popular with increasing mobile phone subscription rates in low- and -middle-income countries, where cell phone coverage in some regions compares with that in high-income countries [6,7]. Cost-effectiveness, time efficiency, increased reach, and willingness to share sensitive information are some advantages of phone-based surveys [8,9]. Disadvantages of phone-based surveys include the inability to collect visual data, lower response rates in some communities, and exclusion of participants without access to phones [9,10]. Mobile phone ownership is not universal, and the practical appeal of phone-based surveys may come at a cost by systematically excluding segments of the population, raising questions about the representativeness of the estimates they yield [8,11–13]. India's average tele-density, or the number of telephone connections (wireless and wired) for every hundred individuals living within an area, is 88.5, with a slight disparity between most rural and urban areas [14,15]. Tamil Nadu, in southern India, with a population of 72 million, is the fifth most tele-dense state of the 28 states in India with 108.5 telephone connections per 100 people, which is higher than the Indian average, with high mobile phone penetration even in rural households [14,15].

Neglected tropical diseases (NTDs) predominantly affect impoverished, rural communities, and preventive chemotherapy (PC) through mass drug administration

For peer review only

(MDA) is one of the key public health interventions implemented to control soil-transmitted helminth (STH) infections, lymphatic filariasis (LF), trachoma, schistosomiasis, and onchocerciasis [16]. The World Health Organization (WHO) refers to coverage evaluation surveys as population-based surveys that offer a simple and effective method to accurately assess programme performance and provide precise estimates of PC coverage, or the proportion of individuals who swallowed the medicine or combination of medicines, for targeted NTDs [17,18]. These surveys are a valuable tool for evaluating the performance of NTD control programs. For programs targeting lymphatic filariasis, trachoma and onchocerciasis, community-wide MDA (cMDA) coverage is assessed by household surveys using a standardised WHO tool [17,19].

DeWorm3 is a trial of cMDA for interrupting transmission of STH, during which household coverage evaluation surveys were conducted after each of six cMDA rounds in the years 2018–2020 [20,21]. In April 2020, however, during the nationwide lockdown imposed by the Government of India as a control measure during the COVID-19 pandemic, a phone-based survey was conducted instead of an in-person survey as in the previous four rounds. Leveraging this change in the trial protocol, necessitated by the COVID-19 pandemic, we conducted this analysis to contrast the representativeness, or household participation, and implementation of a phone-based survey with a previous in-person survey conducted six months earlier.

For peer review only

METHODS

DeWorm3 trial

DeWorm3 is a cluster-randomised, controlled, community-based intervention trial that aims to determine the feasibility of interrupting STH transmission in Benin, India, and Malawi [21,22]. In brief, 40 clusters at each study site were randomised to control clusters, to receive the standard of care, which in India is twice-yearly targeted deworming of children attending schools and pre-schools between 1 – 19 years of age during National Deworming Days (NDDs) [23], or intervention clusters, to receive twice-yearly cMDA (delivered door-to-door) for three years (between 2018 and 2020) (**Figure 1**) [24]. At the start of each of the three years, an annual census was conducted to obtain accurate population data at the individual and household level and optimise coverage estimates during cMDAs. In all intervention clusters, cMDA was implemented simultaneously the day after NDD, followed by a mop-up revisiting previously unavailable households. Following each of the six rounds of cMDA, coverage evaluation surveys were conducted in the intervention and control clusters within a week of cMDA. Surveys in control clusters were intended to measure the coverage of treatment provided through the NDD program. All census and coverage evaluation surveys (in-person and phone-based) were collected using the same electronic data collection forms programmed into the SurveyCTO mobile application (Dobility, Inc; Cambridge, Massachusetts and Washington, DC) run on encrypted Android smartphones. The survey forms in this application were embedded with logic checks, skip logic, numerical constraints and pre-loaded with location and demographic information to reduce errors while entering data.

Study setting

The DeWorm3 trial site in India includes a sub-site in Timiri, a rural area in Ranipet district (formerly a part of Vellore district), and another sub-site in Jawadhu hills in Tiruvannamalai district, a hilly, hard-to-reach area inhabited predominantly by tribal groups. Both sub-sites are located in the southern state of Tamil Nadu. The study covers an annually enumerated population of nearly 141,000, residing in approximately 37,000 households, with a total area of 477 km^2 [20]. Males and females are equally represented, and children below 15 years of age constitute 23% of the population. Nearly a third of the adults received no formal education, with 20% and 90% of the population engaged in agricultural activities in Timiri and Jawadhu hills, respectively [21]. More details on India's study site and population have been published previously [20].

Coverage evaluation surveys

For the coverage evaluation surveys, 50 households were randomly selected to participate from each of the 40 clusters (n=2000). Questions in the survey aligned with the WHO-endorsed coverage evaluation survey for use by national programs, including individual-level questions on receipt and swallowing of the drug and reasons for not taking treatment, if applicable (**Supplementary file**) [18]. The in-person coverage evaluation survey in 2019 was conducted from 27th to 31st August, based on a household sampling frame derived from the previous annual census (25th October 2018 to 2nd February 2019) **(Figure 1).** All households had to be visited up to three times before they were considered unavailable, and the reasons were recorded. Sampled households that were not located or unavailable were substituted as necessary with households from two randomly-selected staged replacement lists of 20 households per cluster. First, the respondent answered the household-level

For peer review only - http://bmjopen.bmj.com/site/about/guidelines.xhtml

questions, and then each member present was interviewed. Proxy responses from household members were accepted for children under five years of age and household members absent on the second or third visit.

The phone-based coverage evaluation survey was conducted from 31st March to 10th April 2020 with a household sampling frame derived from the previous annual census (19th October 2019 to 31st January 2020) (**Figure 1**). All households were eligible for selection, regardless of whether they had provided a phone number during the census or not. Households without a telephone number, who did not consent, or whose number was not reachable (two attempts made four hours apart were made on the first day and the third and fourth attempts made the next day) were also substituted with households from replacement lists. The interviewers were trained to administer the coverage evaluation survey over the phone while recording responses on the SurveyCTO mobile application on another device. The next available adult was interviewed using the same phone number if the head of the household (preferred respondent) was unavailable for three attempts. Thereafter, other household members were also interviewed over the phone during the same call. The primary respondent was allowed to provide proxy responses for children under five years and household members absent on any attempt.

Data analysis

To assess representativeness, we compared the characteristics of households that participated in each of the coverage evaluation surveys with those of households in the previous census not randomly-selected for the coverage evaluation survey and identified factors associated with household non-response during both of the coverage

evaluation surveys. To achieve the sample size for each coverage evaluation survey, additional lists of households were randomly selected and released to the survey teams to compensate for unavailable households. For the current study contrasting representativeness, we included only the initial 2000 households sampled for each survey, and restricted analyses to households with complete data. We compared characteristics of households participating in each of the coverage evaluation surveys with households in the most recent previous annual census considering age, sex, reported education, and marital status of household head; household religion and caste; and period of time in residence and house type. Principal component analysis (PCA) using household assets was used to arrive at a composite wealth index and divided into five socioeconomic status (SES) quintiles, as described previously [20]. Participants who were currently married, separated, widowed or divorced were considered ever married. Chi-squared tests were used to compare distributions of household characteristics between census and surveyed households.

For each coverage evaluation survey, we identified factors associated with household non-response by fitting modified Poisson regression models with robust errors to adjust for clustering [25]. For this analysis, a household was considered to have a response if at least one household member was present and agreed to respond to the survey. Households not meeting this criterion for any reason were considered a non-response. We estimated the prevalence ratio (PR) between each candidate predictor variable and non-response. We then used a best-subset selection approach, modelling all possible combinations of candidate predictor variables hypothesised to influence a household's participation and selecting the final model with the smallest Bayesian Information Criterion [26]. All candidate models included sub-site, and

For peer review only - http://bmjopen.bmj.com/site/about/guidelines.xhtml

household caste was excluded from the multivariable model selection because of collinearity with sub-site. In a sensitivity analysis, we examined the inclusion of mobile phone ownership and phone number availability as additional candidate predictors to determine factors associated with household non-response. As a *post hoc* analysis, we also examined the association between households who reported owning a mobile phone and phone number availability.

In addition, we contrasted the implementation of each of the coverage evaluation surveys by comparing the number of attempts to contact (visits or calls), time of the day the visits/calls were made, duration per visit or call, number of individuals interviewed per attempt, proportion of household residents interviewed per attempt, total duration to complete the household's survey, and number of visits or calls to complete the household survey. We used a nonparametric K-sample test of medians for continuous measures, a Pearson's chi-squared test for categorical measures, and compared mean total duration weighted by household size using a Wald test. Statistical significance for all tests was set at p-value<0.05. The data were managed and analysed using STATA 16.1 software (StataCorp, TX, USA).

Patient and public involvement

None.

RESULTS

Households available for participation in coverage evaluation surveys

For peer review only

During the 2019 in-person coverage evaluation survey, of the 2000 households sampled, 1780 (89.0%) participated, and during the 2020 phone-based coverage evaluation survey, 1144 (57.2%) of 2000 sampled households participated. Data were incomplete for 24 and 17 households of those sampled for the in-person and phone-based surveys, respectively. The number of households not participating due to various reasons during the in-person and phone-based surveys is illustrated in **Figure 2**. During the in-person survey, members were unavailable in 203 (10.1%) households even after three visits. During the phone-based survey, 346 (17.3%) households could not be contacted as phone numbers were unavailable. Further, 505 (25.2%) households were unavailable after three or more phone calls – these included 61 households where the phone call was not picked up, 269 for whom the 'number not reachable' message was received from the phone service provider as the phone was outside an area of connection, 12 primary respondents informed the interviewers that they were 'out of town' and not present during the cMDA and as a result were unable to answer on behalf of their family members, and 163 were recorded as 'wrong number' as the call was attended by persons not associated with the surveyed household.

Representativeness of coverage evaluation surveys

No differences were observed when the characteristics of households and heads of households participating in the in-person coverage evaluation survey were compared to censused households that were not sampled **(Table 1).** In the phone-based survey, however, participating households in the highest SES quintile were overrepresented (27.5%) compared to non-sampled households (19.9%), with the inverse observed in the poorest quintile (p<0.01). Larger households with five or more household members

For peer review only

were also overrepresented in the phone-based survey (39.8%) compared to non-sampled households (34.0%, p<0.01).

Table 1. Comparison of household characteristics between censused and participating households for in-person and phone-based coverage surveys in Vellore, India, 2018-2020

	Census households (2018-19) N=35663 n(%)	In-person coverage survey N=1765 n(%)	P value†	Census households (2019-20) N=35900 n(%)	Phone-based coverage survey N=1134 n(%)	P value†
Site details						
**Study arm**						
Control	17420 (48.8)	880 (49.9)	0.41	17431 (48.6)	564 (49.7)	0.43
Intervention	18243 (51.2)	885 (50.1)		18469 (51.4)	570 (50.3)	
**Study sub-site**						
Timiri	27638 (77.5)	1394 (79.0)	0.14	27959 (77.9)	892 (78.7)	0.53
Jawadhu Hills	8025 (22.5)	371 (21.0)		7941 (22.1)	242 (21.3)	
Household characteristics						
**Religion**						
Other	1086 (3.0)	45 (2.5)	0.24	1065 (3.0)	41 (3.6)	0.21
Hindu	34577 (97.0)	1720 (97.5)		34835 (97.0)	1093 (96.4)	
**Caste**						
Higher caste	404 (1.1)	17 (1.0)	0.66	410 (1.1)	14 (1.2)	0.55
Backward caste	10667 (29.9)	536 (30.4)		10727 (29.9)	364 (32.1)	
Most backward caste	8533 (23.9)	440 (24.9)		8720 (24.3)	274 (24.2)	
Scheduled caste	7960 (22.3)	393 (22.3)		8018 (22.3)	241 (21.3)	
Scheduled tribes	8099 (22.7)	379 (21.5)		8025 (22.4)	241 (21.3)	
**House type***						
Concrete	18525 (51.9)	896 (50.8)	0.78	19001 (52.9)	637 (56.2)	0.13
Mixed	3975 (11.1)	203 (11.5)		3957 (11.0)	120 (10.6)	
Government donated/funded house	3380 (9.5)	167 (9.5)		3318 (9.2)	105 (9.3)	
Thatched	9783 (27.4)	499 (28.3)		9624 (26.8)	272 (24.0)	
**Socio-economic quintiles**						
Least poor quintile	7120 (20.0)	343 (19.4)	0.58	7151 (19.9)	312 (27.5)	**<0.01**
4th quintile	6965 (19.5)	321 (18.2)		7179 (20.0)	229 (20.2)	
3th quintile	7273 (20.4)	375 (21.2)		7134 (19.9)	221 (19.5)	
2nd quintile	7118 (20.0)	363 (20.6)		7212 (20.1)	195 (17.2)	
Poorest quintile	7187 (20.2)	363 (20.6)		7224 (20.1)	177 (15.6)	

For peer review only

**Large household (family size)**						
No (<= 4 members)	24027 (67.4)	1180 (66.9)	0.65	23679 (66.0)	683 (60.2)	**<0.01**
Yes (>= 5 members)	11636 (32.6)	585 (33.1)		12221 (34.0)	451 (39.8)	
Head of the household characteristics						
**Age**						
18-30 years	2143 (6.0)	92 (5.2)	0.68	1516 (4.2)	53 (4.7)	**<0.01**
31-40 years	7071 (19.8)	348 (19.7)		6842 (19.1)	246 (21.7)	
41-50 years	9103 (25.5)	451 (25.6)		9192 (25.6)	331 (29.2)	
51-60 years	7827 (21.9)	402 (22.8)		8417 (23.4)	247 (21.8)	
>61 years	9519 (26.7)	472 (26.7)		9933 (27.7)	257 (22.7)	
**Sex**						
Male	28260 (79.2)	1412 (80.0)	0.44	29198 (81.3)	964 (85.0)	**<0.01**
Female	7403 (20.8)	353 (20.0)		6702 (18.7)	170 (15.0)	
**Education level**						
No education	12047 (33.8)	588 (33.3)	0.55	10994 (30.6)	270 (23.8)	**<0.01**
Any primary	7604 (21.3)	379 (21.5)		8161 (22.7)	256 (22.6)	
Any middle	6210 (17.4)	329 (18.6)		6419 (17.9)	202 (17.8)	
Any secondary or higher	9802 (27.5)	469 (26.6)		10326 (28.8)	406 (35.8)	
**Marriage status**						
Never married	6778 (19.0)	324 (18.4)	0.50	7446 (20.7)	168 (14.8)	**<0.01**
Ever married	28885 (81.0)	1441 (81.6)		28454 (79.3)	966 (85.2)	
**Owns mobile phone**						
No	5243 (14.7)	268 (15.2)	0.75	5430 (15.1)	43 (3.8)	**<0.01**
Yes	27972 (78.4)	1382 (78.3)		30470 (84.9)	1091 (96.2)	
Data not available	2448 (6.9)	115 (6.5)				

*Concrete - concrete walls and roof; Mixed - concrete walls and tiled roof; Government donated/funded house - prebuilt government houses or houses funded through schemes for economically and socially marginalised groups; Thatched - thatched walls and roof.
†Pearson's chi-squared test

When their characteristics were compared, heads of households participating in the phone-based survey were most commonly aged between 41–50 years (29.2%) in contrast to those in the non-sampled households who were mainly older than 60 years (27.7%, p<0.01). Similarly, households with female heads were under-represented in the phone-based survey (15.0%) compared to non-sampled households (18.7%, p<0.01). Households where the heads reported no formal education were also

For peer review only - http://bmjopen.bmj.com/site/about/guidelines.xhtml

underrepresented in the phone-based survey (23.8%) compared to non-sampled households (30.6%, p<0.01). Households headed by individuals who were never married were also significantly underrepresented in the phone-based survey (14.8%) compared to non-sampled households (20.7%, p<0.01). Reported ownership of mobile phones was higher (96.2%) compared to non-sampled households (84.9%, p<0.01) in the phone-based coverage evaluation survey. **(Table 1).**

Factors associated with household participation

Of the 1983 households with complete data sampled for the phone-based survey, 849 (42.8%) were considered non-responders. The selected modified Poisson regression model included SES and the education of the head of the household. Non-response increased with greater poverty, and households in the poorest quintile were more than twice as likely to have not responded as households in the least poor quintile (PR: 2.3, 95% CI:2.0–2.7), adjusting for site and education of the household head. Non-response was lower in households with heads who reported any education compared to those who reported no education (p<0.01) in the phone-based survey **(Table 2)**, adjusting for site and SES**.** Of the 1976 households sampled for the in-person survey and with complete data, 211 (10.7%) were non-responders. Similar to the phone-based survey, the unadjusted estimate for non-response in the in-person survey was lower in the Jawadhu Hills sub-site compared to the Timiri sub-site (PR: 0.5, 95% CI:0.3–0.8), and age was selected (p<0.01) once sub-site was included **(Table 3).**

Table 2. Factors associated with non-response in a phone-based coverage evaluation survey in Vellore, India, 2020

	Households†	Non-response‡	Univariate	Multivariable

For peer review only - http://bmjopen.bmj.com/site/about/guidelines.xhtml

	N=1983 (100) n (%)	n=849 (42.8) n (%)	PR (95% CI)	P value	PR (95% CI)	P value
Site details						
**Proportion of households in each arm**						
Control	993 (50.1)	429 (43.2)	REF	0.78		
Intervention	990 (49.9)	420 (42.4)	1.0 (0.9, 1.1)			
**Proportion of households per site**						
Timiri	1586 (80.0)	694 (43.8)	REF	0.45	REF	**<0.01**
Jawadhu Hills	397 (20.0)	155 (39.0)	0.9 (0.7, 1.2)		0.6 (0.4, 0.8)	
Household characteristics						
**Religion**						
Other	68 (3.4)	27 (39.7)	REF	0.67		
Hindu	1915 (96.6)	822 (42.9)	1.1 (0.8, 1.5)			
**Caste**						
Higher caste	20 (1.0)	6 (30.0)	REF	0.50	--	--
Backward caste	635 (32.0)	271 (42.7)	1.4 (0.8, 2.6)		--	--
Most backward caste	487 (24.6)	213 (43.7)	1.5 (0.8, 2.7)		--	--
Scheduled caste	439 (22.1)	198 (45.1)	1.5 (0.9, 2.6)		--	--
Scheduled tribes	402 (20.3)	161 (40.0)	1.3 (0.7, 2.5)		--	--
**House type**§						
Concrete	1054 (53.2)	417 (39.6)	REF	**0.01**		
Mixed	233 (11.7)	113 (48.5)	1.2 (1.1, 1.4)			
Government funded/ donated house	200 (10.1)	95 (47.5)	1.2 (1.0, 1.5)			
Thatched	496 (25.0)	224 (45.2)	1.1 (1.0, 1.3)			
**Socio-economic quintiles**						
Least poor quintile	422 (21.3)	110 (26.1)	REF	**<0.01**	REF	**<0.01**
4th quintile	390 (19.7)	161 (41.3)	1.6 (1.3, 1.9)		1.5 (1.2, 1.8)	
3th quintile	406 (20.5)	185 (45.6)	1.7 (1.4, 2.1)		1.6 (1.4, 2.0)	
2nd quintile	386 (19.5)	191 (49.5)	1.9 (1.5, 2.3)		1.9 (1.6, 2.3)	
Poorest quintile	379 (19.1)	202 (53.3)	2.0 (1.7, 2.5)		2.3 (2.0, 2.7)	
**Large household (family size)**						
No (<= 4 members)	1286 (64.9)	603 (46.9)	REF	**<0.01**		
Yes (>= 5 members)	697 (35.1)	246 (35.3)	0.8 (0.7, 0.8)			

For peer review only

Head of the household characteristics						
**Age**						
18-30 years	84 (4.2)	31 (36.9)	REF	**<0.01**		
31-40 years	388 (19.6)	142 (36.6)	1.0 (0.8, 1.3)			
41-50 years	516 (26.0)	185 (35.9)	1.0 (0.8, 1.2)			
51-60 years	431 (21.7)	184 (42.7)	1.2 (0.9, 1.5)			
>61 years	564 (28.4)	307 (54.4)	1.5 (1.1, 2.0)			
**Sex**						
Male	1602 (80.8)	638 (39.8)	REF	**<0.01**		
Female	381 (19.2)	211 (55.4)	1.4 (1.2, 1.6)			
**Education level**						
No education	587 (29.6)	317 (54.0)	REF	**<0.01**	REF	**<0.01**
Any primary	452 (22.8)	196 (43.4)	0.8 (0.7, 0.9)		0.8 (0.7, 0.9)	
Any middle	343 (17.3)	141 (41.1)	0.8 (0.6, 0.9)		0.8 (0.6, 0.9)	
Any secondary or higher	601 (30.3)	195 (32.4)	0.6 (0.5, 0.7)		0.7 (0.6, 0.8)	
**Marriage status**						
Never married	397 (20.0)	229 (57.7)	REF	**<0.01**		
Ever married	1586 (80.0)	620 (39.1)	0.7 (0.6, 0.8)			

Acronyms: PR - Prevalence Ratio; CI - Confidence Interval

†Number (N) of sampled households with complete covariate data used to estimate prevalence of non-response in each category.

‡Non-response - no household members available for interview, household refused to participate or unable to consent, or the household was not attempted.

§Concrete - concrete walls and roof; Mixed - concrete walls and tiled roof; Government donated/funded house - prebuilt government houses or houses funded through schemes for economically and socially marginalised groups; Thatched - thatched walls and roof.

For peer review only - http://bmjopen.bmj.com/site/about/guidelines.xhtml

Table 3. Factors associated with non-response in an in-person coverage evaluation survey in Vellore, India, 2019

	In-person coverage survey					
	**Households†**	**Non-response‡**	**Univariate**		**Multivariable**	
	**N=1976 (100) n (%)**	**n=211 (10.7) n (%)**	**PR (95% CI)**	**P value**	**PR (95% CI)**	**P value**
Site details						
**Proportion of households in each arm**						
Control	990 (50.1)	110 (11.1)	REF	0.65		
Intervention	986 (49.9)	101 (10.2)	0.9 (0.7, 1.3)			
**Proportion of households per site**						
Timiri	1581 (80.0)	187 (11.8)	REF	**<0.01**	REF	**<0.01**
Jawadhu Hills	395 (20.0)	24 (6.1)	0.5 (0.3, 0.8)		0.5 (0.3, 0.8)	
Household characteristics						
**Religion**						
Other	58 (2.9)	13 (22.4)	REF	**<0.01**		
Hindu	1918 (97.1)	198 (10.3)	0.5 (0.3, 0.7)			
**Caste**						
Higher caste	18 (0.9)	1 (5.6)	REF	0.18	--	--
Backward caste	609 (30.8)	73 (12.0)	2.2 (0.3, 16.0)		--	--
Most backward caste	502 (25.4)	62 (12.4)	2.2 (0.3, 16.2)		--	--
Scheduled caste	439 (22.2)	46 (10.5)	1.9 (0.2, 14.4)		--	--
Scheduled tribes	408 (20.6)	29 (7.1)	1.3 (0.2, 9.8)		--	--
**House type§**						
Concrete	1019 (51.6)	123 (12.1)	REF	**0.02**		
Mixed	229 (11.6)	26 (11.4)	0.9 (0.6, 1.4)			
Government funded/ donated house	191 (9.7)	24 (12.6)	1.0 (0.7, 1.5)			
Thatched	537 (27.2)	38 (7.1)	0.6 (0.4, 0.9)			
**Socio-economic quintiles**						
Least poor quintile	391 (19.8)	48 (12.3)	REF	**0.02**		
4th quintile	360 (18.2)	39 (10.8)	0.9 (0.6, 1.3)			
3th quintile	429 (21.7)	54 (12.6)	1.0 (0.7, 1.6)			
2nd quintile	410 (20.7)	47 (11.5)	0.9 (0.6, 1.5)			
Poorest quintile	386 (19.5)	23 (6.0)	0.5 (0.3, 0.8)			
**Large household (family size)**						

For peer review only

For peer review only - http://bmjopen.bmj.com/site/about/guidelines.xhtml

No ≤ 4 members)	1339 (67.8)	159 (11.9)	REF	**0.02**		
Yes (≥ 5 members)	637 (32.2)	52 (8.2)	0.7 (0.5, 0.9)			
Head of the household characteristics						
**Age**						
18-30 years	108 (5.5)	16 (14.8)	REF	**0.03**	REF	**<0.01**
31-40 years	403 (20.4)	55 (13.6)	0.9 (0.6, 1.5)		0.8 (0.5, 1.3)	
41-50 years	489 (24.7)	38 (7.8)	0.5 (0.3, 0.9)		0.4 (0.3, 0.7)	
51-60 years	455 (23.0)	53 (11.6)	0.8 (0.5, 1.3)		0.6 (0.4, 1.0)	
>61 years	521 (26.4)	49 (9.4)	0.6 (0.3, 1.2)		0.5 (0.3, 0.9)	
**Sex**						
Male	1567 (79.3)	155 (9.9)	REF	**0.02**		
Female	409 (20.7)	56 (13.7)	1.4 (1.1, 1.8)			
**Education level**						
No education	655 (33.1)	67 (10.2)	REF	0.36		
Any primary	427 (21.6)	48 (11.2)	1.1 (0.8, 1.5)			
Any middle	360 (18.2)	31 (8.6)	0.8 (0.5, 1.4)			
Any secondary or higher	534 (27.0)	65 (12.2)	1.2 (0.9, 1.6)			
**Marriage status**						
Never married	377 (19.1)	53 (14.1)	REF	**0.01**		
Ever married	1599 (80.9)	158 (9.9)	0.7 (0.5, 0.9)			

Acronyms: PR - Prevalence Ratio; CI - Confidence Interval

†Number (N) of sampled households with complete covariate data used to estimate prevalence of non-response in each category.

‡Non-response - no household members available for interview, household refused to participate or unable to consent, or the household was not attempted.

§Concrete - concrete walls and roof; Mixed - concrete walls and tiled roof; Government donated/funded house - prebuilt government houses or houses funded through schemes for economically and socially marginalised groups; Thatched - thatched walls and roof.

As a sensitivity analysis, we examined mobile phone ownership and the availability of phone numbers as additional candidate predictors. The final selected model for non-response in the phone-based survey included mobile phone ownership and SES, but it no longer included head of household education. Including mobile phone ownership attenuated but did not entirely remove the association of SES with non-response (households from the poorest SES quintile: PR: 1.7, 95% CI:1.4–2.1). Mobile phone

For peer review only

ownership was strongly associated with non-response in the phone-based survey independent of SES and adjusting for sub-site, but not in the in-person survey, for which the results did not change with the inclusion of mobile phone ownership as a candidate predictor (**Supplementary Tables 1 & 2**). Using phone number availability in place of mobile phone ownership, the predictor was again selected and had a slightly stronger association with non-response in the phone-based survey model, but there was no change in results for the in-person survey (**Supplementary Tables 3 & 4**). Cross-tabulation of phone number availability among those who reported owning a mobile phone showed that 93.5% (1461/1563) of the phone numbers were available in the in-person survey and decreased slightly to 89.6% (1524/1700) in the phone-based survey **(Supplementary Table 5).**

In 2020, 5728 (15.1%) out of the 37,883 censused households reported not owning a mobile phone. Households in the poorest quintile were 19 times more likely not to own a mobile phone than households in the least poor quintile (PR:19.3, 95% CI:15.3–24.3). Non-ownership of phones was less likely in households with heads who were more educated compared to those who reported no education ($p<0.01$). (**Supplementary Table 6).**

Differences in the implementation of in-person and phone-based surveys

For the in-person survey, 2932 attempts were made to reach the 2000 households, during 2376 (81.0%) of which household respondents were located and available to participate. In the phone-based survey, 3262 attempts were made to reach the 2000 households, during which primary respondents were located and available to participate in 1166 (35.7%) attempts **(Table 4).** The phone-based surveys were most

For peer review only - http://bmjopen.bmj.com/site/about/guidelines.xhtml

often conducted after 18:00 hrs, in contrast to the in-person interviews conducted mainly between 10:01 to 18:00 hrs (p<0.01). The mean duration of each in-person survey visit was longer (3.5 min) than the mean time taken to complete a call during the phone-based survey (1.9 min, p<0.01). During the in-person survey, each visit interviewed a median of 2.0 individuals per household or 66.7 of residents compared to each call during the phone-based survey, when a median of 0 was interviewed per call (which includes all the calls where nobody was present/could be reached) (p<0.01).

Table 4. Comparison of implementation of in-person and phone-based coverage surveys in Vellore, India, 2019-2020

	**In-person coverage survey**	**Phone-based coverage survey**	**P value***
Discrete household visits or calls	n=2932 (100.0)	n=3262 (100.0)	
**Household status during visit or call**			
Not located	2 (0.1)	1 (0.0)	
Members deceased	7 (0.2)	0 (0.0)	
Members not present	547 (18.7)	2095 (64.2)	**<0.01**
Members present	2376 (81.0)	1166 (35.7)	
**Time of day**			
<10:00	149 (5.1)	83 (2.5)	**<0.01**
10:01-12:00	656 (22.4)	398 (12.2)	
12:01-14:00	832 (28.4)	785 (24.1)	
14:01-16:00	659 (22.5)	535 (16.4)	
16:01-18:00	542 (18.5)	457 (14.0)	
>18:00	94 (3.2)	1004 (30.8)	
**Mean duration per visit or call (min)**	3.5 (1.8 - 6.9)	1.9 (1.0 - 4.4)	**<0.01**
**Median number of resident interviews completed per visit or call**	2.0 (1.0 - 4.0)	0.0 (0.0 - 3.0)	**<0.01**
**Median proportion of resident interviews completed per visit or call (IQR)**	66.7 (25.0 - 100.0)†	0.0 (0.0 - 100.0)†	**<0.01**
Household survey completion	n=1765 (100.0)	n=1134 (100.0)	
**Total duration to complete household survey (min)**	6.3 (3.6 - 10.6)	6.5 (3.8 - 10.8)	0.47
**Weighted mean total duration (min) ‡**	9.3 (9.0 - 9.7)	8.8 (8.4 - 9.2)	**0.05**

For peer review only - http://bmjopen.bmj.com/site/about/guidelines.xhtml

**Visits or calls to complete household survey**			
1	981 (55.6)	780 (68.8)	**<0.01**
2	730 (41.4)	174 (15.3)	
3	51 (2.9)	61 (5.4)	
4+	3 (0.2)	119 (10.5)	

*Pearson's chi-squared/T-test as appropriate. For status analysis, not located and members deceased were excluded due to small cell counts.

†Median (Interquartile range)

‡Weighted according to the household size

For peer review only

Most in-person survey households were completed in 1 or 2 visits (97.0%), but by phone, households were mainly completed in the first attempt (68.8%). Considering the household size, the weighted total time to complete the household's survey was slightly longer during the in-person survey (9.3 min) than in the phone-based survey (8.8 min, p=0.05) **(Table 4).**

DISCUSSION

In this study, we compared representativeness and implementation of a phone-based survey with an in-person survey to measure treatment coverage following cMDA for STH as part of the DeWorm3 trial in rural communities in southern India. Household participation in surveys assessing the coverage of the STH program was higher when conducted in person than when done using a phone-based survey (89.0% versus 57.2%). When comparing the characteristics of participating households, larger households, households with higher socioeconomic status and those with younger, more educated, and male heads of household were over-represented in the phone-based survey. Adjusting for sub-site, non-response in the phone-based survey was higher among households from the poorest SES quintile and households with

uneducated household heads, while non-response in the in-person survey was associated with the age of the household head.

We observed that households with higher socioeconomic status were overrepresented in the phone-based survey. Studies conducted in other low- and -middle-income countries have demonstrated that households with higher socioeconomic status are more likely to own mobile phones [27,28]. Furthermore, the phone-based survey was implemented during the COVID-19 lockdown in India when income and employment were drastically reduced, potentially increasing the challenges faced by poorer households to recharge phone subscriptions [29,30]. Both issues could likely explain lower overall participation rates among the poorest households in the phone-based survey, who were approximately half as likely to respond as the least poor households, based on the non-response model. Larger households were overrepresented in the phone-based survey, potentially because the probability of someone in the family owning a phone may be higher in such households, but this characteristic was not selected during modelling. Similar to our findings related to SES, studies in Germany and the UK also reported an under-representation of socially disadvantaged and/or low-income participants in phone-based surveys [8,10].

We also noted that participation of households headed by individuals in certain demographic groups differed between in-person and phone-based surveys. The phone-based survey had a higher proportion of respondents from households with younger, more educated, and male heads of household compared to the censused population. The effect of education was also noted in our model revealing that non-response in the phone-based survey was lower in households with heads who

For peer review only

reported any education compared to those who reported no education. The effect of age on participation in phone-based surveys has been well documented in related health surveys from the United States, Ireland, UK and Brazil [8,12,13]. In terms of gender, low phone ownership among women residing in South Asia has been reported previously due to traditional gender norms, technical illiteracy in operating a phone, male dominance over phone usage, and economic reasons [31].

Of the 1983 sampled households with complete data in the phone-based survey, 1685 (85.0%) had provided a mobile phone number during the annual census. The availability of a phone number does not always result in a response, however, often due to poor network coverage, unrenewed subscriptions, or changed subscriptions, as reported in a study on cell phone ownership in Burkina Faso [32]. From the starting sample of 2000 households, nearly twice as many households participated in the in-person survey compared to the phone-based survey. If information on population characteristics is available beforehand, researchers conducting phone-based surveys could consider quota sampling, where participants are selected non-randomly according to a fixed quota or percentage of the population based on one or more characteristics, or post-stratification weight adjustment following data collection to ensure that all age groups and socio-economic strata are equally represented [10,33]. Both methods can be employed if non-response remains high following quota sampling.

Interviewers initially contacted participants on the phone during the same working hours as the in-person survey. However, during implementation, we observed two distinct periods (mid-day between 12:01 to 14:00 and after 18:00 hrs) of response in

For peer review only

the phone-based survey (data not shown) and adapted the time of the calls to this observed pattern. Phone calls can occur in unexpected instances, and the owner may not be in a suitable environment to answer, such as during working hours, which can impact response rates [34]. Although phone-based surveys allowed us to adapt interview timings, we recommend considering the local culture and work patterns of the target population during planning to ensure high response rates. As observed in our study, phone-based surveys provide the advantage of flexibility in terms of survey timing, enabling data collection beyond the typical working hours and daylight constraints associated with in-person surveys, particularly in the context of safety considerations in many countries.

The in-person survey took marginally longer to complete as it necessitated the interviewer to verify and interview all available household members individually. This observed difference in duration would increase if the travel time to the house for an in-person survey, particularly in hard-to-reach rural areas, is considered. Arguably, the phone-based survey was faster to complete since one participant could respond on behalf of those absent, explaining higher completion rates on the first attempt. However, the number of attempts to establish a successful contact was higher in the phone-based survey because phone calls often failed to connect, similar to phone-based surveys conducted in Ireland and the UK [8].

NTDs are more prevalent in socially and economically disadvantaged groups living in poor environmental conditions [35]. In this population, STH infections, particularly hookworm, are more prevalent in households belonging to poorer SES quintiles with less-educated household heads [20]. MDA programs are one of the key public health

For peer review only - http://bmjopen.bmj.com/site/about/guidelines.xhtml

interventions implemented to control or eliminate NTDs, including STH, and a coverage survey following an MDA program evaluates the performance of the intervention. Therefore, it is imperative that the population included in a coverage evaluation survey does not exclude the same groups at a higher risk of infection to avoid biasing estimates of treatment coverage. In this regard, the phone-based survey under-represents many of these groups. Our findings suggest that in-person surveys better represent the target population in rural communities than phone-based surveys.

There are several advantages and limitations to this analysis. Our study within a trial setting provided the opportunity to compare both survey modes with exhaustive and up-to-date census information, allowing us to robustly assess the representativeness of both survey strategies. This analysis also offered an advantage over others based in low-and-middle-income countries since it was conducted in a rural setting with regular community engagement and used the same questionnaire in both surveys within a 6-month time frame, thus allowing us to compare participation confidently [36–38]. The phone-based survey was conducted in a state with high teledensity and fair network coverage, however, even in rural areas. As a result, these findings may not be generalisable to all rural settings. In addition, this survey was conducted during the COVID-19 pandemic and during a time of lockdowns in the study region, which may have negatively impacted responsiveness to the phone survey. Our exploration of representativeness and characteristics associated with non-response included a range of measures collected during the trial census, but there may be other unmeasured factors that we have not accounted for in our analyses.

CONCLUSIONS

For peer review only - http://bmjopen.bmj.com/site/about/guidelines.xhtml

We found that phone-based surveys underrepresent groups at a higher risk of acquiring STH and other NTDs. As a result, phone surveys may bias coverage estimates and could potentially negatively impact the groups that most need treatment. Phone-based surveys are a convenient alternative, especially when circumstances preclude household visits and resources are limited, but in the absence of accurate, up-to-date data on population characteristics, in-person surveys appear more representative.

For peer review only

Patient consent for publication: Not required.

Ethics approval: The DeWorm3 study was approved by the Institutional Review Board of Christian Medical College, Vellore, India (10392, dated 30.11.2016), Human Subjects Division at the University of Washington (STUDY00000180) and The London School of Hygiene & Tropical Medicine (12013) and conformed to the principles embodied in the Declaration of Helsinki. For the in-person survey, written informed consent was sought from the head of the household or any adult member (≥18 years). For the phone-based surveys, the standardised information sheet was read out to the household head or the next eligible adult household member at the start of the call, and verbal consent over the phone was obtained. The change in protocol and current analysis was submitted to the Institutional Review Board of Christian Medical College, Vellore, as an amendment (IRB-A9-24.11.2021, dated 02.12.2022) and approved by The London School of Hygiene & Tropical Medicine (26333). The trial was registered at ClinicalTrials.gov (NCT03014167).

Acknowledgements: We thank field managers Rajeshkumar Rajendiran (Timiri) and Chinnaduraipandi (Jawadhu hills), Jasmine Farzana, Gokila Palaniswamy, Janarthanan Maniyarasu, Naveen Kumar Sekar and Dhanalakshmi Manoharan (at CMC, Vellore) and David S Kennedy (at LSHTM), for their meticulous work in developing and implementing the coverage data collection forms. We are most grateful to our field supervisors and field workers in Timiri and Jawadhu for exemplary work in completing the surveys despite the pandemic limitations. We acknowledge the contributions and efforts of the entire DeWorm3 Trials Team, particularly Dr Arianna Means, Dr Kristjana Ásbjörnsdóttir, and Emily Pearman at the University of Washington and Dr Tim Littlewood and Leanne Doran at the Natural History Museum.

For peer review only

We are thankful for support extended to this study from the Ministry of Health and Family Welfare, Delhi and the Directorate of Public Health, Chennai, as well as help in implementing study activities from officials at the district and block levels, village leaders and members of our community advisory boards in Timiri and Jawadhu hills. Most importantly, we thank all the participants for their time.

Contributors: RMR and WEO conceptualised the study, conducted the data analysis, and wrote the first draft with support from SSRA and KEH. JLW, the Deworm3 study PI, obtained funding for the study and helped write the paper. KA, SPK and GJI conceptualised the study, oversaw the analysis, and provided feedback on the draft. SG and HL provided access to the data and supported critical interpretation of the findings. All authors (RMR, WEO, GJI, KA, SG, HL, SPK, JLW, KEH and SSRA) provided feedback on the manuscript and approved the final version.

Funding: The DeWorm3 study is funded through a grant to the University of Washington, Seattle, Washington, United States of America, from the Bill and Melinda Gates Foundation (OPP1129535, PI JLW). Under the grant conditions of the Foundation, a Creative Commons Attribution 4.0 Generic License has already been assigned to the Author Accepted Manuscript version that might arise from this submission. The funders were not involved in the decision to publish the manuscript and had no role in data collection, analysis or publication of study results.

Competing interests: None declared

Provenance and peer review: Not commissioned; externally peer-reviewed.

For peer review only

Supplemental material: This content has been supplied by the author(s). It has not been vetted by BMJ Publishing Group Limited (BMJ) and may not have been peer-reviewed. Any opinions or recommendations discussed are solely those of the author(s) and are not endorsed by BMJ. BMJ disclaims all liability and responsibility arising from any reliance on the content. Where the content includes any translated material, BMJ does not warrant the accuracy and reliability of the translations (including but not limited to local regulations, clinical guidelines, terminology, drug names and drug dosages) and is not responsible for any error or omissions arising from translation and adaptation or otherwise.

For peer review only

Twitter:

Rohan Michael Ramesh: @rohan_mic

William Edward Oswald: @williameoswald

Sitara S.R. Ajjampur: @ajjampur_s

ORCID iDs:

Rohan Michael Ramesh: 0000-0002-1089-6037

William Edward Oswald: 0000-0002-5287-2834

Sitara S.R. Ajjampur: 0000-0003-3419-6577

Data availability statement

Data cannot be shared publicly because the study remains blinded to outcome data. Following unblinding of the study, data will be available upon reasonable request.

References

1 Kempf AM, Remington PL. New Challenges for Telephone Survey Research in the Twenty-First Century. *Annu Rev Public Health* 2007;**28**:113–26. doi:10.1146/annurev.publhealth.28.021406.144059

2 Centre for Disease Control. Statistical methodology of the National Immunization Survey,1994-2002. CDC, 2005. Available:https://stacks.cdc.gov/view/cdc/6573 (accessed 1 Oct 2021).

3 Remington PL, Smith MY, Williamson DF, *et al.* Design, characteristics, and usefulness of state-based behavioral risk factor surveillance: 1981-87. *Public Health Rep* 1988;**103**:366–75.

4 Coffey D, Hathi P, Khalid N, *et al.* Using phone surveys to measure mental health. Ideas For India, 2020. Available: http://www.ideasforindia.in/topics/human-development/using-phone-surveys-to-measure-mental-health.html (accessed 1 Oct 2021).

5 Link MW, Mokdad AH. Effects of survey mode on self-reports of adult alcohol consumption: a comparison of mail, web and telephone approaches. *J Stud Alcohol* 2005;**66**:239–45.doi:10.15288/jsa.2005.66.239

6 The World Bank. Mobile cellular subscriptions (per 100 people). TWB, 2020. Available: https://data.worldbank.org/indicator/IT.CEL.SETS.P2?view=map (accessed 6 Nov 2021).

7 Gibson DG, Pereira A, Farrenkopf BA, *et al.* Mobile Phone Surveys for Collecting Population-Level Estimates in Low- and Middle-Income Countries: A Literature Review. *JMIR* 2017;**19**:e7428.doi:10.2196/jmir.7428

8 Boland M, Sweeney M, Scallan E, *et al.* Emerging advantages and drawbacks of telephone surveying in public health research in Ireland and the U.K. *BMC Public Health* 2006;**6**:208.doi:10.1186/1471-2458-6-208

9 Block ES, Erskine L. Interviewing by Telephone: Specific Considerations, Opportunities, and Challenges. *Int J Qual Methods* 2012;**11**(4):428–45.doi:10.1177/160940691201100409

10 Szolnoki G, Hoffmann D. Online, face-to-face and telephone surveys—Comparing different sampling methods in wine consumer research. *Wine Economics and Policy* 2013;**2**:57–66.doi:10.1016/j.wep.2013.10.001

11 Ellis CH, Krosnick JA. Comparing Telephone and Face-to-Face Surveys in Terms of Sample Representativeness: A Meta-Analysis of Demographic Characteristics. American Nation election studies, 1999. Available: http://users.clas.ufl.edu/kenwald/pos6757/spring02/tch59.pdf (accessed 25 Aug 2020).

12 National Center for Health Statistics, Blumberg SJ, Lake JV. Wireless substitution : early release of estimates From the National Health Interview Survey, July-December 2020. National Center for Health Statistics, 2021. doi:10.15620/cdc:108678

For peer review only

13 Moura EC, Claro RM, Bernal R, *et al.* A feasibility study of cell phone and landline phone interviews for monitoring of risk and protection factors for chronic diseases in Brazil. *Cad Saúde Pública* 2011;**27**:277–86.doi:10.1590/S0102-311X2011000200009

14 Telecom Regulatory Authority of India. TRAI releases Telecom Subscription Data as on 31st July, 2021, TRAI 2021. Available: https://www.trai.gov.in/notifications/press-release/trai-releases-telecom-subscription-data-31st-july-2021 (accessed 1 Oct 2021).

15 International Institute for Population Sciences. National Family Health Survey (NFHS-4),India, 2015-16,IIPS 2017.Available: http://rchiips.org/nfhs/nfhs-4Reports/India.pdf (accessed 30 Apr 2020).

16 Crompton DWT. Preventive chemotherapy in human helminthiasis : coordinated use of anthelminthic drugs in control interventions : a manual for health professionals and programme managers. World Health Organization, 2006. Available:https://apps.who.int/iris/handle/10665/43545 (accessed 8 Nov 2021).

17 World Health Organisation. Preventive chemotherapy: Tools for improving the quality of reported data and information, 2019. Available:https://apps.who.int/iris/bitstream/handle/10665/329376/9789241516464-eng.pdf (accessed 8 Nov 2021).

18 USAID. Post MDA Survey Design: Monitoring treatment coverage of neglected disease control programs, in A toolkit developed for USAID Neglected Tropical Disease Control Project, 2009. Available:https://pdf.usaid.gov/pdf_docs/Pdacq848.pdf

19 World Health Organization. Monitoring and epidemiological assessment of mass drug administration in the global programme to eliminate lymphatic filariasis : a manual for national elimination programmes. *Suivi et évaluation épidémiologique du traitement médicamenteux de masse dans le cadre du Programme mondial pour l'élimination de la filariose lymphatique : manuel à l'intention des programmes nationauxd'élimination* Published Online First: 2011. Available: https://apps.who.int/iris/handle/10665/44580 (accessed 15 Nov 2021).

20 Ajjampur SSR, Kaliappan SP, Halliday KE, *et al.* Epidemiology of soil transmitted helminths and risk analysis of hookworm infections in the community: Results from the DeWorm3 Trial in southern India. *PLoS Negl Trop Dis* 2021;**15**:e0009338.doi:10.1371/journal.pntd.0009338

21 Ásbjörnsdóttir KH, Ajjampur SSR, Anderson RM, *et al.* Assessing the feasibility of interrupting the transmission of soil-transmitted helminths through mass drug administration: The DeWorm3 cluster randomized trial protocol. *PLoS Negl Trop Dis* 2018;**12**:e0006166.doi:10.1371/journal.pntd.0006166

22 DeWorm3 Trials Team. Baseline patterns of infection in regions of Benin, Malawi and India seeking to interrupt transmission of soil transmitted helminths

For peer review only

(STH) in the DeWorm3 trial. *PLoS Negl Trop Dis* 2020;**14**:e0008771.doi:10.1371/journal.pntd.0008771

23 Evidence Action. World's Largest Deworming Program in India To Start With Support from Evidence Action, 2015. Available:https://www.evidenceaction.org/largest-deworming-program-in-india-to-start-with-support-from-evidence-action/ (accessed 31 Jan 2022).

24 National Health Portal Of India. National Deworming Day, 2021. Available: https://www.nhp.gov.in/national-deworming-day-2021_pg (accessed 6 Apr 2022).

25. Zou G. A modified poisson regression approach to prospective studies with binary data. *Am J Epidemiol*. 2004; **159**:702-6. doi: 10.1093/aje/kwh090.

26. Heinze G, Wallisch C, Dunkler D. Variable selection - A review and recommendations for the practicing statistician. *Biom J.* 2018;**60**:431-449. doi: 10.1002/bimj.201700067

27 Tran MC, Labrique AB, Mehra S, *et al.* Analyzing the Mobile "Digital Divide": Changing Determinants of Household Phone Ownership Over Time in Rural Bangladesh. *JMIR mHealth and uHealth* 2015;**3**:e3663.doi:10.2196/mhealth.3663

28 Labrique A, Blynn E, Ahmed S, *et al.* Health Surveys Using Mobile Phones in Developing Countries: Automated Active Strata Monitoring and Other Statistical Considerations for Improving Precision and Reducing Biases. *JMIR* 2017;**19**:e7329.doi:10.2196/jmir.7329

29 Kumar C. TRAI: India lost 1.7 crore mobile subscribers in lockdown months. The Times of India, 2020. Available: https://timesofindia.indiatimes.com/india/trai-india-lost-1-7-crore-mobile-subscribers-in-lockdown-months/articleshow/79273419.cms (accessed 8 Nov 2021).

30 Centre for Sustainable Employment. Covid-19 Employment and Income Tracker,2020. Available:https://cse.azimpremjiuniversity.edu.in/covid19-data-tracker/ (accessed 1 Dec 2021).

31 Giorgia Barboni, Erica Field, Rohini Pande, *et al.* A Tough Call: Understanding barriers to and impacts of women's mobile phone adoption in India. Harvard Kennedy School, 2018. Available: https://research.hks.harvard.edu/publications/getFile.aspx?Id=2765

32 Greenleaf AR, Ahmed S, Moreau C, *et al.* Cell phone ownership and modern contraceptive use in Burkina Faso: implications for research and interventions using mobile technology. *Contraception* 2019;**99**:170. doi:10.1016/j.contraception.2018.11.006

33. Quota Sampling. In: *The SAGE Encyclopedia of Educational Research, Measurement, and Evaluation*. 2455 Teller Road, Thousand

For peer review only

Oaks, California 91320: SAGE Publications, Inc. 2018.doi:10.4135/9781506326139.n565

34 Dal Grande E, Chittleborough CR, Campostrini S, *et al.* Bias of health estimates obtained from chronic disease and risk factor surveillance systems using telephone population surveys in Australia: results from a representative face-to-face survey in Australia from 2010 to 2013. *BMC Med Res Methodol* 2016;**16**:44.doi:10.1186/s12874-016-0145-z

35 Aagaard-Hansen J, Chaignat CL. Neglected tropical diseases: equity and social determinants. World Health Organization 2010;**8**:135–157. Available:http://citeseerx.ist.psu.edu/viewdoc/download?doi=10.1.1.394.6922&rep=rep1&type=pdf (accessed 1 Dec 2021).

36 Nagpal K, Mathur MR, Biswas A, *et al*. Who do phone surveys miss, and how to reduce exclusion: recommendations from phone surveys in nine Indian states. *BMJ Glob Health* 2021;**6**:e005610.

37 Nambiar D, Benny G Usaidali. Telephony and trade-offs in fieldwork with the 'unreached': on the conduct of telephonic interviews with indigenous study participants in southern India. *BMJ Glob Health* 2021;**6:**e006261

38 Mohan D, Scott K, Shah N*, et al.* Can health information through mobile phones close the divide in health behaviours among the marginalised? An equity analysis of Kilkari in Madhya Pradesh, India. *BMJ Glob Health* 2021;**6:**e005512.

Figure titles

Figure 1. Timeline of phone-based coverage evaluation survey in relation to other key activities

Figure 2. Household participation details of in-person and phone-based coverage surveys

For peer review only

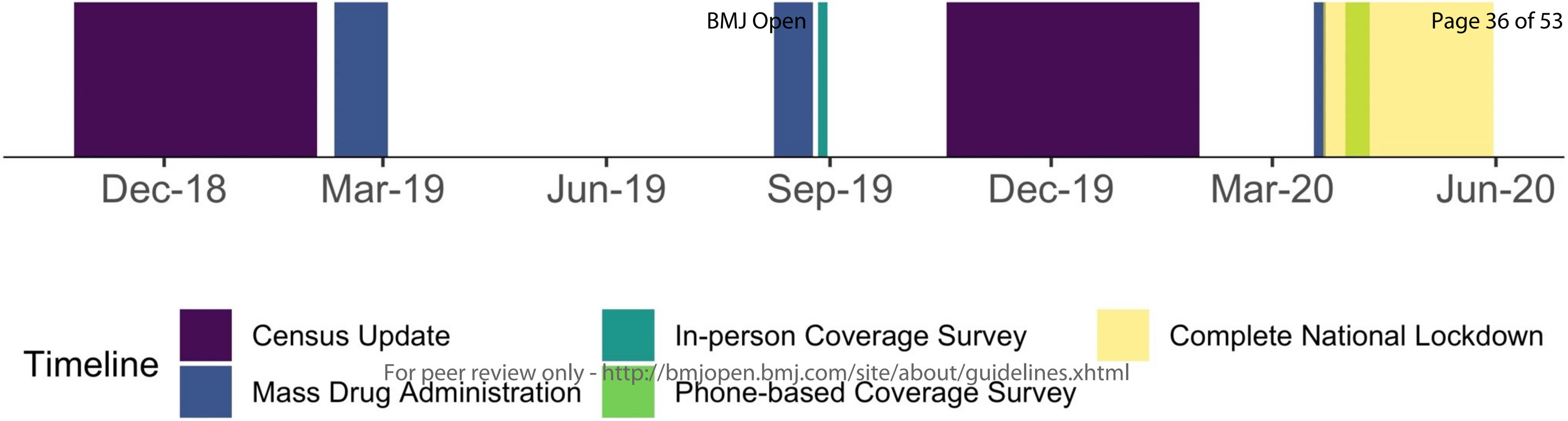
Dec-18
Mar-19
Jun-19
Sep-19
Dec-19
Mar-20
Jun-20
Timeline
Census Update
Mass Drug Administration
In-person Coverage Survey
Phone-based Coverage Survey
Complete National Lockdown

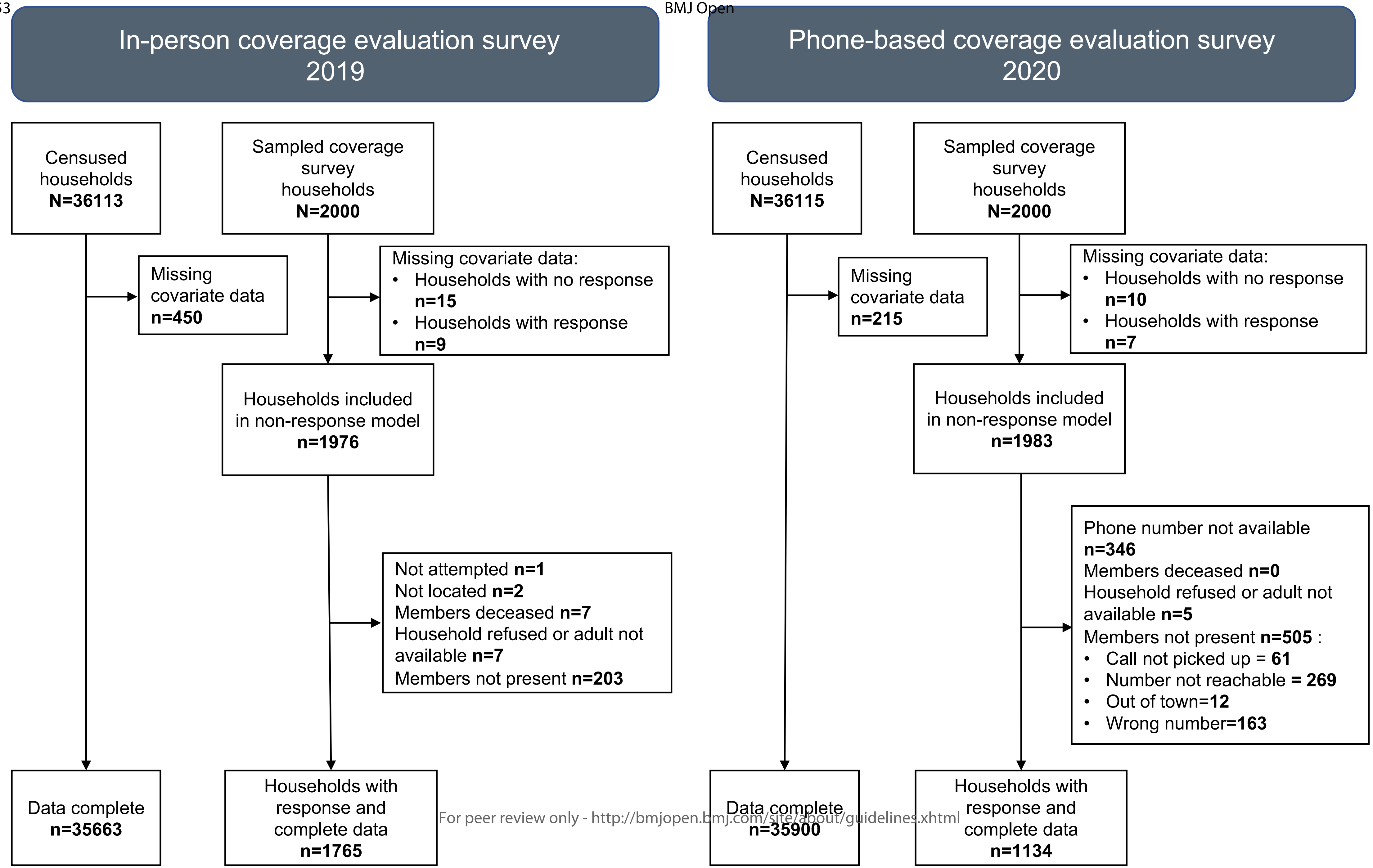

For peer review only - http://bmjopen.bmj.com/site/about/guidelines.xhtml

Supplementary Table 1 - Factors associated with non-response in a phone-based coverage evaluation survey, including mobile phone ownership

	Phone-based coverage survey					
	Sampled households† N=1983 (100) n (%)	Non response‡ n=849 (42.8) n (%)	Univariate PR (95% CI)	P value	Multivariable PR (95% CI)	P value
Site details						
**Proportion of households in each arm**						
Control	993 (50.1)	429 (43.2)	REF	0.78		
Intervention	990 (49.9)	420 (42.4)	1.0 (0.9, 1.1)			
**Proportion of households per site**						
Timiri	1586 (80.0)	694 (43.8)	REF	0.45	REF	**0.04**
Jawadhu Hills	397 (20.0)	155 (39.0)	0.9 (0.7, 1.2)		0.7 (0.6, 1.0)	
Household characteristics						
**Religion**						
Other	68 (3.4)	27 (39.7)	REF	0.67		
Hindu	1915 (96.6)	822 (42.9)	1.1 (0.8, 1.5)			
**Caste**						
Higher caste	20 (1.0)	6 (30.0)	REF	0.50		
Backward caste	635 (32.0)	271 (42.7)	1.4 (0.8, 2.6)			
Most backward caste	487 (24.6)	213 (43.7)	1.5 (0.8, 2.7)			
Scheduled caste	439 (22.1)	198 (45.1)	1.5 (0.9, 2.6)			
Scheduled tribes	402 (20.3)	161 (40.0)	1.3 (0.7, 2.5)			
**House type§**						
Concrete	1054 (53.2)	417 (39.6)	REF	**0.01**		
Mixed	233 (11.7)	113 (48.5)	1.2 (1.1, 1.4)			
Government funded/ donated house	200 (10.1)	95 (47.5)	1.2 (1.0, 1.5)			
Thatched	496 (25.0)	224 (45.2)	1.1 (1.0, 1.3)			
**Socio-economic quintiles**						
Least poorest quintile	422 (21.3)	110 (26.1)	REF	**<0.01**	REF	**<0.01**
4th quintile	390 (19.7)	161 (41.3)	1.6 (1.3, 1.9)		1.5 (1.2, 1.8)	
3rd quintile	406 (20.5)	185 (45.6)	1.7 (1.4, 2.1)		1.5 (1.3, 1.9)	
2th quintile	386 (19.5)	191 (49.5)	1.9 (1.5, 2.3)		1.6 (1.4, 1.9)	
Poorest quintile	379 (19.1)	202 (53.3)	2.0 (1.7, 2.5)		1.7 (1.4, 2.1)	
**Large household (family size)**						
No (<= 4 members)	1286 (64.9)	603 (46.9)	REF	**<0.01**		

Yes (>= 5 members)	697 (35.1)	246 (35.3)	0.8 (0.7, 0.8)			
**Owns mobile phone**						
No	298 (15.0)	255 (85.6)	REF	**<0.01**	REF	**<0.01**
Yes	1685 (85.0)	594 (35.3)	0.4 (0.4, 0.5)		0.5 (0.4, 0.5)	
Head of the household characteristics						
**Age**						
18-30 years	84 (4.2)	31 (36.9)	REF	**<0.01**		
31-40 years	388 (19.6)	142 (36.6)	1.0 (0.8, 1.3)			
41-50 years	516 (26.0)	185 (35.9)	1.0 (0.8, 1.2)			
51-60 years	431 (21.7)	184 (42.7)	1.2 (0.9, 1.5)			
>61 years	564 (28.4)	307 (54.4)	1.5 (1.1, 2.0)			
**Sex**						
Male	1602 (80.8)	638 (39.8)	REF	<0.01		
Female	381 (19.2)	211 (55.4)	1.4 (1.2, 1.6)			
**Education level**						
No education	587 (29.6)	317 (54.0)	REF	**<0.01**		
Any primary	452 (22.8)	196 (43.4)	0.8 (0.7, 0.9)			
Any middle	343 (17.3)	141 (41.1)	0.8 (0.6, 0.9)			
Any secondary or higher	601 (30.3)	195 (32.4)	0.6 (0.5, 0.7)			
**Marriage status**						
Never married	397 (20.0)	229 (57.7)	REF	**<0.01**		
Ever married	1586 (80.0)	620 (39.1)	0.7 (0.6, 0.8)			

Acronyms: PR - Prevalence Ratio; CI - Confidence Interval

†Number (N) of sampled households with complete covariate data used to estimate prevalence of non-response in each category.
‡Non-response - no household members available for interview, household refused to participate or unable to consent, or the household was not attempted.
§Concrete - concrete walls and roof; Mixed - concrete walls and tiled roof; Government donated/funded house - prebuilt government houses or houses funded through schemes for economically and socially marginalized groups; Thatched - thatched walls and roof.

Supplementary Table 2 - Factors associated with non-response in an in-person coverage evaluation survey, including mobile phone ownership

	In-person coverage survey					
	**Sampled households†**	**Non response‡**	**Univariate**		**Multivariable**	
	**N=1976 (100) n (%)**	**n=211 (10.7) n (%)**	**PR (95% CI)**	**P value**	**PR (95% CI)**	**P value**
Site details						
**Proportion of households in each arm**						
Control	990 (50.1)	110 (11.1)	REF	0.65		
Intervention	986 (49.9)	101 (10.2)	0.9 (0.7, 1.3)			
**Proportion of households per site**						
Timiri	1581 (80.0)	187 (11.8)	REF	**<0.01**	REF	**<0.01**
Jawadhu Hills	395 (20.0)	24 (6.1)	0.5 (0.3, 0.8)		0.5 (0.3, 0.8)	
Household characteristics						
**Religion**						
Other	58 (2.9)	13 (22.4)	REF	**<0.01**		
Hindu	1918 (97.1)	198 (10.3)	0.5 (0.3, 0.7)			
**Caste**						
Higher caste	18 (0.9)	1 (5.6)	REF	0.18		
Backward caste	609 (30.8)	73 (12.0)	2.2 (0.3, 16.0)			
Most backward caste	502 (25.4)	62 (12.4)	2.2 (0.3, 16.2)			
Scheduled caste	439 (22.2)	46 (10.5)	1.9 (0.2, 14.4)			
Scheduled tribes	408 (20.6)	29 (7.1)	1.3 (0.2, 9.8)			
**House type§**						
Concrete	1019 (51.6)	123 (12.1)	REF	0.02		
Mixed	229 (11.6)	26 (11.4)	0.9 (0.6, 1.4)			
Government funded/ donated house	191 (9.7)	24 (12.6)	1.0 (0.7, 1.5)			
Thatched	537 (27.2)	38 (7.1)	0.6 (0.4, 0.9)			
**Socio-economic quintiles**						
Least poorest quintile	391 (19.8)	48 (12.3)	REF	**0.02**		
4th quintile	360 (18.2)	39 (10.8)	0.9 (0.6, 1.3)			
3th quintile	429 (21.7)	54 (12.6)	1.0 (0.7, 1.6)			
2th quintile	410 (20.7)	47 (11.5)	0.9 (0.6, 1.5)			
Poorest quintile	386 (19.5)	23 (6.0)	0.5 (0.3, 0.8)			
**Large household (family size)**						

No (<= 4 members)	1339 (67.8)	159 (11.9)	REF	**0.02**		
Yes (>= 5 members)	637 (32.2)	52 (8.2)	0.7 (0.5, 0.9)			
**Owns mobile phone**						
No	293 (14.8)	25 (8.5)	REF	0.16		
Yes	167 (10.8)	1.3 (0.9, 1.8)	1.3 (0.9, 1.8)			
Data not available	134 (6.8)	19 (14.2)	1.7 (1.0, 2.8)			
Head of the household characteristics						
**Age**						
18-30 years	108 (5.5)	16 (14.8)	REF	**0.03**	REF	**<0.01**
31-40 years	403 (20.4)	55 (13.6)	0.9 (0.6, 1.5)		0.8 (0.5, 1.3)	
41-50 years	489 (24.7)	38 (7.8)	0.5 (0.3, 0.9)		0.4 (0.3, 0.7)	
51-60 years	455 (23.0)	53 (11.6)	0.8 (0.5, 1.3)		0.6 (0.4, 1.0)	
>61 years	521 (26.4)	49 (9.4)	0.6 (0.3, 1.2)		0.5 (0.3, 0.9)	
**Sex**						
Male	1567 (79.3)	155 (9.9)	REF	**0.02**		
Female	409 (20.7)	56 (13.7)	1.4 (1.1, 1.8)			
**Education level**						
No education	655 (33.1)	67 (10.2)	REF	0.36		
Any primary	427 (21.6)	48 (11.2)	1.1 (0.8, 1.5)			
Any middle	360 (18.2)	31 (8.6)	0.8 (0.5, 1.4)			
Any secondary or higher	534 (27.0)	65 (12.2)	1.2 (0.9, 1.6)			
**Marriage status**						
Never married	377 (19.1)	53 (14.1)	REF	**0.01**		
Ever married	1599 (80.9)	158 (9.9)	0.7 (0.5, 0.9)			

Acronyms: PR - Prevalence Ratio; CI - Confidence Interval

†Number (N) of sampled households with complete covariate data used to estimate prevalence of non-response in each category.

‡Non-response - no household members available for interview, household refused to participate or unable to consent, or the household was not attempted.

§Concrete - concrete walls and roof; Mixed - concrete walls and tiled roof; Government donated/funded house - prebuilt government houses or houses funded through schemes for economically and socially marginalized groups; Thatched - thatched walls and roof.

Supplementary Table 3 - Factors associated with non-response in a phone-based coverage survey, including phone number availability

	Phone-based coverage survey					
	**Sampled households†**	**Non response‡**	**Univariate**		**Multivariable**	
	**N=1983 (100) n (%)**	**n=849 (42.8) n (%)**	**PR (95% CI)**	**P value**	**PR (95% CI)**	**P value**
Site details						
**Proportion of households in each arm**						
Control	993 (50.1)	429 (43.2)	REF	0.78		
Intervention	990 (49.9)	420 (42.4)	1.0 (0.9, 1.1)			
**Proportion of households per site**						
Timiri	1586 (80.0)	694 (43.8)	REF	0.45	REF	**0.08**
Jawadhu Hills	397 (20.0)	155 (39.0)	0.9 (0.7, 1.2)		0.8 (0.6, 1.0)	
Household characteristics						
**Religion**						
Other	68 (3.4)	27 (39.7)	REF	0.67		
Hindu	1915 (96.6)	822 (42.9)	1.1 (0.8, 1.5)			
**Caste**						
Higher caste	20 (1.0)	6 (30.0)	REF	0.50		
Backward caste	635 (32.0)	271 (42.7)	1.4 (0.8, 2.6)			
Most backward caste	487 (24.6)	213 (43.7)	1.5 (0.8, 2.7)			
Scheduled caste	439 (22.1)	198 (45.1)	1.5 (0.9, 2.6)			
Scheduled tribes	402 (20.3)	161 (40.0)	1.3 (0.7, 2.5)			
**House type§**						
Concrete	1054 (53.2)	417 (39.6)	REF	**0.01**		
Mixed	233 (11.7)	113 (48.5)	1.2 (1.1, 1.4)			
Government funded/ donated house	200 (10.1)	95 (47.5)	1.2 (1.0, 1.5)			
Thatched	496 (25.0)	224 (45.2)	1.1 (1.0, 1.3)			
**Socio-economic quintiles**						
Least poorest quintile	422 (21.3)	110 (26.1)	REF	**<0.01**	REF	**<0.01**
4th quintile	390 (19.7)	161 (41.3)	1.6 (1.3, 1.9)		1.5 (1.3, 1.9)	
3th quintile	406 (20.5)	185 (45.6)	1.7 (1.4, 2.1)		1.5 (1.2, 1.7)	
2th quintile	386 (19.5)	191 (49.5)	1.9 (1.5, 2.3)		1.6 (1.4, 1.9)	
Poorest quintile	379 (19.1)	202 (53.3)	2.0 (1.7, 2.5)		1.7 (1.4, 2.0)	
**Large household (family size)**						
No (<= 4 members)	1286 (64.9)	603 (46.9)	REF	**<0.01**		

Yes (>= 5 members)	697 (35.1)	246 (35.3)	0.8 (0.7, 0.8)			
**Phone number available**						
No	405 (20.4)	382 (94.3)	REF	**<0.01**	REF	**<0.01**
Yes	1578 (79.6)	467 (29.6)	0.3 (0.3, 0.4)		0.3 (0.3, 0.4)	
Head of the household characteristics						
**Age**						
18-30 years	84 (4.2)	31 (36.9)	REF	**<0.01**		
31-40 years	388 (19.6)	142 (36.6)	1.0 (0.8, 1.3)			
41-50 years	516 (26.0)	185 (35.9)	1.0 (0.8, 1.2)			
51-60 years	431 (21.7)	184 (42.7)	1.2 (0.9, 1.5)			
>61 years	564 (28.4)	307 (54.4)	1.5 (1.1, 2.0)			
**Sex**						
Male	1602 (80.8)	638 (39.8)	REF	**<0.01**		
Female	381 (19.2)	211 (55.4)	1.4 (1.2, 1.6)			
**Education level**						
No education	587 (29.6)	317 (54.0)	REF	**<0.01**		
Any primary	452 (22.8)	196 (43.4)	0.8 (0.7, 0.9)			
Any middle	343 (17.3)	141 (41.1)	0.8 (0.6, 0.9)			
Any secondary or higher	601 (30.3)	195 (32.4)	0.6 (0.5, 0.7)			
**Marriage status**						
Never married	397 (20.0)	229 (57.7)	REF	**<0.01**		
Ever married	1586 (80.0)	620 (39.1)	0.7 (0.6, 0.8)			

Acronyms: PR - Prevalence Ratio; CI - Confidence Interval

†Number (N) of sampled households with complete covariate data used to estimate prevalence of non-response in each category.
‡Non-response - no household members available for interview, household refused to participate or unable to consent, or the household was not attempted.
§Concrete - concrete walls and roof; Mixed - concrete walls and tiled roof; Government donated/funded house - prebuilt government houses or houses funded through schemes for economically and socially marginalized groups; Thatched - thatched walls and roof.

For peer review only

Supplementary Table 4 - Factors associated with non-response in an in-person coverage survey, including phone number availability

	In-person coverage survey					
	**Sampled households†**	**Non response‡**	**Univariate**		**Multivariable**	
	**N=1976 (100) n (%)**	**n=211 (10.7) n (%)**	**PR (95% CI)**	**P value**	**aPR (95% CI)**	**P value**
Site details						
**Proportion of households in each arm**						
Control	990 (50.1)	110 (11.1)	REF	0.65		
Intervention	986 (49.9)	101 (10.2)	0.9 (0.7, 1.3)			
**Proportion of households per site**						
Timiri	1581 (80.0)	187 (11.8)	REF	**<0.01**	REF	**<0.01**
Jawadhu Hills	395 (20.0)	24 (6.1)	0.5 (0.3, 0.8)		0.5 (0.3, 0.8)	
Household characteristics						
**Religion**						
Other	58 (2.9)	13 (22.4)	REF	**<0.01**		
Hindu	1918 (97.1)	198 (10.3)	0.5 (0.3, 0.7)			
**Caste**						
Higher caste	18 (0.9)	1 (5.6)	REF	0.18		
Backward caste	609 (30.8)	73 (12.0)	2.2 (0.3, 16.0)			
Most backward caste	502 (25.4)	62 (12.4)	2.2 (0.3, 16.2)			
Scheduled caste	439 (22.2)	46 (10.5)	1.9 (0.2, 14.4)			
Scheduled tribes	408 (20.6)	29 (7.1)	1.3 (0.2, 9.8)			
**House type§**						
Concrete	1019 (51.6)	123 (12.1)	REF	0.02		
Mixed	229 (11.6)	26 (11.4)	0.9 (0.6, 1.4)			
Government funded/ donated house	191 (9.7)	24 (12.6)	1.0 (0.7, 1.5)			
Thatched	537 (27.2)	38 (7.1)	0.6 (0.4, 0.9)			
**Socio-economic quintiles**						
Least poor quintile	391 (19.8)	48 (12.3)	REF	**0.02**		
4th quintile	360 (18.2)	39 (10.8)	0.9 (0.6, 1.3)			
3th quintile	429 (21.7)	54 (12.6)	1.0 (0.7, 1.6)			
2th quintile	410 (20.7)	47 (11.5)	0.9 (0.6, 1.5)			
Poorest quintile	386 (19.5)	23 (6.0)	0.5 (0.3, 0.8)			
**Large household (family size)**						
No (<= 4 members)	1339 (67.8)	159 (11.9)	REF	**0.02**		

For peer review only

Yes (>= 5 members)	637 (32.2)	52 (8.2)	0.7 (0.5, 0.9)			
**Phone number available**						
No	273 (13.8)	26 (9.5)	REF	**<0.01**		
Yes	1703 (86.2)	185 (10.9)	1.1 (0.8, 1.6)			
Head of the household characteristics						
**Age**						
18-30 years	108 (5.5)	16 (14.8)	REF	**0.03**	REF	**<0.01**
31-40 years	403 (20.4)	55 (13.6)	0.9 (0.6, 1.5)		0.8 (0.5, 1.3)	
41-50 years	489 (24.7)	38 (7.8)	0.5 (0.3, 0.9)		0.4 (0.3, 0.7)	
51-60 years	455 (23.0)	53 (11.6)	0.8 (0.5, 1.3)		0.6 (0.4, 1.0)	
>61 years	521 (26.4)	49 (9.4)	0.6 (0.3, 1.2)		0.5 (0.3, 0.9)	
**Sex**						
Male	1567 (79.3)	155 (9.9)	REF	**0.02**		
Female	409 (20.7)	56 (13.7)	1.4 (1.1, 1.8)			
**Education level**						
No education	655 (33.1)	67 (10.2)	REF	0.36		
Any primary	427 (21.6)	48 (11.2)	1.1 (0.8, 1.5)			
Any middle	360 (18.2)	31 (8.6)	0.8 (0.5, 1.4)			
Any secondary or higher	534 (27.0)	65 (12.2)	1.2 (0.9, 1.6)			
**Marriage status**						
Never married	377 (19.1)	53 (14.1)	REF	**0.01**		
Ever married	1599 (80.9)	158 (9.9)	0.7 (0.5, 0.9)			

Acronyms: PR - Prevalence Ratio; CI - Confidence Interval

†Number (N) of sampled households with complete covariate data used to estimate prevalence of non-response in each category.

‡Non-response - no household members available for interview, household refused to participate or unable to consent, or the household was not attempted.

§Concrete - concrete walls and roof; Mixed - concrete walls and tiled roof; Government donated/funded house - prebuilt government houses or houses funded through schemes for economically and socially marginalized groups; Thatched - thatched walls and roof.

For peer review only

Supplementary Table 5 - Phone number availability among mobile phone ownership reported in the annual census

Owns mobile phone	In-person coverage survey households (n=1563)	Phone-based coverage survey households (n=1700)	P value
Household phone number available			
**No**	102 (6.5)	176 (10.4)	**<0.01**
**Yes**	1461 (93.5)	1524 (89.6)	

For peer review only

Supplementary Table 6 - Factors associated with reported non-ownership of a mobile phone in Tamil Nadu, 2019

	Censused households 2019-2020 N=37883 (100) n (%)	Non ownership N=5728 (15.1) n (%)	Univariate PR (95% CI)	P value	Multivariable PR (95% CI)	P value
Site details						
**Proportion of households in each arm**						
Control	18424 (48.6)	2800 (15.2)	REF	0.91		
Intervention	19459 (51.4)	2928 (15.0)	1.0 (0.8, 1.2)			
**Proportion of households per site**						
Timiri	29545 (78.0)	4159 (14.1)	REF	**<0.01**	REF	**<0.01**
Jawadhu Hills	8338 (22.0)	1569 (18.8)	1.3 (1.1, 1.7)		0.4 (0.3, 0.4)	
Household characteristics						
**Religion**						
Other	1133 (3.0)	93 (8.2)	REF	**<0.01**		
Hindu	36750 (97.0)	5635 (15.3)	1.9 (1.5, 2.4)			
**Caste**						
Higher	430 (1.1)	50 (11.6)	REF	**<0.01**		
Backward	11362 (30.0)	1406 (12.4)	1.1 (0.8, 1.5)			
Most backward	9207 (24.3)	1334 (14.5)	1.2 (0.9, 1.8)			
Scheduled	8457 (22.3)	1264 (14.9)	1.3 (0.9, 1.8)			
Scheduled tribes	8427 (22.2)	1674 (19.9)	1.7 (1.2, 2.4)			
**House type†**						
Concrete	20055 (52.9)	1861 (9.3)	REF	**<0.01**		
Mixed	4190 (11.1)	750 (17.9)	1.9 (1.8, 2.1)			
Government funded/ donated house	3518 (9.3)	626 (17.8)	1.9 (1.7, 2.1)			
Thatched	10120 (26.7)	2491 (24.6)	2.7 (2.4, 2.9)			
**Socio-economic quintiles**						
Least poor quintile	7573 (20.0)	132 (1.7)	REF	**<0.01**	REF	**<0.01**
4th quintile	7569 (20.0)	302 (4.0)	2.3 (1.8, 2.9)		2.0 (1.6, 2.5)	
3th quintile	7540 (19.9)	876 (11.6)	6.7 (5.3, 8.4)		5.2 (4.1, 6.5)	
2th quintile	7598 (20.1)	1750 (23.0)	13.2 (9.9, 17.7)		10.9 (8.5, 14.1)	
Poorest quintile	7603 (20.1)	2668 (35.1)	20.1 (15.4, 26.3)		19.3 (15.3, 24.3)	
**Large household (family size)**						

No (<= 4 members)	24965 (65.9)	4947 (19.8)	REF	**<0.01**		
Yes (>= 5 members)	12918 (34.1)	781 (6.0)	0.3 (0.3, 0.4)			
**Head of the household characteristics**						
**Age**						
18-30 years	1600 (4.2)	144 (9.0)	REF	**<0.01**		
31-40 years	7230 (19.1)	538 (7.4)	0.8 (0.7, 1.0)			
41-50 years	9708 (25.6)	783 (8.1)	0.9 (0.7, 1.1)			
51-60 years	8848 (23.4)	1160 (13.1)	1.5 (1.1, 1.9)			
>61 years	10497 (27.7)	3103 (29.6)	3.3 (2.5, 4.2)			
**Sex**						
Male	30800 (81.3)	3203 (10.4)	REF	**<0.01**		
Female	7083 (18.7)	2525 (35.6)	3.4 (3.1, 3.7)			
**Education level**						
No education	11581 (30.6)	3518 (30.4)	REF	**<0.01**	REF	**<0.01**
Any primary	8613 (22.7)	1240 (14.4)	0.5 (0.4, 0.5)		0.6 (0.6, 0.6)	
Any middle	6762 (17.8)	517 (7.6)	0.3 (0.2, 0.3)		0.4 (0.3, 0.4)	
Any secondary or higher	10927 (28.8)	453 (4.1)	0.1 (0.1, 0.2)		0.3 (0.3, 0.3)	
**Marriage status**						
Never married	7843 (20.7)	2750 (35.1)	REF	**<0.01**		
Ever married†	30040 (79.3)	2978 (9.9)	0.3 (0.3, 0.3)			

Acronyms: PR - Prevalence Ratio; CI - Confidence Interval

† Concrete - concrete walls and roof; Mixed - concrete walls and tiled roof; Government donated/funded house - prebuilt government houses or houses funded through schemes for economically and socially marginalized groups; Thatched - thatched walls and roof

For peer review only

Coverage Evaluation Survey Questionnaire

1	Name of the participant		
2	Sex if the participant		
3	Age of the participant in years		
4.1	Can the participant answer the questions now?	1<br>2<br>3<br>4<br>5	Yes, agrees to participate<br>Not present<br>No, refused<br>No, other<br>Proxy respondent
4.2	Why has household refused to participate?	1<br>2<br>3<br>4<br>5<br>6.	Refused to speak/Not interested<br>Prior negative experience<br>Unwilling to provide detailed household information<br>Unwilling because of concerns related to risks, confidentiality, etc.<br>Unwilling to sign/provide thumb impression<br>Other
4.3	If unavailable, where is the participant?	1<br>2<br>3<br>4<br>5<br>6<br>7<br>8.	At school<br>At college<br>At work<br>Not at home (e.g at shop)<br>Moved away permanently<br>Temporarily migrated<br>Died<br>Other
5	Name of the proxy respondent		
6	Sex of the proxy respondent		
7	Age of the proxy respondent		
8	Did someone give you a tablet in the past month to treat worms (at home, at school, or in the community)?	1<br>2<br>3.<br>4.	Yes<br>No<br>Don't know/Cannot recall<br>Refused
8.1	If 'No', Why didn't you receive the tablet?	1<br>2<br>3<br><br>4<br>5<br>6<br>7<br>8<br>9<br>10<br>11<br>12<br>13<br>14<br>15<br>16	Unaware of MDA<br>Distributor did not come to my community<br>Distributor came to my community but did not come to my house<br>Distributor did not come to my school<br>The distributor was out of tablets and did not return<br>Pregnant<br>Breastfeeding<br>Ill<br>Told I could not take a tablet that day for other reasons<br>Too old<br>Underage (less than 1yo)<br>I was busy<br>I was too far away<br>Other<br>Don't know/Cannot recall<br>Refused
8.2	If 'Yes', Did you swallow the tablet?	1<br>2<br>3<br>4	Yes<br>No<br>Don't know/Cannot recall<br>Refused
8.3	Why did you not swallow the tablet?	1<br>2<br>3<br>4<br>5<br>6<br>7<br>8<br>9<br>10<br>11<br>12	Pregnant<br>Breastfeeding<br>Ill<br>Too old<br>Underage (less than 1yo)<br>Afraid of side effects<br>I am not at risk for this disease<br>I am taking other medications<br>Medicine doesn't work<br>Other<br>Don't know/Cannot recall<br>Refused
8.4	Why did you swallow the tablet?	1<br>2<br>3<br>4<br>5	To be healthy<br>Afraid of getting the disease<br>Someone told me to<br>Everyone else is taking it<br>Don't know/Cannot recall

For peer review only

		6	Refused
		7	Other
9	Did you swallow the tablet in front of the person who gave it to you?	1	Yes
		2	No
		3	Don't know/Cannot recall
		4	Refused
10	Who gave you the tablet?	1	Family member
		2	Health staff
		3	Field worker
		4	Drug distributor
		5	Community leader
		6	Teacher /Anganwadi worker
		7	Other
		8	Don't know/Cannot recall
		9	Refused
11	Where did they give you the tablet?	1	At home
		2	At school
		3	At Anganwadi centre
		4	Elsewhere in community
		5	Other
		6	Don't know/Cannot recall
		7	Refused
12	Did you feel any side effects after swallowing the tablet?	1	Yes
		2	No
		3	Don't know/Cannot recall
		4	Refused
13.1	Which of the following statements are true about side effects you experienced after swallowing the tablet?	1	Mild (did not interfere with daily activities)
		2	Moderate (interfered with daily activities)
		3	Serious (required hospitalization)
		4	Don't know/Cannot recall
		5	Refused
14	How many of these tablets did you swallow?	1	1/2
		2	1
		3	2
		4	3
		5	4
		6	5+
		7	Don't know/Cannot recall
		8	Refused
15	Were you aware that tablets would be distributed?	1	Yes
		2	No
		3	Don't know/Cannot recall
		4	Refused
16	How did you know that the tablets would be distributed?	1	Family member/friend/neighbor
		2	Professional health staff
		3	Community drug distributor/community health worker/teacher
		4	Community or religious leader
		5	Brochures/flyers
		6	Posters
		7	Banners
		8	Radio
		9	TV
		10	Social media
		11	Community Theatre/Drama
		12	Gram sabha
		13	Don't know/Cannot recall
		14	Refused to answer
		15	Other
17	Did any of your neighbors take the distributed tablet?	1	Yes
		2	No
		3	Don't know/Cannot recall
		4	Refused
18	What did you like about the community treatment programme?	1	House to house treatment makes it easy
		2	Trusted distributors
		3	No long wait for drugs
		4	Free drugs
		5	No specific part I liked
		6	Don't know/Cannot recall
		7	Refused to answer

For peer review only

		8	Other
19	What did you not like about the community treatment programme?	1<br>2<br>3<br>4<br>5<br>6<br>7<br>8<br>9<br>10	Inconvenient time<br>Drugs ran out or were not available<br>Unfriendly distributor<br>Took too much time<br>Adverse reaction to drug<br>Did not also give treatment for other diseases<br>No specific part I disliked<br>Don't know/Cannot recall<br>Refused to answer<br>Other
20	Next time the tablets are given, how would you want them distributed?	1<br>2<br>3<br>4<br>5<br>6	The same door-to-door<br>All individuals go to school<br>Distributors in central places in the village<br>Don't know/Cannot recall<br>Refused to answer<br>Other
21	Did you have to change your daily routine to participate in the treatment day?	1<br>2<br>3<br>4	Yes<br>No<br>Don't know/Cannot recall<br>Refused
22	About how much of your time did it take to participate in the treatment day, including the time you spent waiting for the drug distributor to arrive?	1<br>2<br>3<br>4<br>5<br>6<br>7<br>8	Less than one hour<br>About one hour<br>Half of a day<br>A full day<br>More than one day, distributor did not come on day planned<br>Don’t know/Cannot recall<br>Refused to say<br>Other
23	About how many minutes did the drug distributor spend at your house on the treatment day?	1<br>2<br>3<br>4<br>5<br>6<br>7<br>8<br>9<br>10<br>11	Less than 5 minutes<br>About 10 minutes<br>About 20 minutes<br>About 30 minutes<br>About 45 minutes<br>About one hour<br>More than one hour<br>Drugs were left here when we were not home<br>Don't know/Cannot recall<br>Refused to answer<br>Other
24	If there are no adult members available, where are they?	1<br>2<br>3	Not at home (e.g., at shop)<br>Moved away permanently<br>Temporarily migrated

For peer review only

STROBE Statement—Checklist of items that should be included in reports of ***cross-sectional studies***

	Item No	Recommendation	Page No
**Title and abstract**	1	*(a)* Indicate the study's design with a commonly used term in the title or the abstract **DONE – in the Abstract**	3
		(b) Provide in the abstract an informative and balanced summary of what was done and what was found **DONE – in the Abstract**	3
**Introduction**			
Background/rationale	2	Explain the scientific background and rationale for the investigation being reported **DONE – See Introduction section**	5-6
Objectives	3	State specific objectives, including any prespecified hypotheses **DONE – See Introduction section**	6
**Methods**			
Study design	4	Present key elements of study design early in the paper **DONE – See Methods section**	7-9
Setting	5	Describe the setting, locations, and relevant dates, including periods of recruitment, exposure, follow-up, and data collection **DONE – See Methods section**	7-9
Participants	6	*(a)* Give the eligibility criteria, and the sources and methods of selection of participants **DONE – See Methods section**	7-9
Variables	7	Clearly define all outcomes, exposures, predictors, potential confounders, and effect modifiers. Give diagnostic criteria, if applicable **DONE – See Methods section**	8-11
Data sources/ measurement	8*	For each variable of interest, give sources of data and details of methods of assessment (measurement). Describe comparability of assessment methods if there is more than one group **DONE – See Methods section**	*10-11*
Bias	9	Describe any efforts to address potential sources of bias **N/A**	
Study size	10	Explain how the study size was arrived at **DONE – See Methods section**	8-11
Quantitative variables	11	Explain how quantitative variables were handled in the analyses. If applicable, describe which groupings were chosen and why **DONE – See Methods section**	10-11
Statistical methods	12	*(a)* Describe all statistical methods, including those used to control for confounding **DONE – See Methods section**	10-11
		(b) Describe any methods used to examine subgroups and interactions **DONE – See Methods section**	10-11
		(c) Explain how missing data were addressed **N/A**	-

For peer review only - http://bmjopen.bmj.com/site/about/guidelines.xhtml

		(d) If applicable, describe analytical methods taking account of sampling strategy<br>**N/A**	-
		(e) Describe any sensitivity analyses<br>**DONE – See Methods and Analysis section**	11
**Results**			
Participants	13*	(a) Report numbers of individuals at each stage of study—eg numbers potentially eligible, examined for eligibility, confirmed eligible, included in the study, completing follow-up, and analysed<br>**DONE – See Results section/Figure 2**	12-13
		(b) Give reasons for non-participation at each stage<br>**DONE – See Results section**	12-13
		(c) Consider use of a flow diagram<br>**DONE – See Results section/ Figure 2**	12-13
Descriptive data	14*	(a) Give characteristics of study participants (eg demographic, clinical, social) and information on exposures and potential confounders<br>**DONE – See Results section**	13-15
		(b) Indicate number of participants with missing data for each variable of interest<br>**DONE – See Results section/Figure 2**	12-13
Outcome data	15*	Report numbers of outcome events or summary measures<br>**DONE – See Results section**	13-23
Main results	16	*(a)* Give unadjusted estimates and, if applicable, confounder-adjusted estimates and their precision (eg, 95% confidence interval). Make clear which confounders were adjusted for and why they were included<br>**DONE – See Results section**	13-23
		(b) Report category boundaries when continuous variables were categorized<br>**DONE – See Results section**	13-23
		(c) If relevant, consider translating estimates of relative risk into absolute risk for a meaningful time period<br>**N/A**	-
Other analyses	17	Report other analyses done—eg analyses of subgroups and interactions, and sensitivity analyses<br>**DONE – See Results section**	20-21
**Discussion**			
Key results	18	Summarise key results with reference to study objectives<br>**DONE – see Discussion section**	23-26
Limitations	19	Discuss limitations of the study, taking into account sources of potential bias or imprecision. Discuss both direction and magnitude of any potential bias<br>**DONE – see Discussion section**	26-27

For peer review only - http://bmjopen.bmj.com/site/about/guidelines.xhtml

Interpretation	20	Give a cautious overall interpretation of results considering objectives, limitations, multiplicity of analyses, results from similar studies, and other relevant evidence<br>**DONE – see Discussion section**	23-26
Generalisability	21	Discuss the generalisability (external validity) of the study results<br>**DONE – see Discussion section**	23-27
**Other information**			
Funding	22	Give the source of funding and the role of the funders for the present study and, if applicable, for the original study on which the present article is based<br>**DONE – see Funding section**	29

*Give information separately for exposed and unexposed groups.

Note: An Explanation and Elaboration article discusses each checklist item and gives methodological background and published examples of transparent reporting. The STROBE checklist is best used in conjunction with this article (freely available on the Web sites of PLoS Medicine at http://www.plosmedicine.org/, Annals of Internal Medicine at http://www.annals.org/, and Epidemiology at http://www.epidem.com/). Information on the STROBE Initiative is available at www.strobe-statement.org.

For peer review only
